# Computational Exploration of Ambiphilic Reactivity of Azides and Sustmann’s Paradigmatic Parabola

**DOI:** 10.1021/acs.joc.1c00239

**Published:** 2021-03-26

**Authors:** Pan-Pan Chen, Pengchen Ma, Xue He, Dennis Svatunek, Fang Liu, Kendall N. Houk

**Affiliations:** † Department of Chemistry and Biochemistry, University of California, Los Angeles, California 90095-1569, United States; ‡ College of Sciences, Nanjing Agricultural University, Nanjing 210095, China

## Abstract

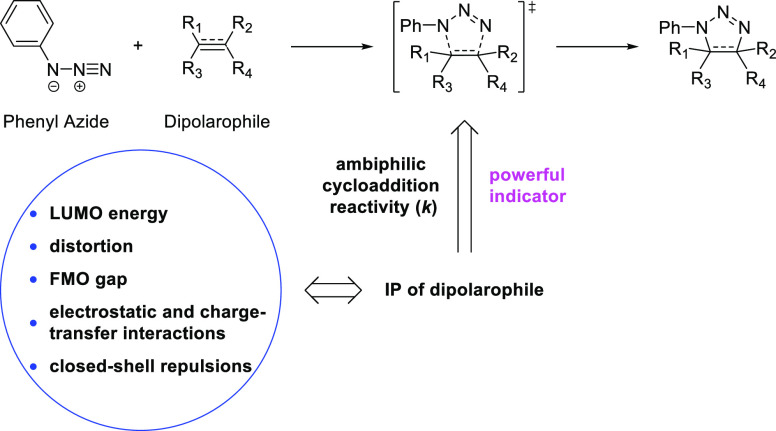

We examine the theoretical underpinnings of the seminal discoveries by Reiner Sustmann about the ambiphilic nature of Huisgen’s phenyl azide cycloadditions. Density functional calculations with ωB97X-D and B2PLYP-D3 reproduce the experimental data and provide insights into ambiphilic control of reactivity. Distortion/interaction-activation strain and energy decomposition analyses show why Sustmann’s use of dipolarophile ionization potential is such a powerful predictor of reactivity. We add to Sustmann’s data set several modern distortion-accelerated dipolarophiles used in bioorthogonal chemistry to show how these fit into the orbital energy criteria that are often used to understand cycloaddition reactivity. We show why such a simple indicator of reactivity is a powerful predictor of reaction rates that are actually controlled by a combination of distortion energies, charge transfer, closed-shell repulsion, polarization, and electrostatic effects.

## Introduction

**Figure 1:**
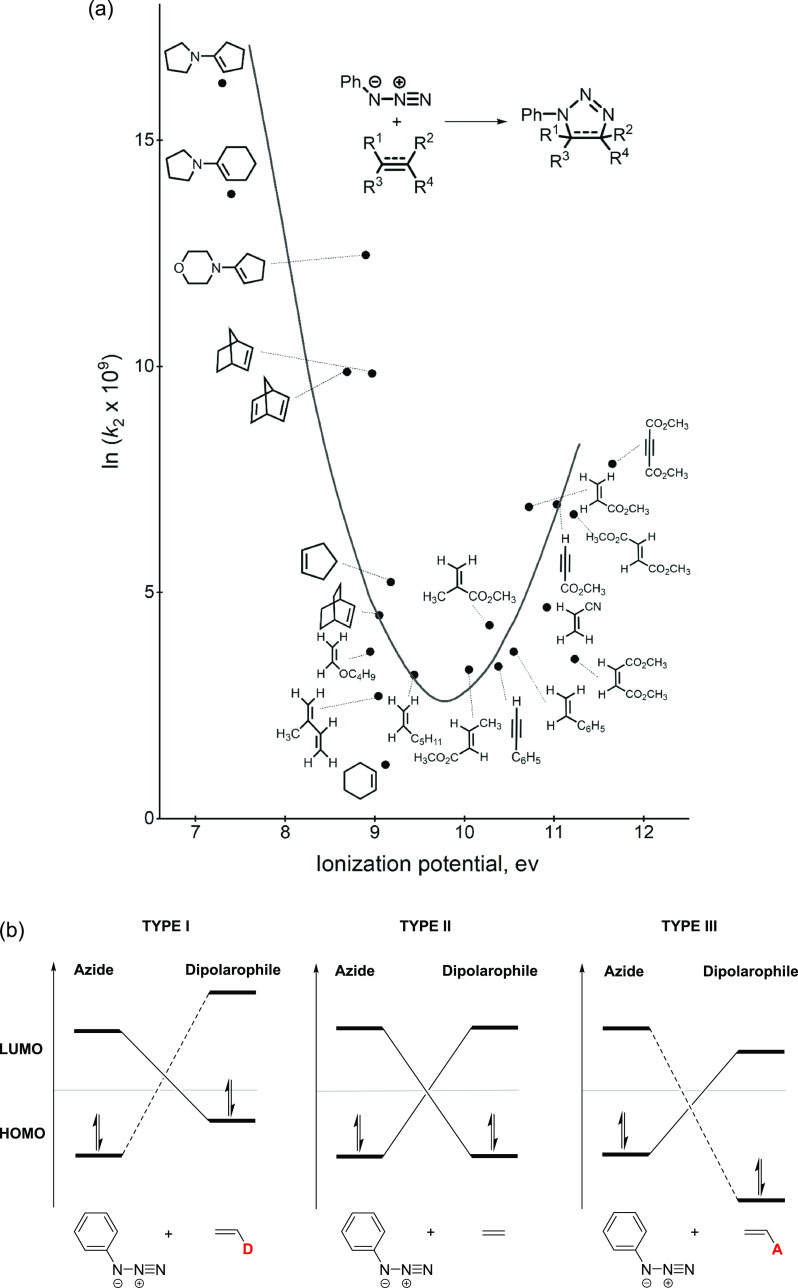
(a) Plot of reactivity (ln*k*_2_) vs IP. Replotted in the same style as Sustmann’s original figure in ref (1). Copyright 1972 by Verlag Chemie GmbH, Germany. (b) Schematic of the azide HOMO and LUMO vs those of donor-substituted dipolarophiles, ethylene, and acceptor-substituted dipolarophiles.

Sustmann and Trill published a landmark paper in 1972 about reactivity of phenyl azide in 1,3-dipolar cycloadditions.^1a^ At that time, an iconic parabola (Figure 1a) presented for the first time a memorable summary of how the electronic nature of a dipolarophile, relative to that of the 1,3-dipole, determines the rate of reaction. This parabola, based on second-order perturbation theory,^2^ shows the correlation of the second-order rate constant *k* for cycloadditions of phenyl azide to a series of dipolarophiles and the experimental ionization potential (IP) of these dipolarophiles.^1b^ The IP is related by Koopmans’ theorem to the negative of the highest occupied molecular orbital (HOMO) energy and serves as a measure of both the HOMO and lowest unoccupied molecular orbital (LUMO) energies: a low-energy HOMO is usually accompanied with a low-energy LUMO and vice versa.

Figure 1b shows how donor (D) and acceptor (A) substituents alter the frontier molecular orbital (FMO) energies of a dipolarophile.^1c^ In the reaction of phenyl azide with ethylene (Figure 1b, middle), two sets of interactions contribute: the HOMO of azide with the LUMO of ethylene and the HOMO of ethylene with the LUMO of azide. An electron donor (Figure 1b, left) raises the ethylene HOMO and LUMO energies, which leads to better interaction between the HOMO of ethylene and the LUMO of azide, resulting in stronger stabilization and higher reactivity. An electron acceptor (Figure 1b, right) enhances the reactivity of ethylene in the same manner by lowering the HOMO and LUMO energies. With this FMO model, Sustmann qualitatively explained the effect of different substituents on reactivities in 1,3-dipolar cycloadditions.

The Sustmann paper quantified what we now describe as the ambiphilic character of aryl and alkyl azides, a feature that was revealed by comparisons of Huisgen’s experimental measurements^3^ to the many ionization potentials that became available from Heilbronner et al.^4^ and Sustmann et al.’s work,^5^ as well as ours^6^ and others^7^ through the commercialization of the PerkinElmer UV photoelectron spectrometer in 1967.^8^

Ever since that time, Fukui’s FMO theory has provided a powerful model for a qualitative understanding of reactivity and regioselectivity in reactions of various 1,3-dipoles with alkenes.^9^ We recently proposed a general distortion/interaction (D/I) model for cycloaddition reactivity,^10a−10d^ and Bickelhaupt proposed the equivalent activation strain (AS) model.^10e,10f^ The D/I-AS model has been applied to 1,3-dipolar cycloadditions and shows that there are correlations between distortion energies and the activation barriers for 1,3-dipolar cycloadditions of various 1,3-dipoles with alkenes and alkynes. The reactivity differences between dipoles are often controlled by the distortion energies of the 1,3-dipoles (the energy required to distort the ground state of the 1,3-dipoles to their transition state geometries), rather than by FMO interactions or reaction thermodynamics. However, the reactivity of dipolarophiles with a given dipole correlates with orbital interactions and can be understood with FMO analysis.^10a^ Energy decomposition analyses have shown that a variety of other factors, like closed-shell repulsion, electrostatic effects, and polarization, may also influence reactivity.^10e^ A different approach based on electron density analyses was proposed by Domingo.^11^ Domingo’s approach, molecular electron density theory (MEDT), is based on conceptual density functional theory, in which reactivity parameters are obtained by computations of changes in electron density upon electron removal or addition. This obviously ties in closely with Sustmann’s ionization potential as an indicator of reactivity, although Domingo criticizes severely any discussion of reactivity in terms of orbitals.^11^ The MEDT approach was applied to azide cycloadditions and many other cycloadditions.^11b^ In addition, Cremer et al. performed computational studies of ten different cycloadditions of 1,3-dipoles XYZ (diazonium betaines, nitrilium betaines, azomethines, and nitryl hydride) to acetylene with B3LYP/6-31G(d,p) geometries and CCSD(T)-F12/aug-cc-pVTZ energetics, employing their unified reaction valley approach (URVA)^12^ on the concerted processes, and Breugst, with Huisgen and Reissig, conducted calculations with M06-2X geometries and DLPNO-CCSD(T)^13^ energies for the concerted 1,3-dipolar cycloadditions of diazomethane with many model-substituted alkynes.^14^ Because few recent high-accuracy calculations have explored the full range of azide cycloadditions to the alkene dipolarophiles studied experimentally by Huisgen, we carried out an analysis of the Sustmann relationship for this important class of reactions.^15^

## Computational Methods

All density functional theory (DFT) calculations were performed with Gaussian 16.^16^ Geometry optimizations of all stationary points were performed with the long-range corrected hybrid functional, ωB97X-D^17^ with the 6-31+G(d,p) basis set in solution. Solvents were chosen according to what was used in the experiment. Vibrational frequencies were calculated at the same level of theory to evaluate the zero-point vibrational energy (ZPVE) and thermal corrections at 298.15 K. The single-point energies were computed using ωB97X-D and the double hybrid B2PLYP-D3^18^ functional with the aug-cc-pVTZ^19^ basis set; solvation energy corrections were evaluated with the CPCM model.^20^ The Hirshfeld charges of the transition states were analyzed at the ωB97X-D/6-31+G(d,p) level of theory on the optimized structures to determine the direction and extent of charge transfer. Frontier molecular orbitals were calculated on DFT-optimized structures at the HF level of theory with the 6-31G(d) basis set and visualized with Multiwfn^21^ and VMD.^22^ Fragment distortion and interaction energies were calculated with autoDIAS^23^ at the ωB97X-D level of theory with the aug-cc-pVTZ basis set in the gas phase. Energy decomposition analyses were performed in ADF 2019.304^24^ at the ωB97X-D3/TZ2P^25^ level of theory using PyFrag 2019.^26^ The orbital coefficients were calculated using the NBO^27^ module of Gaussian 16 and Multiwfn. Extensive conformational searches for the intermediates and transition states have been carried out to ensure that the lowest energy conformers were located. The 3D images of molecules were generated using CYLView.^28^

## Results and Discussion

We investigated the reactivity of phenyl azide in the context of modern density functional theory and the distortion/interaction-activation strain model and analyzed the interactions of phenyl azide and dipolarophiles with an energy decomposition analysis. We describe the factors that control reactivity and show why Sustmann’s parabola provides such a powerful model for ambiphilic cycloaddition reactivity, despite the many factors that contribute to reactivity.

**Figure 2:**
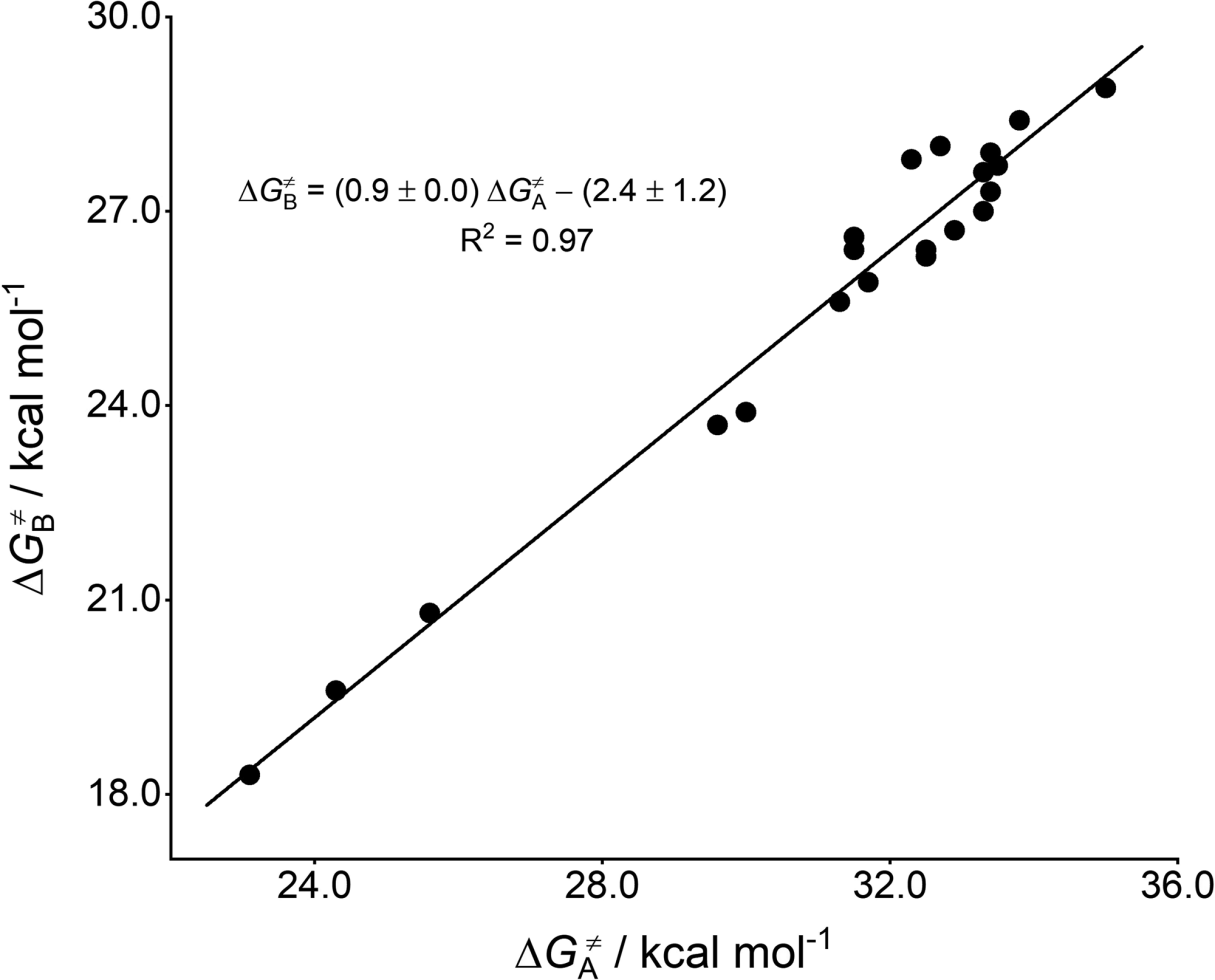
Correlation of *^a^*Δ*G*_A_^‡^ with *^b^*Δ*G*_B_^‡^ for the cycloadditions between phenyl azide and 21 dipolarophiles. *^a^*ωB97X-D/aug-cc-pVTZ-CPCM(solvent)//ωB97X-D/6-31+G(d,p)-CPCM(solvent); *^b^*B2PLYP-D3/aug-cc-pVTZ-CPCM(solvent)//ωB97X-D/6-31+G(d,p)-CPCM(solvent).

**Figure 3:**
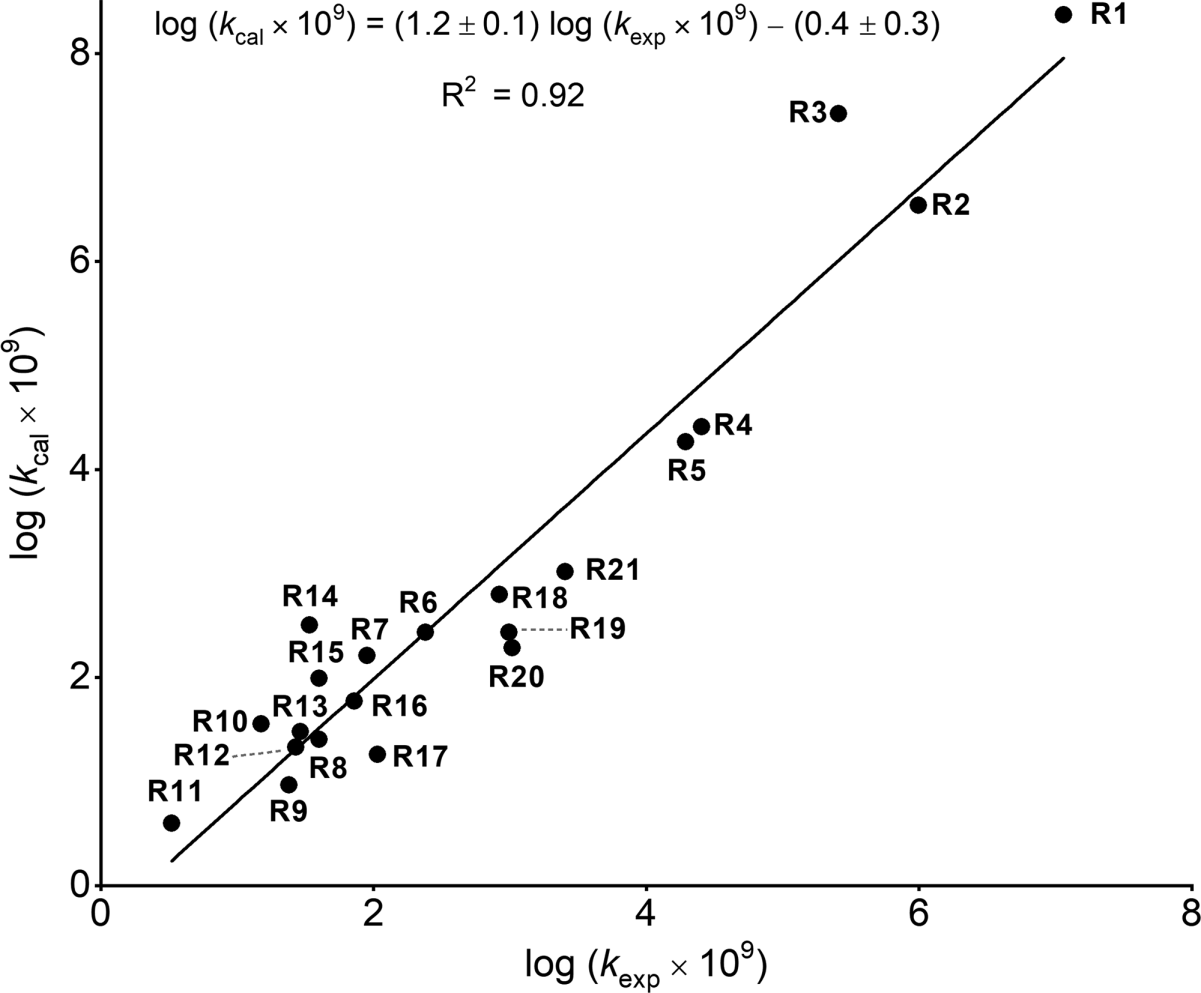
Correlation of logarithms of the experimental rate constants with the calculated rate constants (method B) for the cycloadditions between phenyl azide and 21 dipolarophiles.

**Scheme 1:**
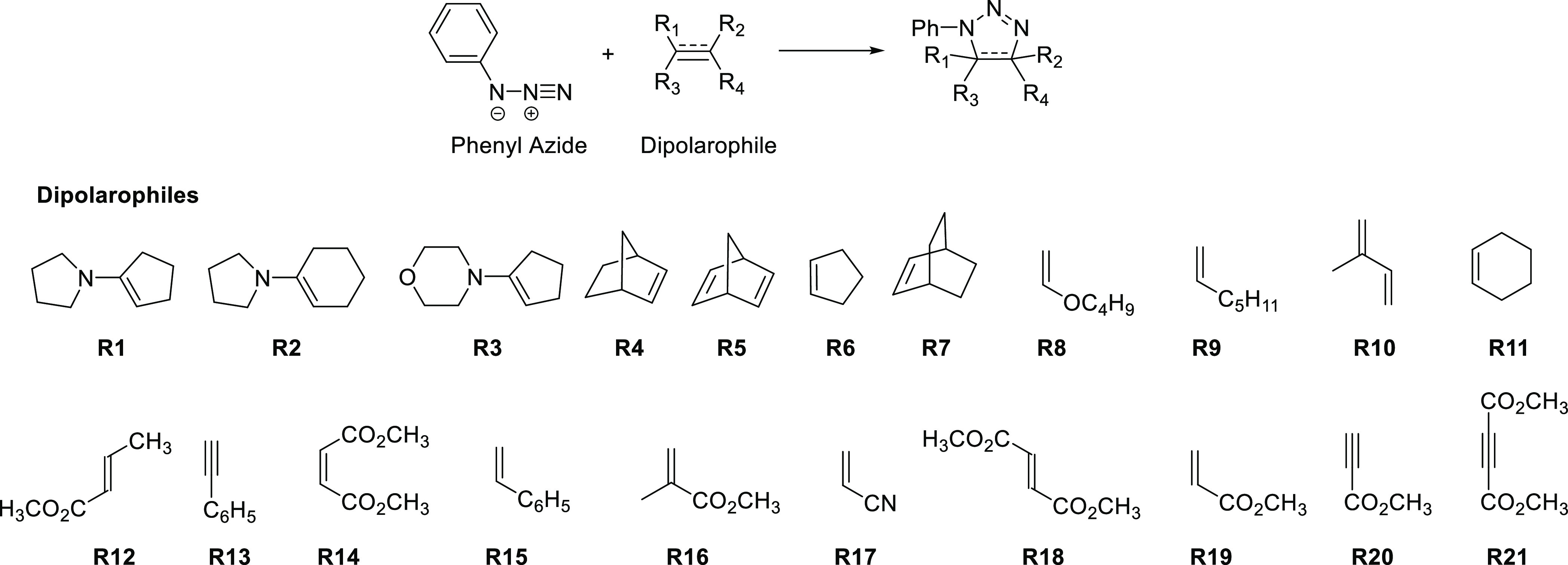
The 1,3-Dipolar Cycloaddition between Phenyl Azide and Different Dipolarophiles Studied by Sustmann and in This Paper

We computed the transition states of the 1,3-dipolar cycloadditions between phenyl azide and 27 dipolarophiles. The first 21 dipolarophiles, which are included in Sustmann’s plot, are listed in Scheme 1. Table 1 shows the energetics computed with two functionals (method A and method B). The same trend of reactivity was obtained from the two methods (Figure 2). The computed rate constants (by applying the Eyring equation to activation free energies)^29^ given by the double hybrid functional (method B) agree somewhat better with measured rate constants^30^ (Table 1 and Figure 3). For all dipolarophiles studied, there is a good linear correlation between the variables. In the following discussion, we report the values of energetics calculated by B2PLYP-D3, unless otherwise specified.

**Table 1:** Experimental Rate Constants^a^ and Calculated Reaction Barriers and Rate Constants^b^

		method A^c^		method B^d^	
dipolarophile	log(*k*_exp_ × 10^9^)	Δ*G*_A_^‡^ (kcal/mol)	log(*k*_cal_ × 10^9^)	Δ*G*_B_^‡^ (kcal/mol)	log(*k*_cal_ × 10^9^)
**R1**^e^	7.06	23.1	4.85	18.3	8.37
**R2**^e^	6.00	25.6	3.02	20.8	6.54
**R3**^e^	5.41	24.3	3.97	19.6	7.42
**R4**^e^	4.40	29.6	0.09	23.7	4.41
**R5**	4.29	30.0	–0.21	23.9	4.27
**R6**^e^	2.38	32.5	–2.04	26.4	2.43
**R7**	1.95	32.9	–2.33	26.7	2.21
**R8**	1.60	32.3	–1.90	27.8	1.41
**R9**	1.38	33.8	–3.00	28.4	0.97
**R10**	1.18	33.3	–2.63	27.6	1.55
**R11**	0.52	35.0	–3.88	28.9	0.60
**R12**	1.43	33.4	–2.70	27.9	1.33
**R13**	1.46	33.5	–2.78	27.7	1.48
**R14**	1.53	32.5	–2.04	26.3	2.51
**R15**	1.60	33.3	–2.63	27.0	1.99
**R16**	1.86	33.4	–2.70	27.3	1.77
**R17**	2.03	32.7	–2.19	28.0	1.26
**R18**	2.92	31.7	–1.46	25.9	2.80
**R19**	2.99	31.5	–1.31	26.4	2.43
**R20**	3.02	31.5	–1.31	26.6	2.29
**R21**	3.40	31.3	–1.16	25.6	3.02

a For different dipolarophiles, the corresponding cycloaddition was performed in a particular solvent (carbon tetrachloride or benzene) at 25 °C.^30^

b For different dipolarophiles, the calculated reaction barriers and rate constants are values in a specific solvent (carbon tetrachloride or benzene), which was chosen according to what was used in the experiment.^30^

c ωB97X-D/aug-cc-pVTZ-CPCM(solvent)//ωB97X-D/6-31+G(d,p)-CPCM(solvent).

d B2PLYP-D3/aug-cc-pVTZ-CPCM(solvent)//ωB97X-D/6-31+G(d,p)-CPCM(solvent).

e In the experimental study, the reaction solvent was benzene for these dipolarophiles and carbon tetrachloride for the other dipolarophiles.^30^

We compared our predicted ionization potentials (IP) based on Koopmans’ theorem^31^ to those measured experimentally by photoelectron spectroscopy and found that the theoretical value is in good agreement with the experimental value (Figure S1). In Sustmann’s plot, a key concept is that the HOMO and LUMO of the substituted dipolarophiles will be shifted in the same direction. Therefore, the variation of HOMO energy should reflect corresponding shifts of LUMO energy. We indeed found a correlation between predicated ionization potential and electron affinity (EA) for different dipolarophiles (Figure S2), indicating that the change of LUMO energy can be semi-quantitatively characterized by the variation of HOMO energy.

**Figure 4:**
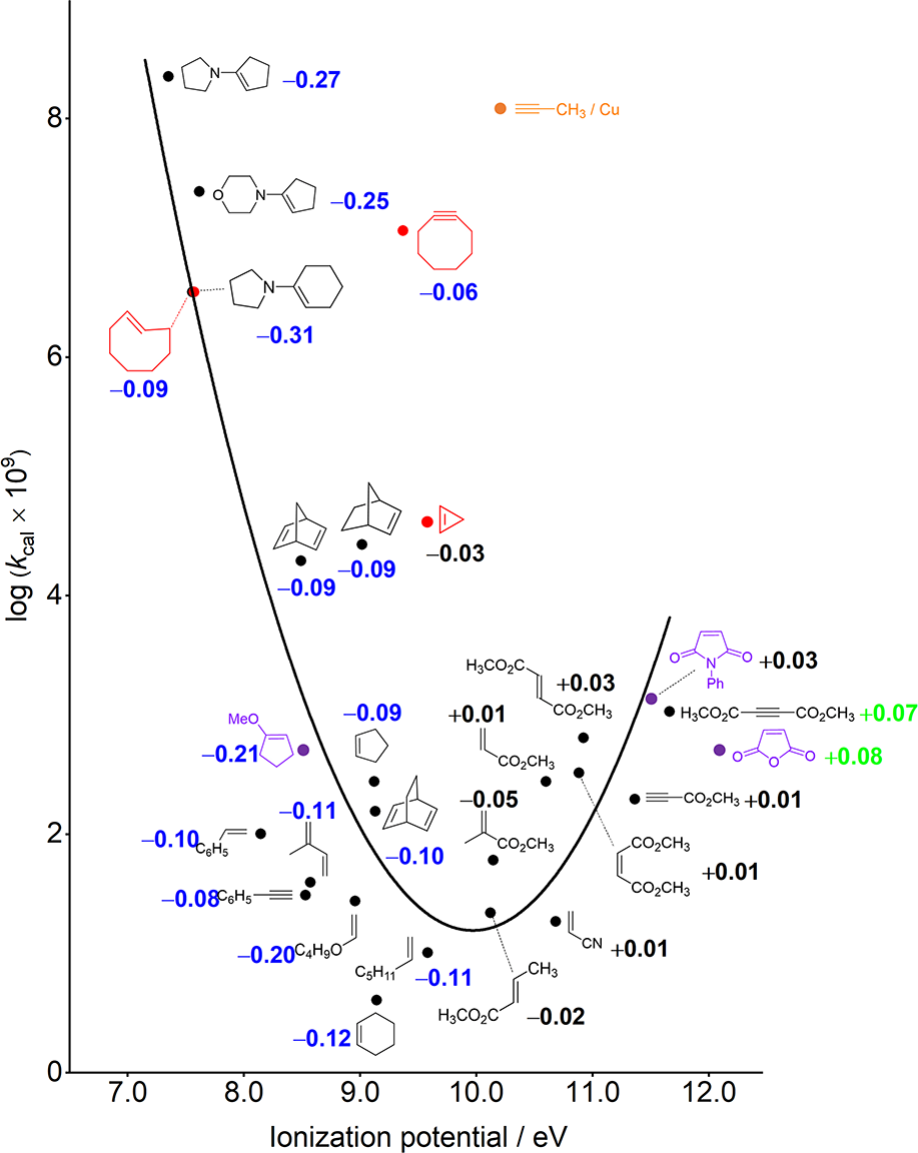
Theoretical version of Sustmann’s plot with charge transfer (e), using B2PLYP-D3/aug-cc-pVTZ-CPCM(solvent)//ωB97X-D/6-31+G(d,p)-CPCM(solvent)-predicted rate constants and HF/6-31G(d)-calculated ionization potentials (eV). The three purple points, for which experimental rate constants have been given in Huisgen’s experimental study^30^ but were not included in Sustmann’s analysis, are qualitatively in line with the parabolic trend. The three points in red are added to the original plot to show how distortion-accelerated dipolarophiles fit on the correlation, and the point in orange is added to the original plot to show the enormous acceleration that occurs with Cu catalysis. Data in blue (negative number) shows charge transfer from dipolarophiles to azide; data in green (positive number) shows charge transfer from azide to dipolarophiles; data in black shows charge transfer of 0.05 e or less between reactants, essentially zero.

We also plotted the predicted log *k* values for the 1,3-dipolar cycloadditions versus the calculated ionization potentials of a number of dipolarophiles. Figure 4 is a theoretical version of Sustmann’s plot. The parabolic relationship of reactivity with dipolarophile ionization potential is nicely consistent with Sustmann’s results.^1a^ Additionally, we plotted the relationship between logarithms of the predicted rate constants of 1,3-dipolar cycloadditions and calculated Mulliken electronegativities (χ) for all dipolarophiles, and we obtained a similar parabola (Figure S3). This indicates that there is a relationship between the ionization potential used by Sustmann and the electronegativity, which is the average of the ionization energy and the electron affinity (Figure S4). It is worth noting that the correlation with Mulliken’s electronegativity is only a slight modification of the poor correlation between IP and EA (compare Figures S2 and S4) because Mulliken’s electronegativties (χ = IP/2 + EA/2) superimpose half of the EA values on the identity correlation IP/2 vs IP and thus reduce the magnitude of the deviations while simultaneously increasing the slope.

We have, in addition to the 21 dipolarophiles reported in Sustmann’s analysis, studied the cycloadditions of phenyl azide with other dipolarophiles, such as 1-methoxycyclopentene,^32^ maleic anhydride, and *N*-phenylmaleimide, for which experimental rate constants were given in Huisgen et al.’s 1967 paper.^30^ The reactivities for these three species (Figure 4, purple) are qualitatively in line with the parabolic trend. Moreover, we have incorporated the results for distortion-activated (or strain-promoted) dipolarophiles,^33^ e.g., *trans*-cyclooctene, cyclooctyne, and cyclopropene, which are of interest in bioorthogonal chemistry (Figure 4, red).^34^ It is shown that *trans*-cyclooctene actually fits very nicely on the parabola since the distortion of the alkene increases the HOMO energy to be about the same as pyrrolidinocyclohexene (**R2**). However, cyclooctyne has a slightly lowered ionization potential (9.4 eV) as compared to 2-butyne (9.6 eV)^35^ but is anomalously reactive, as expected for a distortion-accelerated dipolarophile.^33,36^ Cyclopropenes have been used as dienophiles in bioorthogonal chemistry by the Prescher^34d^ and Devaraj groups^34e^ and studied theoretically.^34f^ Cyclopropene is somewhat more reactive than reactive norbornene. To support our statement that *trans*-cyclooctene, cyclooctyne, and cyclopropene are all distortion-promoted dipolarophiles, we performed distortion/interaction-activation strain analysis on the transition states of their cycloaddition reactions with phenyl azide (Figure S5). The corresponding results confirmed our inference (Table S1). Finally, we add the famous Sharpless Cu-catalyzed click reaction of azide with terminal alkyne (Figure 4, orange) to the graph to emphasize the enormous acceleration that occurs with Cu catalysis.^37^

We have also calculated the charge transfer for all cycloaddition transition states, and values are shown in Figure 4. In general, the fast reactions involve significant (δ*q* > 0.05 e) charge transfer, which is consistent with the general idea that orbital interactions are influential on reactivity.

**Figure 5:**
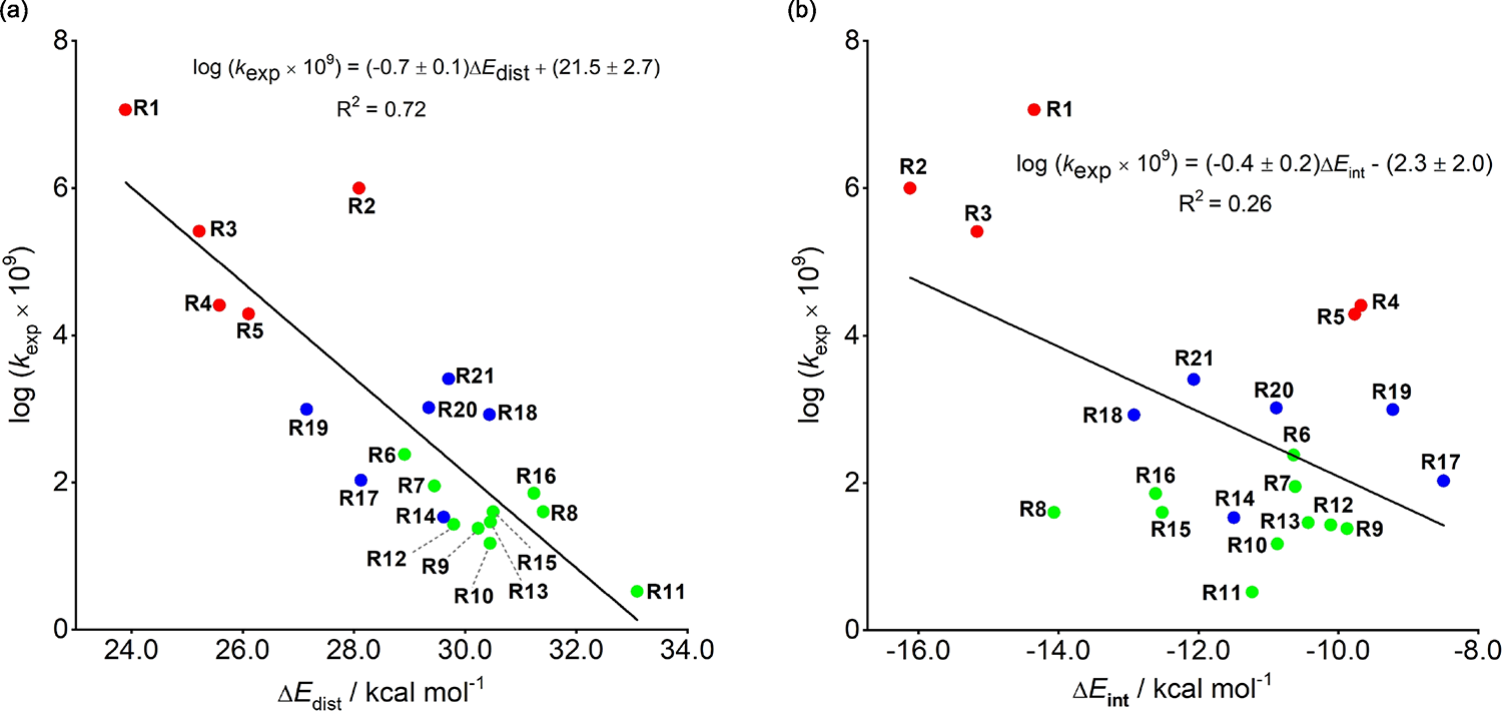
Plots of logarithms of the experimental rate constants versus (a) distortion and (b) interaction energies for the cycloadditions between phenyl azide and dipolarophiles. Energies are in kcal/mol.

We performed distortion/interaction-activation strain analyses on these cycloaddition transition states to evaluate other factors that influence reactivities. This involves decomposition of the electronic energy (Δ*E*) into two terms: the distortion energy (Δ*E*_dist_) that results from the distortion of the individual reactants and the interaction (Δ*E*_int_) between the deformed reactants. Based on our study, we find that there is a rough relationship between distortion energy and cycloaddition reactivity (Figure 5a). However, the interaction energy surprisingly has little correlation with the reactivity (Figure 5b). This is, of course, in apparent contrast to Sustmann’s conclusion and that from charge transfer assessments that suggest that FMO interactions control cycloaddition reactivity. As shown in Figure 5a, nucleophilic dipolarophiles (**R1**–**R5**, red dots) show low distortion energies and higher reactivities; electrophilic dipolarophiles (**R14** and **R17**–**R21**, blue dots) show moderate distortion energies and reactivities; neutral or ambiphilic dipolarophiles (**R6**–**R13**, **R15**, and **R16**, green dots) show the greatest distortion energies and the lowest reactivities. The cycloaddition reactivity as a whole is related to the distortion energy. There is a close relationship between FMO interactions, the position of the transition state, and distortion energies. For nucleophilic dipolarophiles, the HOMO-LUMO interaction is stronger (smaller FMO gap), so the transition state is earlier, corresponding to less distortion. For electrophilic dipolarophiles, the HOMO-LUMO interaction is weak (larger FMO gap), so the transition state is later, corresponding to greater distortion. In other words, the orbital interactions are related to the transition state position and therefore the distortion energies. Although Sustmann’s parabola provided a great qualitative guide to the reactivity trends for an ambiphilic 1,3-dipole, it is because the IP reflects not only changes in HOMO or LUMO energy but also distortion energy differences.^38^

**Figure 6:**
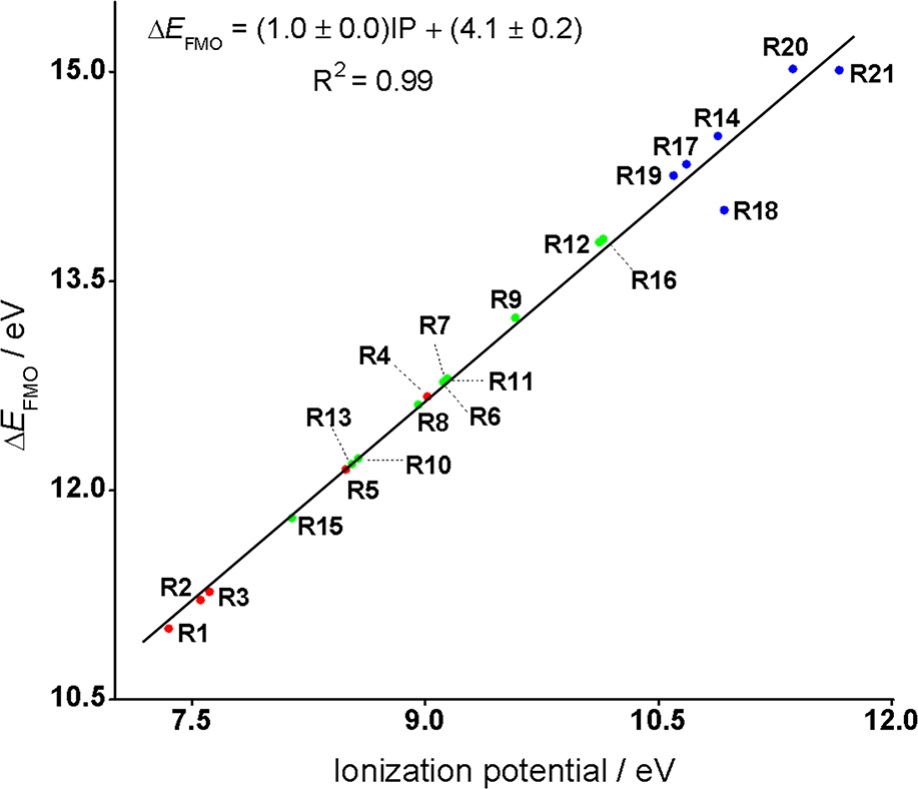
Plot of optimum FMO gaps (eV) between dipole and dipolarophiles versus calculated ionization potentials (eV) of a number of dipolarophiles.

Figure 6 shows a plot of the optimum FMO gaps between dipole and 21 dipolarophiles (the FMOs used here are those involved in the formation of new bonds, not the actual HOMOs and LUMOs) versus the calculated ionization potentials of these dipolarophiles. There is an excellent linear correlation between IP and the energy gap. For the electron-rich (**R1**–**R5**, red dots) and neutral or ambiphilic dipolarophiles (**R6**–**R13**, **R15**, and **R16**, green dots), lower IP and a smaller FMO gap correlate with higher reactivity. However, with the electron-deficient dipolarophiles, **R14** and **R17**–**R21** (blue dots), there must be other factors that compensate the unfavorable FMO gaps, leading to high reactivity.

A plot of the IP of the dipolarophiles versus both FMO gaps (the FMOs used here are those involved in the formation of new bonds, not the actual HOMOs and LUMOs) is provided in Figure S6. As expected, the HOMO_dipolarophile_-(LUMO+2)_azide_ gap of electron-rich dipolarophiles (IP < 10 eV) accounts for the left arm of the parabola. For dipolarophiles with IP around 10 eV, HOMO_dipolarophile_-(LUMO+2)_azide_ and (HOMO-3)_azide_-LUMO_dipolarophile_ gaps become comparable; for dipolarphiles with IP greater than 11 eV, the (HOMO-3)_azide_-LUMO_dipolarophile_ gap becomes determinant and corresponds with the right arm of the parabola. This is the basic idea that Sustmann proposed and is now quantitatively shown in Figure S6.

**Figure 7:**
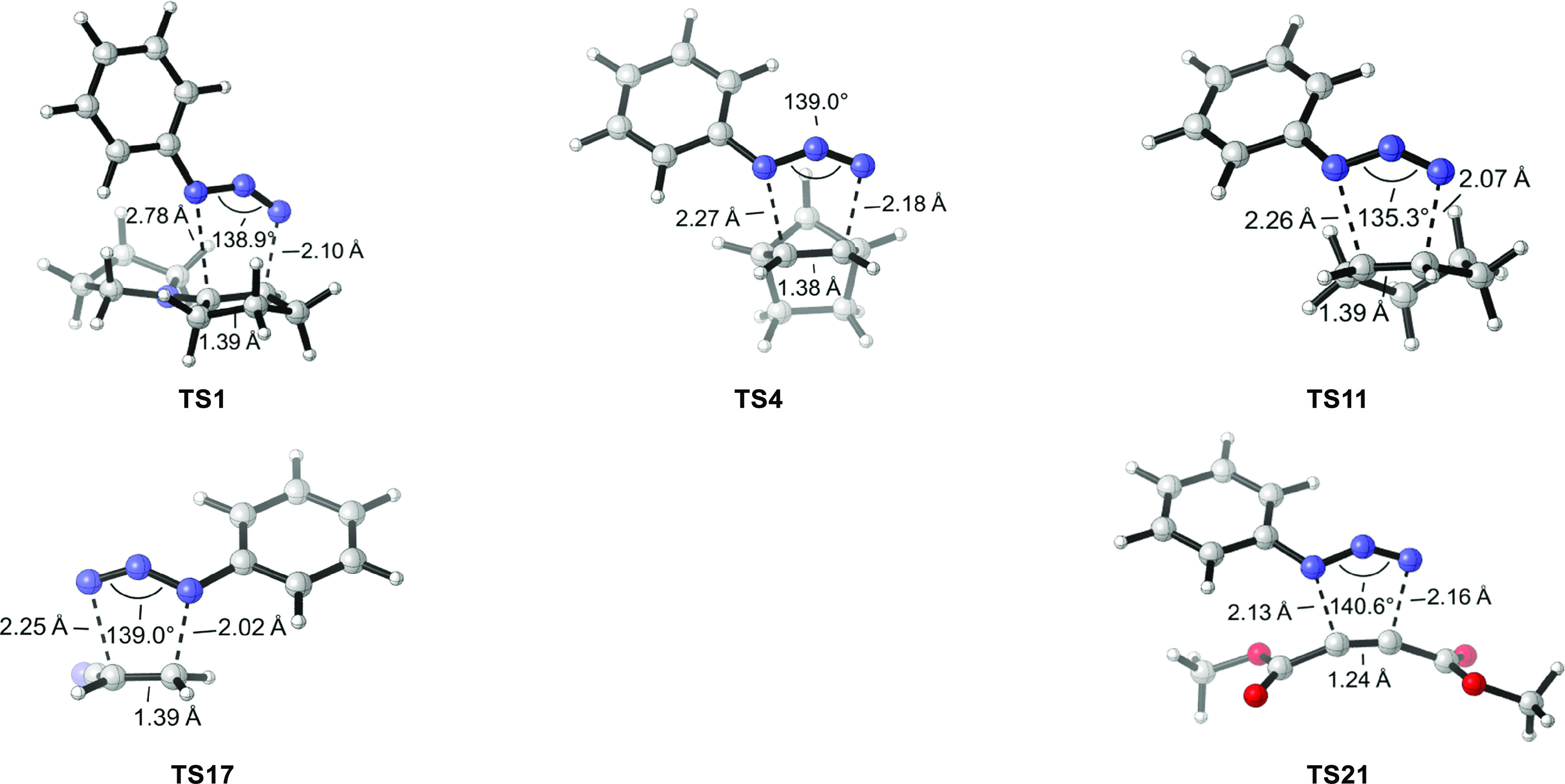
DFT-optimized transition structures for cycloadditions between phenyl azide and five representative dipolarophiles. **TS1** and **TS17** are for the experimentally observed regioselectivity.

To gain more insights into the physical factors leading to the reactivity trend, we studied the cycloadditions between phenyl azide and five representative dipolarophiles, including the most reactive and electron-rich **R1** and **R4**, neutral **R11**, and electron-deficient **R17** and **R21**. The corresponding transition states are shown in Figure 7. The forming C–N bond ranges from 2.0 to 2.8 Å, and the transition state structure varies from nearly synchronous to highly asynchronous, with an average of the two forming C–N bonds being 2.2–2.4 Å.

**Figure 8:**
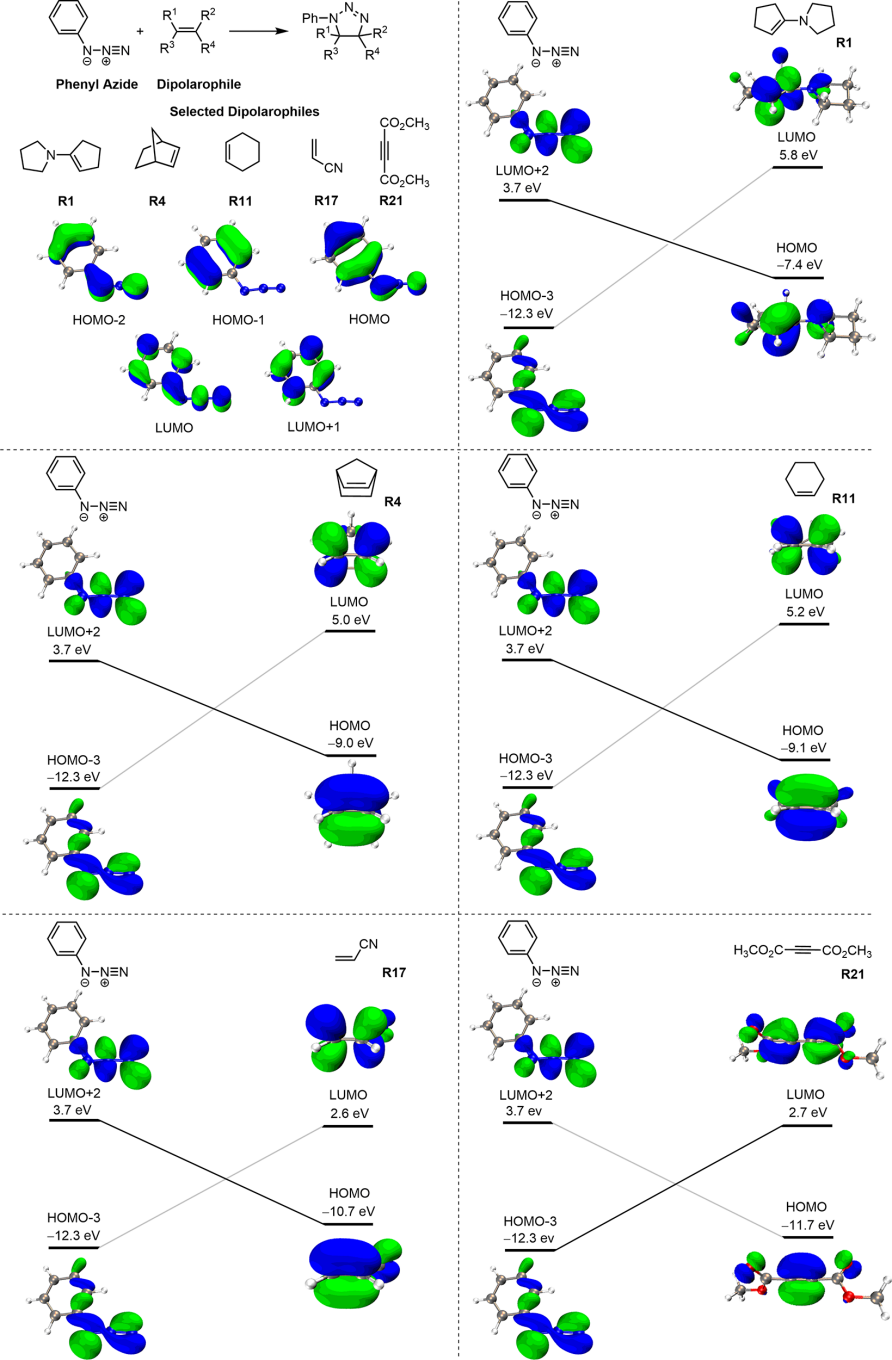
Orbital interaction diagrams for the cycloadditions between phenyl azide and selected dipolarophiles. In order to justify that HOMO-3 and LUMO+2 of phenyl azide are the effective orbitals involved in the orbital interactions between phenyl azide and dipolarophiles, we show the HOMO, HOMO-1, and HOMO-2 as well as the LUMO and LUMO+1 of phenyl azide in the upper left corner for comparison.

Figure 8 shows the orbital interaction diagrams of the selected reactions. As noted above, these diagrams show the FMOs that are involved in the formation of new bonds, not the actual HOMOs and LUMOs. For the cycloaddition between phenyl azide and **R1**, the dominant orbital interaction is between the HOMO of **R1** and the LUMO+2 of phenyl azide (the LUMO+2 is slightly higher than the perpendicular LUMO, which conjugates with the phenyl group and is not involved in bond formation). This is the same with **R4**, **R11**, and **R17**. Along the series, as the HOMO energy of a dipolarophile decreases (the nucleophilicity of the dipolarophile decreases), the energy gap increases (11.1, 12.7, 12.8, and 14.4 eV for **R1**, **R4**, **R11**, and **R17**, respectively). For **R21**, the interaction between HOMO-3 of phenyl azide (the higher energy orbitals are not involved in bond formation) and the LUMO of **R21** dominates. Phenyl azide is somewhat electron-deficient and thus gives stronger interactions and faster rates with the electron-rich dipolarophiles.

**Figure 9:**
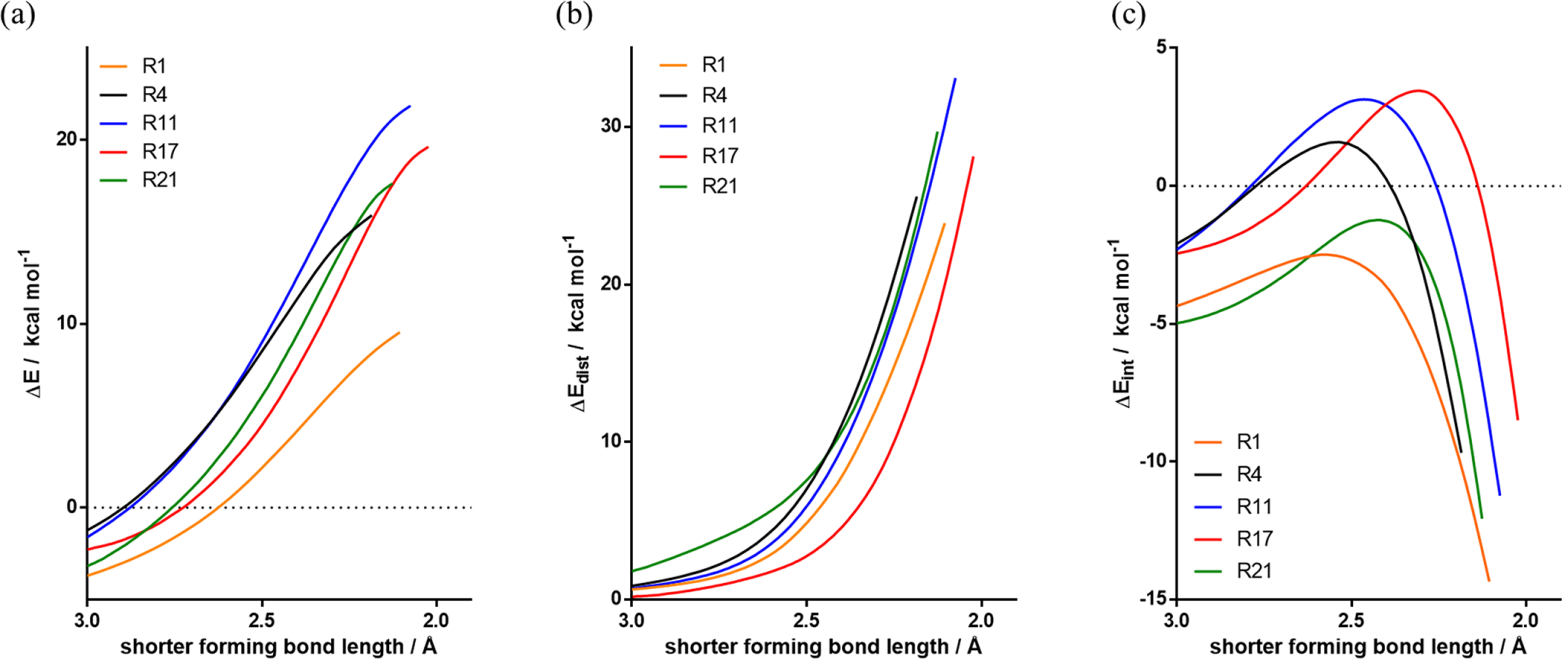
Distortion/interaction-activation strain (DIAS) analysis for selected cycloaddition reactions. Reactant distortion and interaction energies were calculated at the ωB97X-D level of theory with the aug-cc-pVTZ basis set, in the gas phase. (a) Electronic energies (Δ*E*). (b) Distortion energies (Δ*E*_dist_). (c) Interaction energies (Δ*E*_int_).

A distortion/interaction-activation strain analysis was performed along the reaction coordinate (defined by the bond length of the shorter forming C–N bond) for the five reactions in discussion (Figure 9). The transition states of cycloadditions between phenyl azide and symmetric dipolarophiles (**R4**, **R11**, and **R21**) are nearly synchronous, corresponding to generally higher distortion energies. Asymmetric dipolarophiles (**R1** and **R17**) have asynchronous transition states (bond formation mainly at one center) and lower distortion energies at a given forming bond length (Figure 9b). We interpret this as a result of the stronger HOMO-LUMO interactions in the asynchronous transition states: strong asynchronicity is related to lower distortion energies and to much greater overlap at the shorter forming bond.

The interaction energies (Figure 9c) are higher for very electron-rich enamine (**R1**) and very electron-deficient dimethyl acetylenedicarboxylate (**R21**). Interaction energies involve more than the commonly cited frontier orbital interactions represented in Figure 8, and we performed an energy decomposition analysis to determine the origins of the different interaction energies (Figure S7). The interaction energy in transition states (−8 to −14 kcal/mol) is a combination of Pauli repulsion (70 to 100 kcal/mol), orbital interactions (around −40 kcal/mol), and electrostatic interactions (around −40 kcal/mol). No clear pattern in the energy components was found. The origin of these very large individual interactions is that covalent bonds are being formed and very large changes in potential and kinetic energies occur as a result. We caution against simple interpretations of the very large changes in all energy components that occur in partial covalent bond formation; nevertheless, details of the energy decomposition analysis are given in the Supporting Information (Figure S7).

**Figure 10:**
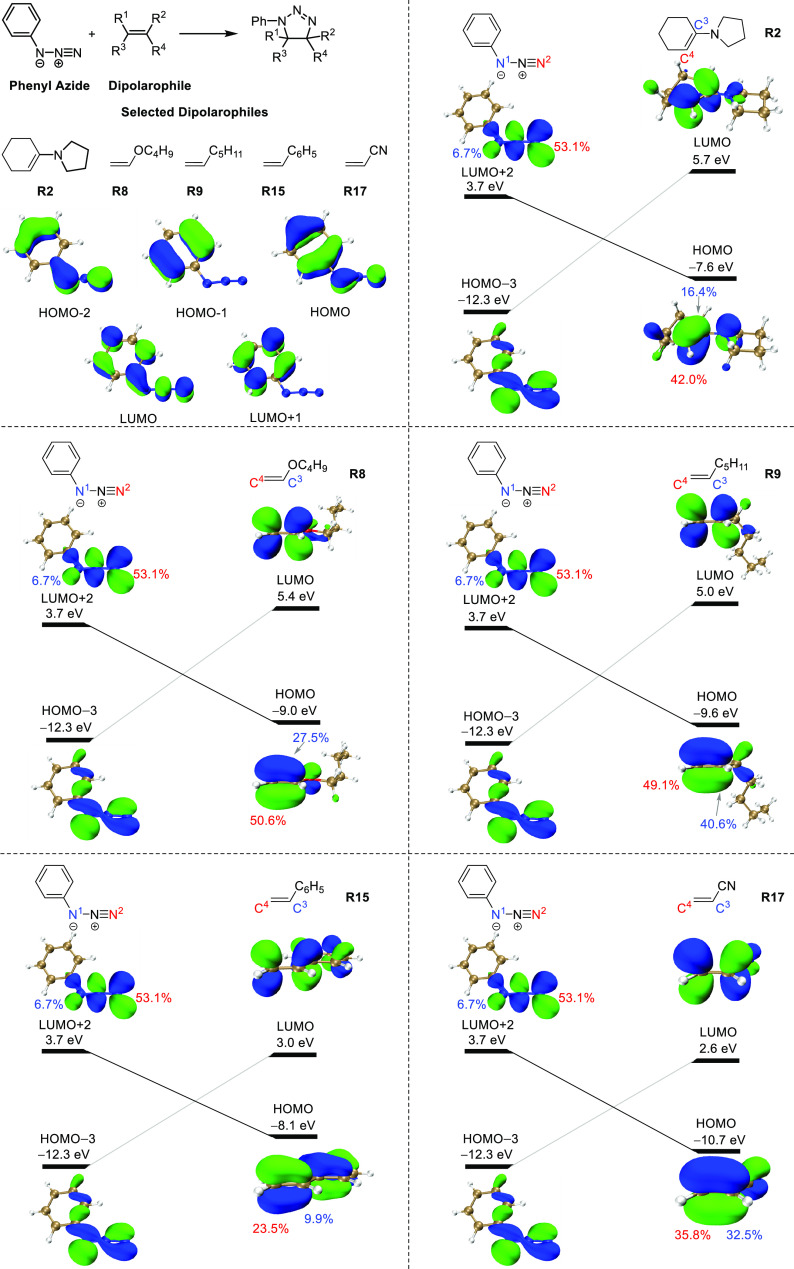
Orbital interaction diagrams for the cycloadditions between phenyl azide and selected dipolarophiles. We added orbital coefficients for the interacting orbitals. For phenyl azide, N^1^ is the nitrogen attached to the phenyl group, and N^2^ is the terminal-unsubstituted nitrogen. For dipolarophiles, C^3^ is the carbon attached to the substituent, and C^4^ is the terminal-unsubstituted carbon. In order to justify that HOMO-3 and LUMO+2 of phenyl azide are the effective orbitals involved in the orbital interactions between phenyl azide and dipolarophiles, we show the HOMO, HOMO-1, and HOMO-2 as well as the LUMO and LUMO+1 of phenyl azide in the upper left corner of Figure 10 for comparison.

We also investigated the regioselectivity of the 1,3-dipolar cycloadditions for five unsymmetrical alkenes, **R2**, **R8**, **R9**, **R15**, and **R17**. Orbital interaction diagrams for the corresponding cycloaddition reactions are shown in Figure 10. The regioselectivity has been rationalized traditionally by frontier molecular orbital interactions, and in general, the preferred product is indeed the one in which the favored product results from the union of the larger terminal coefficients of azide (the nucleophilic substituted terminus of azide in the HOMO-3 and the electrophilic unsubstituted terminus in the LUMO+2, see Figure 8) with the larger terminal coefficients in the complementary FMO of the dipolarophile.^9^

**Figure 11:**
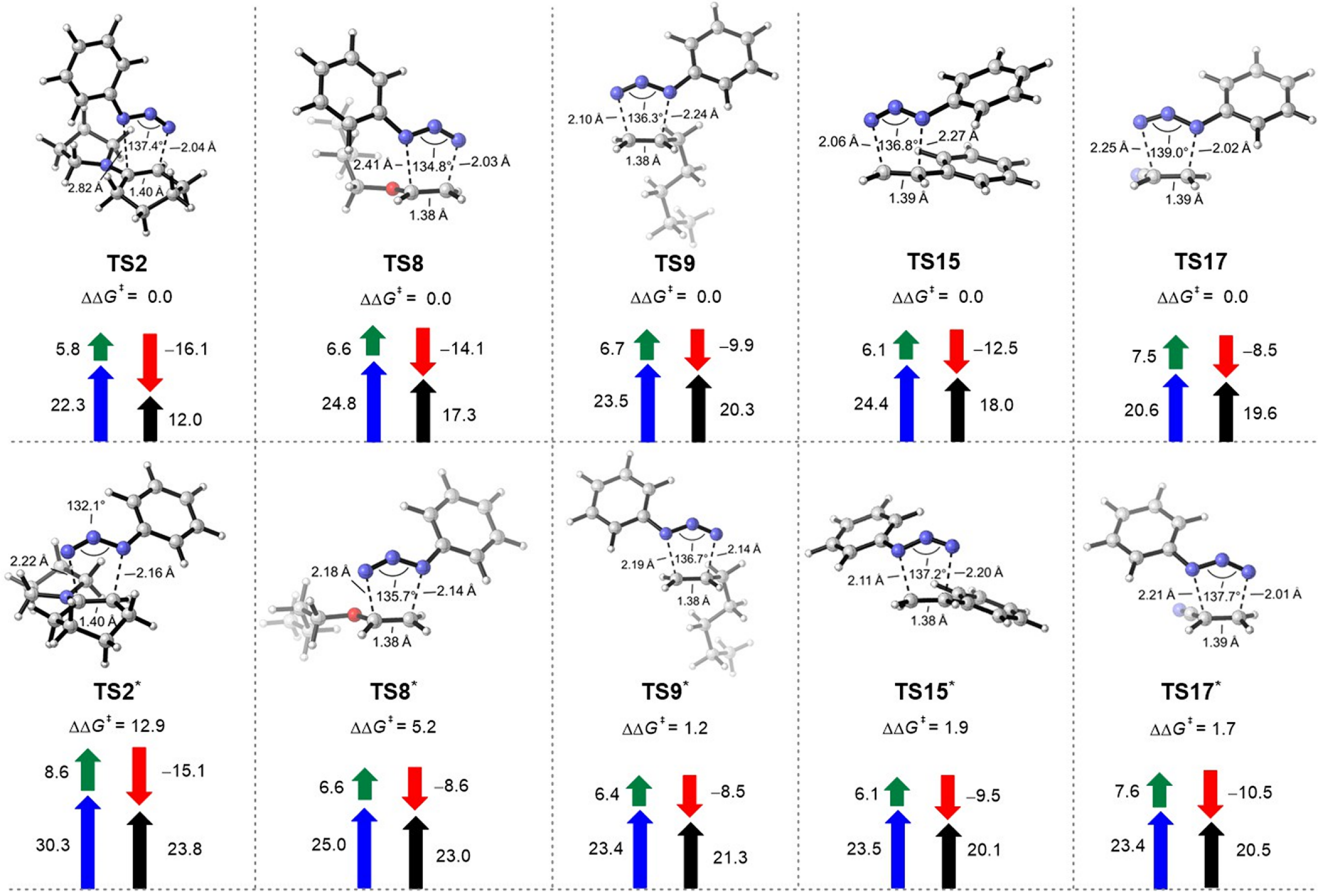
Distortion/interaction-activation strain (DIAS) analysis of the cycloaddition transition states to reveal the origins of regioselectivity. Free energies were calculated at the ωB97X-D/aug-cc-pVTZ-CPCM(solvent)//ωB97X-D/6-31+G(d,p)-CPCM(solvent) level of theory. Fragment distortion and interaction energies were calculated at the ωB97X-D level of theory with the aug-cc-pVTZ basis set, without the inclusion of solvation energy corrections (black, activation energies; blue, distortion energies of azide; green, distortion energies of dipolarophiles; red, interaction energies). Energies are in kcal/mol. The starred transition state is the regioisomeric transition state, which is unfavorable compared to the one without an asterisk.

To achieve a more quantitative view, we performed a distortion/interaction-activation strain analysis (Figure 11) to explore the origins of regioselectivity of the 1,3-dipolar cycloadditions for the five asymmetrical alkenes in discussion. For **R2**, both the distortion and interaction energies determine the regioselectivity. **TS2** is more asynchronous compared to TS2*. Therefore, it has lowered distortion energies for both azide and alkene. Additionally, there are more favorable interactions for **TS2** due to the orbital interactions (the combination between the terminal with the larger HOMO coefficient of alkene and the terminal with the larger LUMO+2 coefficient of azide leads to more favorable orbital interaction), corresponding to a larger charge transfer (Table S2, entry 1). Thus, the cycloaddition occurs via **TS2** with higher selectivity.

For **R8**, **R9**, and **R15**, it is the interaction energy that determines the regioselectivity. There are stronger interactions between phenyl azide and alkenes in the favorable transition states (**TS8**, **TS9**, and **TS15**) compared to unfavorable ones (TS8*, TS9*, and TS15*) due to orbital interactions, and correspondingly, more significant charge transfer occurs in favorable transition states (Table S2, entries 2–4). As for **R17**, it is the distortion of azide that determines the regioselectivity. **TS17** has a smaller distortion energy of azide compared to TS17*. In terms of interaction energy, TS17* is more favorable than **TS17** due to orbital interactions (Table S2, entry 5). However, the interaction energy contributes less to regioselectivity than the distortion energy. Therefore, the cycloaddition between phenyl azide and **R17** occurs via **TS17** selectively.

Based on the above analysis, we found that, in general, a combination of interaction energies and distortion energies parallels those interactions predicated by the FMO model, and the FMO models developed some time ago to rationalize and predict 1,3-dipolar cycloaddition regioselectivity are supported by this analysis.^9^

## Conclusions

As Sustmann showed in the 70s, the IP of dipolarophiles is a powerful indicator of reactivity. A low IP (high HOMO) indicates a very nucleophilic molecule, an electron-rich dipolarophile in our case, which reacts very rapidly with azide. A high IP suggests lowering of the HOMO (and LUMO at the same time); as the dipolarophile becomes more electrophilic, its reactivity with azide rises. We have also analyzed how the IP (minus of HOMO energy) relates to distortion, the FMO gap, closed-shell repulsions, and electrostatic and charge-transfer interactions. All of these are involved in controlling reactivity.

Distortion energies are related to the HOMO-LUMO gap of individual molecules through the “second-order Jahn–Teller effect”, which states that distortions are made easier by the mixing of the HOMO and LUMO of a molecule upon distortion; this becomes more effective when the HOMO-LUMO gap is small.^38^ A donor raises the HOMO energy (low IP), generally more than it raises the LUMO, which leads to a small HOMO-LUMO gap. Alternatively, an electron acceptor lowers the HOMO energy (high IP), generally less than it lowers the LUMO, which also narrows the HOMO-LUMO gap. Thus, for the dipolarophiles with either low or high IPs, the HOMO-LUMO gaps are small and distortion energies are small, at the same time that they are reactive nucleophiles or electrophiles.

Fukui’s frontier molecular orbital theory based only on HOMO-LUMO interactions is very successful, despite the variety of interactions that influence reactivity.^39^ The FMO gap is not only related to distortion energies but to electrostatic interactions as well. As orbital interactions increase, charge transfer increases, causing electrostatic interactions between the reactants to increase. Thus, the degree of electrostatic interactions is also related to charge transfer, which is the result primarily of FMO interactions.

Closed-shell repulsion is also related to FMO interactions. Strong HOMO-LUMO interactions between molecules are generally accompanied by low closed-shell repulsions between HOMOs of the two molecules. A reduction in closed-shell repulsion increases reactivity.^40^ The primary example of this relationship is found in the orbital symmetry relationships. An orbital symmetry-allowed reaction has strong HOMO-LUMO interactions between reactants because the HOMO of one molecule has the same symmetry as the LUMO of the other. For such a case, the HOMOs of the two molecules have opposite symmetries and do not interact, and there is no destabilizing closed-shell repulsion between them. Donor and acceptor substituents polarize the HOMO and LUMO of molecules in opposite directions, so there is always an opposite relationship between stabilizing HOMO-LUMO and destabilizing HOMO-HOMO interactions.

This intimate relationship between the IPs of a series of molecules and the LUMO energies, HOMO-LUMO gaps in a molecule, and distortion, charge transfer, electrostatic, and closed-shell repulsion effects accounts for the ability of IP to be such a simple and valuable indicator of reactivity. As the power of analysis increases, we have learned the profound prescience of the Sustmann parabola.

Looking back from the 2021 perspective at Sustmann’s conclusion almost 50 years ago, we are struck by how insightful and influential they were for the organic community, despite the lack of computers of much consequence when he published the paper (at least a billion times less powerful than today’s computers). The real chemical reaction system is complex, and one parameter can never be quantitatively related to reactivity. Nevertheless, chemists appreciate models that give a qualitative guide to reactivity. Nowadays, armed with sophisticated methods of theoretical calculation and analysis, we can provide intimate and complicated details about mechanisms and reactivity. Nevertheless, we acknowledge and admire Sustmann’s insight that the IP of dipolarophiles is a good guide to reactivities of dipolarophiles toward ambiphilic azides.

## Supporting Information Available

Optimized geometries and energies of all computed species (PDF)
