# Supplementary material for: Computational Exploration of Ambiphilic Reactivity of Azides and Sustmann’s Paradigmatic Parabola
Source: J Org Chem. 2021 Mar 26;86(8):5792–804. doi: 10.1021/acs.joc.1c00239 (PMC8154615; doi:10.1021/acs.joc.1c00239)
Supplement: Supplementary file 1 — jo1c00239_si_001.pdf [file jo1c00239_si_001.pdf]

Supporting Information

Computational Exploration of Ambiphilic Reactivity of Azides and Sustmann's Paradigmatic Parabola

Pan-Pan Chen,[a] Pengchen Ma,[a] Xue He,[b] Dennis Svatunek,[a] Fang Liu[b*] and K. N. Houk[a*]

[a]Department of Chemistry and Biochemistry, University of California, Los Angeles, California, 90095-1569, USA

[b]College of Sciences, Nanjing Agricultural University, Nanjing, 210095, China

*Corresponding authors: acialiu@njau.edu.cn; houk@chem.ucla.edu

Table of Contents

1. **Correlation of the calculated and experimental ionization potentials** **S-2**
2. **Plot of predicted ionization potential versus electron affinity** **S-3**
3. **Correlation of Mulliken electronegativity with predicted rate constant** **S-4**
4. **Correlation of predicated ionization potential with Mulliken electronegativity** **S-5**
5. **Distortion/interaction-activation strain analysis on the transition states of cycloadditions of phenyl azide with distortion-activated dipolarophiles** **S-6**
6. **Correlation of predicated ionization potential with both FMO gaps** **S-7**
7. **Energy decomposition analysis of the origins of the different interaction energies** **S-8**
8. **Explorations of the origins of regioselectivity for selected dipolarophiles** **S-9**
9. **Table of energies** **S-9**
10. **References** **S-16**
11. **Cartesian coordinates of the structures (optimization in benzene)** **S-17**
12. **Cartesian coordinates of the structures (optimization in carbon tetrachloride)** **S-22**

1. Correlation of the calculated and experimental ionization potentials

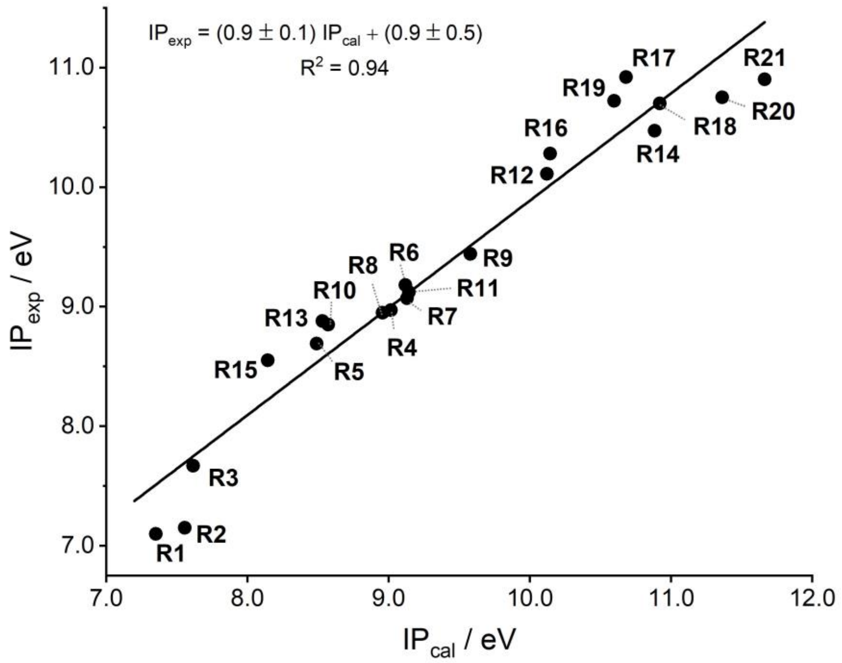

Figure S1. Correlation of the calculated and experimental values of ionization potentials (IPs, eV) for different dipolarophiles. Predicated ionization potential was calculated at HF/6-31G(d) level of theory.

2. Plot of predicted ionization potential versus electron affinity

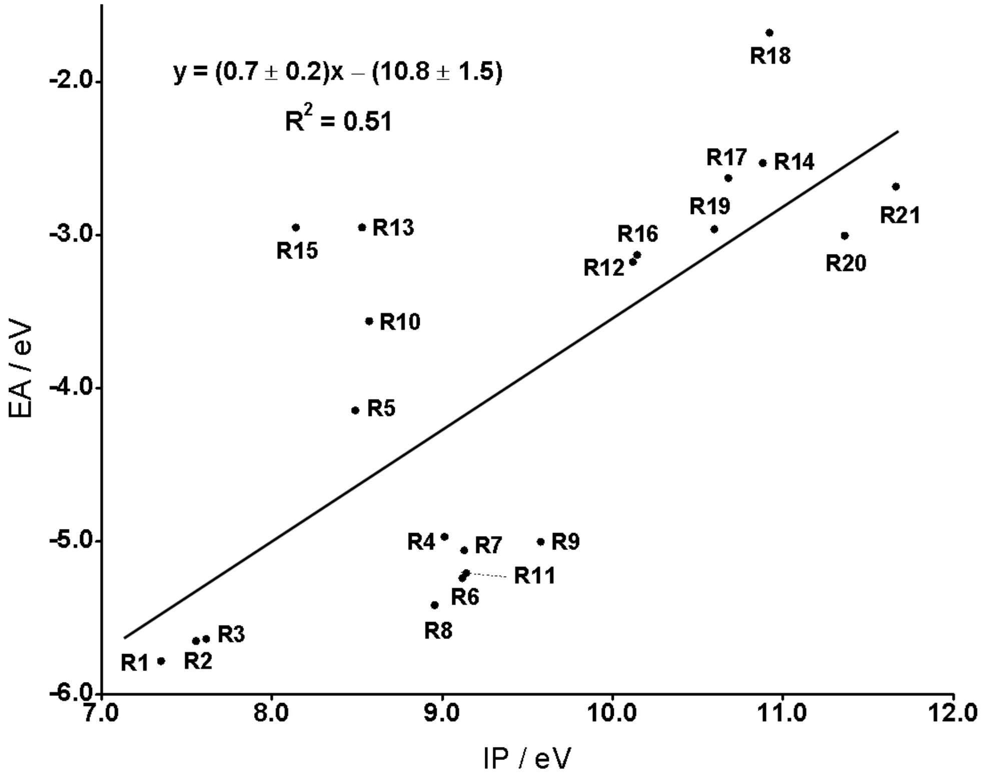

Figure S2. Plot of predicated ionization potentials (IPs, eV) with electron affinities (EAs, eV) for different dipolarophiles.

3. Correlation of Mulliken electronegativity with predicted rate constant

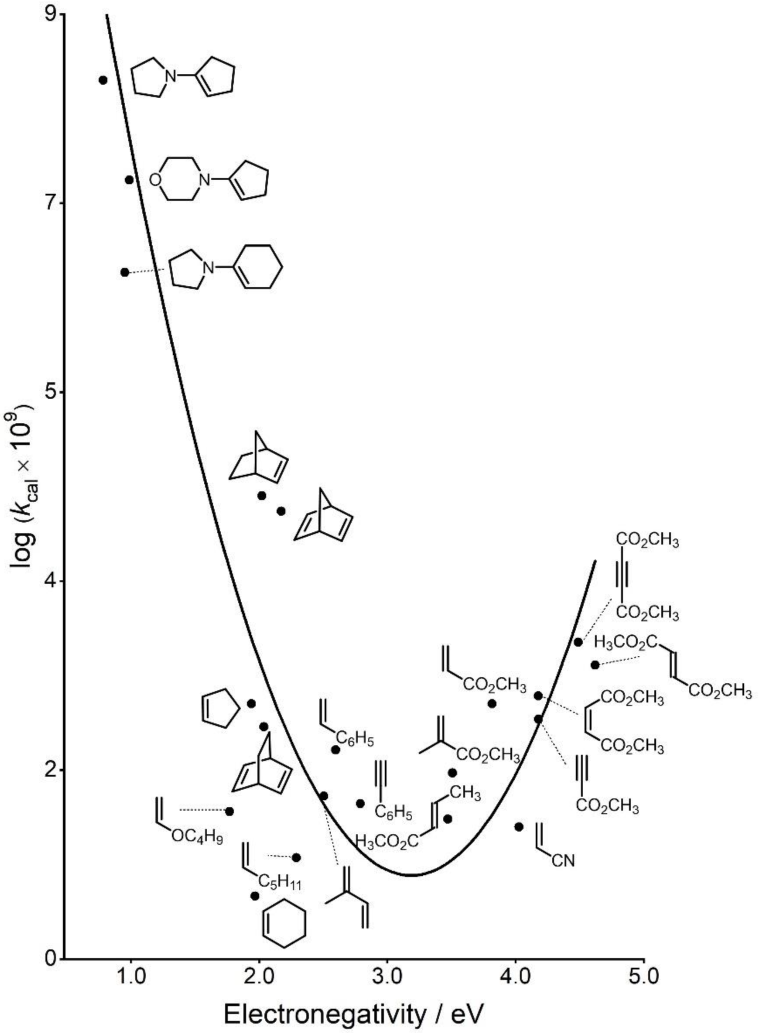

Figure S3. Correlation of Mulliken electronegativities (eV) with predicted rate constants for the cycloadditions between phenyl azide and dipolarophiles. Mulliken electronegativity is the average of the ionization energy (eV) and the electron affinity (eV).

4. Correlation of predicated ionization potential with Mulliken electronegativity

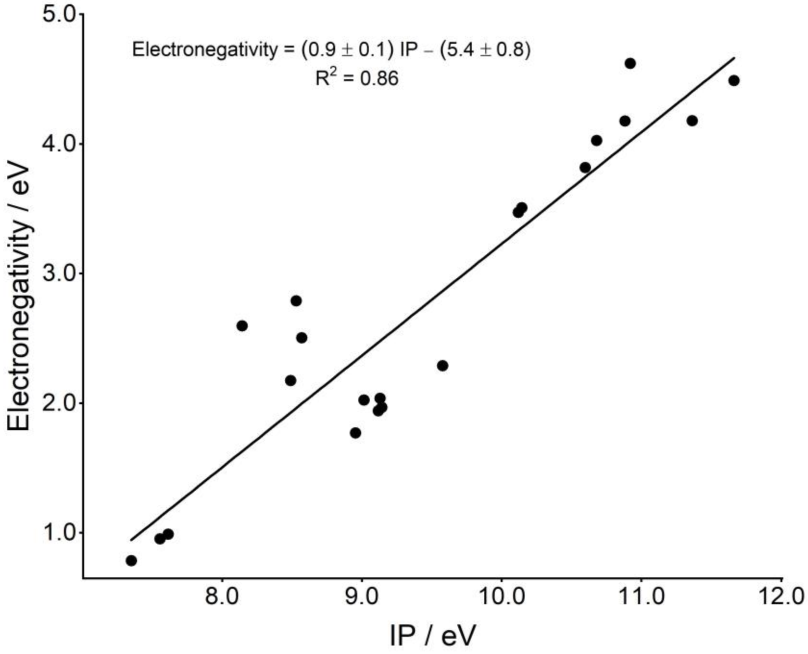

Figure S4. Plot of predicted ionization potentials (IPs, eV) versus Mulliken electronegativities (eV) for different dipolarophiles.

5. Distortion/interaction-activation strain analysis on the transition states of cycloadditions of phenyl azide with distortion-activated dipolarophiles

We performed distortion/interaction-activation strain analysis on the transition states (**TS-cyclooctene**, **TS-cyclooctyne** and **TS-cyclopropene**) of cycloadditions of phenyl azide with *trans*-cyclooctene, cyclooctyne, and cyclopropene, respectively. DFT-optimized transition structures are shown in Figure S5. Each transition state was separated into two fragments (the distorted phenyl azide and dipolarophile), followed by single point energy calculations at the ωB97X-D level of theory with aug-cc-pVTZ basis set in the gas phase. The energy differences between the distorted structures and optimized ground-state structures are the distortion energies of azide ($\Delta E_{\text{dist-azide}}$) and dipolarophile ($\Delta E_{\text{dist-dipolarophile}}$), respectively. The interaction energy (ΔE_{int}) is the difference between the activation energy (ΔE_{act}) and the total distortion energy ($\Delta E_{\text{dist-total}} = \Delta E_{\text{dist-azide}} + \Delta E_{\text{dist-dipolarophile}}$). We use **TS4**, in which the dipolarophile is norbornene (**R4**), as a reference point to discuss the effect of distortion energy on the reactivity.

The results of distortion/interaction-activation strain analyses are shown in Table S1. Comparing **TS-cyclooctene**, **TS-cyclooctyne**, **TS-cyclopropene** and **TS4**, we find that the trend of the cycloaddition reactivity (ΔE_{act}, blue data) is in line with that of the distortion energy ($\Delta E_{\text{dist-total}}$, red data) of the transition state. Compared with **TS4**, **TS-cyclooctene**, **TS-cyclooctyne**, and **TS-cyclopropene** have smaller interaction energies (ΔE_{int}, purple data) but lower activation energies (ΔE_{act}, blue data), indicating that the smaller distortion energies ($\Delta E_{\text{dist-total}}$, red data) of these three transition states make them lower in energy compared to **TS4**, and the corresponding cycloaddition reactions more reactive. In addition, the interaction energies (ΔE_{int}) of **TS-cyclooctene**, **TS-cyclooctyne**, **TS-cyclopropene** are very similar, but the distortion energies ($\Delta E_{\text{dist-total}}$) are significantly different, and the trend of reactivity (ΔE_{act}) is follow up with that of distortion energy. Therefore, the above analysis suggests *trans*-cyclooctene, cyclooctyne, and cyclopropene are all distortion-promoted dipolarophiles, thus, the distortion energy is smaller in the corresponding cycloaddition transition state, which in turn makes the dipolarophile more reactive toward 1.3-dipole.

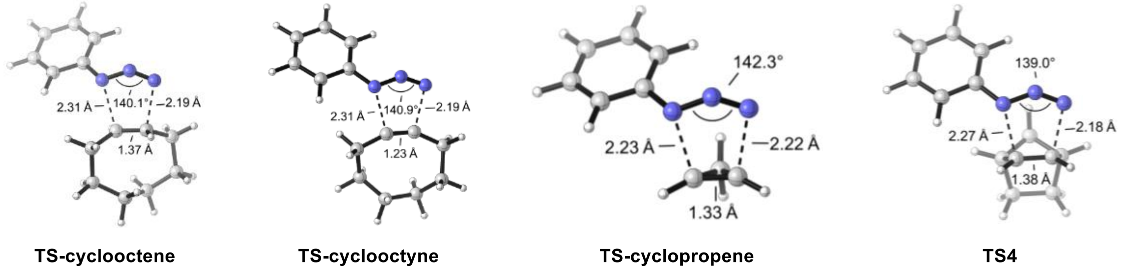

Figure S5. DFT-optimized transition state structures.

Table S1. Results of distortion/interaction-activation strain analyses

Transition state	**TS-cyclooctene**	**TS-cyclooctyne**	**TS-cyclopropene**	**TS4**
dipolarophile	*trans*-cyclooctene	cyclooctyne	cyclopropene	norbornene
ΔE_{act}/kcal/mol	**13.3**	**12.2**	**15.5**	**15.9**
$\Delta E_{\text{dist-azide}}$/kcal/mol	19.2	18.1	17.8	20.9
$\Delta E_{\text{dist-dipolarophile}}$/kcal/mol	2.5	2.6	5.7	4.7
$\Delta E_{\text{dist-total}}$/kcal/mol	**21.7**	**20.7**	**23.5**	**25.6**
ΔE_{int}/kcal/mol	**–8.4**	**–8.5**	**–8.9**	**–9.7**

6. Correlation of predicated ionization potential with both FMO gaps

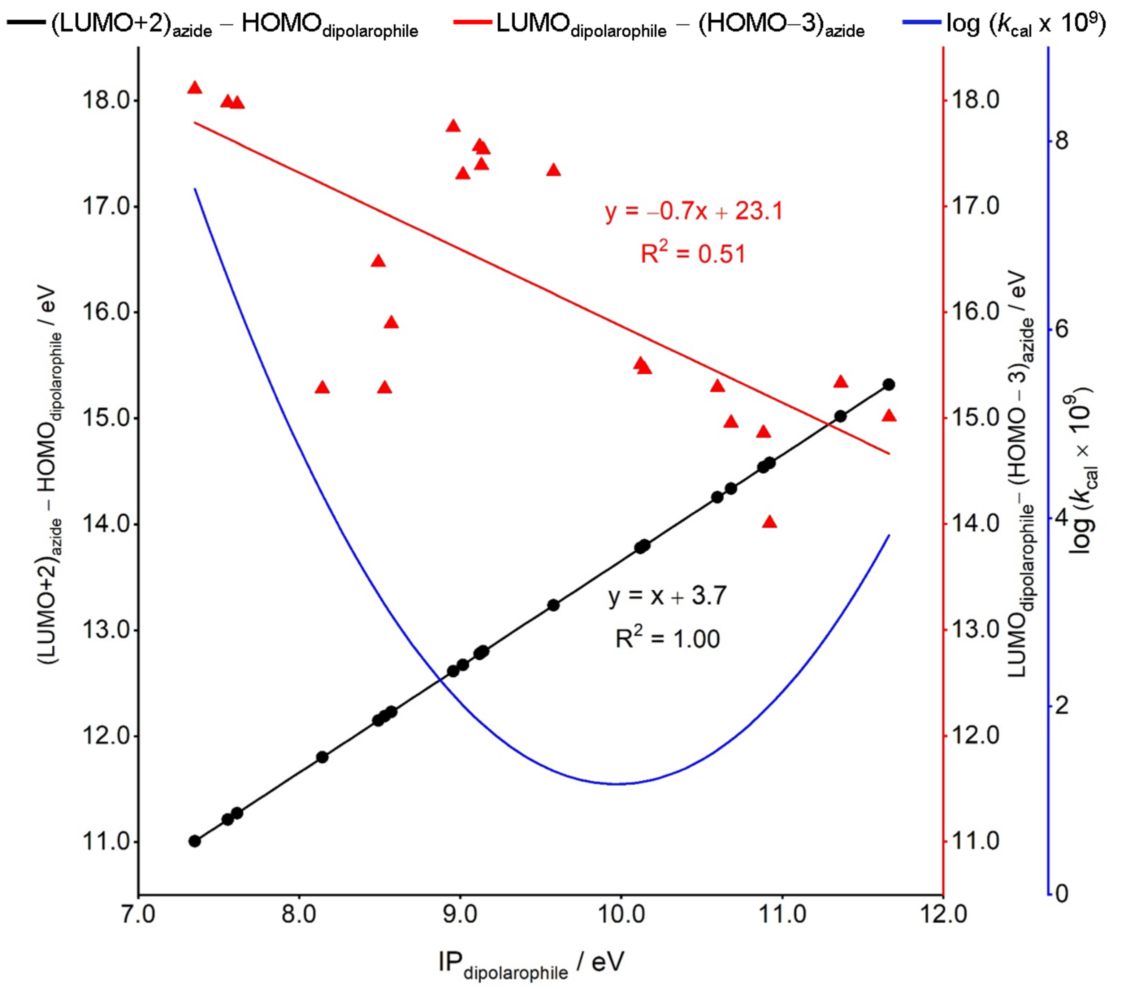

Figure S6 Correlation of predicated ionization potentials (IPs, eV) with both FMO gaps ($HOMO_{dipolarophile}$-$(LUMO+2)_{azide}$ and $LUMO_{dipolarophile}$-$(HOMO{-}3)_{azide}$, eV) for different dipolarophiles.

7. Energy decomposition analysis of the origins of the different interaction energies

Interaction energies are more complex due to the influence of various different contributing factors, therefore, an energy decomposition analysis was performed to determine the origins of the different interaction energies (Figure S7). Symmetrical cases (**R4**, **R11** and **R21**) show higher unfavorable Pauli repulsion at forming bond lengths close to the transition state (Figure S7a). This can be rationalized by the fact that the cycloaddition transition states of these dipolarophiles have larger synchronicity, that is, the two C–N bonds are almost forming simultaneously, and larger overlap of orbitals and consequently stronger Pauli repulsion results. On the other hand, orbital interactions are generally more favorable for symmetrical dipolarophiles than unsymmetrical ones due to greater orbital overlap (Figure S7b). For this reason, **R1**, despite having a much smaller LUMO+2_{azide}-$HOMO_{R1}$ gap, has a reduced ΔE_{OI} at a given forming bond distance, compared to **R4**. The trend of electrostatic interaction is very similar to that of orbital interaction, that is, there are more favorable electrostatic interactions in symmetrical dipolarophiles than asymmetrical ones (Figure S7c). However, these favorable interactions still cannot offset the unfavorable Pauli repulsion, resulting in less interactions for symmetrical cases. For asymmetrical cases (**R1** and **R17**), the asynchronicity of transition states results in less Pauli repulsion, which is the leading cause for a better interaction in case of **R1**. Although there is also less Pauli repulsion for **R17**, orbital interaction is less favorable compared to others due to a bigger FMO gap, leading to less favorable interaction between azide and alkene (Figure 9c).

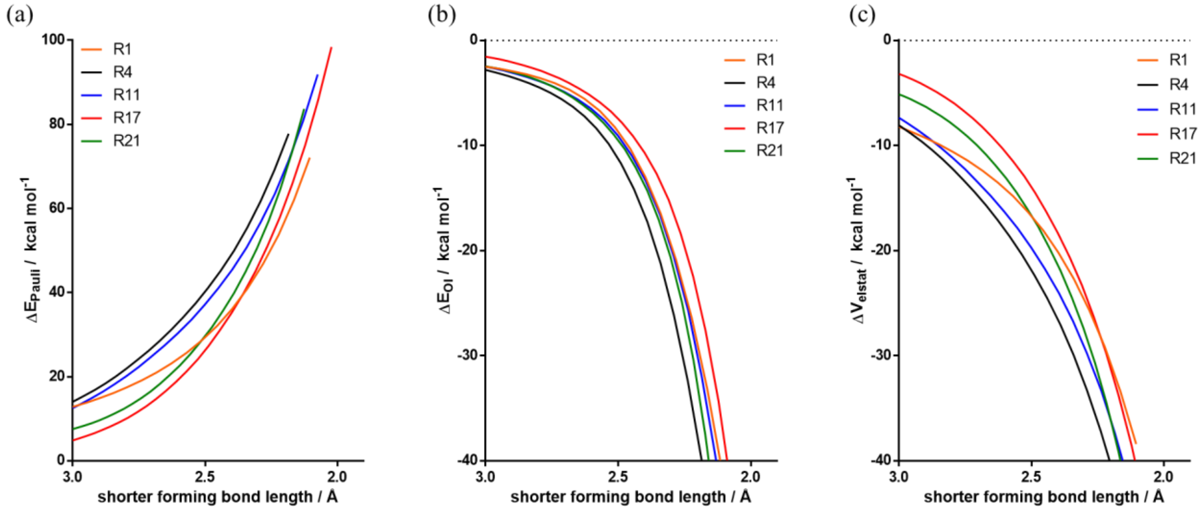

Figure S7. Energy decomposition analysis (EDA) of interaction energies for selected cycloaddition reactions. EDA results were calculated using ADF. (a) Pauli repulsion energies (ΔE_{Pauli}). (b) Orbital interaction energies (ΔE_{OI}). (c) Electrostatic potential (ΔV_{elstat}). Dispersion energies are omitted as they show negligible variation along the reaction coordinate.

8. Explorations of the origins of regioselectivity for selected dipolarophiles

Table S2. The amount of charge transfer in the cycloaddition transition state and orbital coefficients for specific orbitals[a]

Entry	Alkene	Transition state[b]	Charge transfer (e)	LUMO+2 of azide		HOMO of alkene	
				N^1 (%)	N^2 (%)	C^3 (%)	C^4 (%)
1	**R2**	**TS2**	0.31	6.7	53.1	16.4	42.0
		**TS2***	0.19				
2	**R8**	**TS8**	0.20			27.5	50.6
		**TS8***	0.12				
3	**R9**	**TS9**	0.11			40.6	49.1
		**TS9***	0.09				
4	**R15**	**TS15**	0.10			9.9	23.5
		**TS15***	0.06				
5	**R17**	**TS17**	0.01			32.5	35.8
		**TS17***	0.03				

[a]In azide, N^1 is the nitrogen attached to phenyl group, and N^2 is the terminal unsubstituted nitrogen. In alkene, C^3 is the carbon attached to the substituent, and C^4 is the terminal unsubstituted carbon.
[b]The starred transition state is the regioisomeric transition state, which is disfavorable compared to the one without an asterisk.

9. Table of energies

Table S3. Energies in Table 1 and Figure 11. Zero-point vibrational energy (*ZPVE*), thermal correction to enthalpy (*TCH*), thermal correction to Gibbs free energy (*TCG*), energies (*E*), enthalpies (*H*), and Gibbs free energies (*G*) (in Hartree) of the structures calculated at the ωB97X-D/aug-cc-pVTZ-CPCM(Benzene/Carbon tetrachloride)//ωB97X-D/6-31+G(d,p)-CPCM(Benzene/Carbon tetrachloride) level of theory. *Note*: "Pn" (n = 1–21) refers to the cycloaddition product.

Structures	*ZPVE*	*TCH*	*TCG*	*E*	*H*	*G*	Imaginary Frequency
**Phenyl azide**	0.104692	0.112563	0.072801	-395.833651	-395.602804	-395.642566	
**Phenyl azide**[a]	0.104684	0.112558	0.072773	-395.833687	-395.602853	-395.642638	
**R1**[a]	0.228911	0.239257	0.193969	-406.741819	-406.394154	-406.439442	
**TS1**[a]	0.334723	0.352499	0.288227	-802.560202	-801.988055	-802.052327	323.3*i*
**P1**[a]	0.340269	0.356642	0.297059	-802.618391	-802.047443	-802.107026	
**R2**[a]	0.258703	0.269938	0.222297	-446.061774	-445.674172	-445.721813	
**TS2**[a]	0.364538	0.383255	0.316944	-841.876606	-841.264779	-841.331090	365.3*i*
**P2**[a]	0.369326	0.387100	0.323854	-841.932562	-841.321368	-841.384614	
**R3**[a]	0.234509	0.245595	0.198288	-481.948892	-481.567947	-481.615254	
**TS3**[a]	0.340426	0.358853	0.293414	-877.766276	-877.160947	-877.226386	335.2*i*

**P3**[a]	0.345662	0.362857	0.301122	-877.826026	-877.222015	-877.283750	
**R4**[a]	0.154279	0.160396	0.125782	-272.738436	-272.506959	-272.541573	
**TS4**[a]	0.260277	0.273731	0.219984	-668.546334	-668.089186	-668.142933	404.9*i*
**P4**[a]	0.265920	0.278430	0.227444	-668.637185	-668.179425	-668.230411	
**R5**	0.129847	0.135610	0.101749	-271.484584	-271.276794	-271.310655	
**TS5**	0.235708	0.248866	0.195626	-667.291599	-666.858233	-666.911473	407.7*i*
**P5**	0.241763	0.253886	0.203751	-667.389244	-666.955095	-667.005230	
**R6**[a]	0.117696	0.123245	0.090385	-195.332558	-195.157195	-195.190055	
**TS6**[a]	0.224054	0.236556	0.185268	-591.136611	-590.736176	-590.787464	484.8*i*
**P6**[a]	0.229628	0.241269	0.192223	-591.220259	-590.818805	-590.867851	
**R7**	0.184223	0.191418	0.154244	-312.069905	-311.798418	-311.835592	
**TS7**	0.290356	0.304841	0.248970	-707.873131	-707.376239	-707.432110	400.9*i*
**P7**	0.295838	0.309529	0.255349	-707.961437	-707.463845	-707.518025	
**R8**	0.171168	0.181122	0.136093	-311.076155	-310.804302	-310.849331	
**TS8**	0.277817	0.294672	0.232078	-706.881576	-706.384595	-706.447189	421.6*i*
**P8**	0.283100	0.298852	0.240051	-706.957235	-706.460258	-706.519059	
**R9**	0.195490	0.205450	0.161120	-275.173506	-274.895381	-274.939711	
**TS9**	0.301890	0.319023	0.255426	-670.974793	-670.470991	-670.534588	448.6*i*
**P9**	0.307535	0.323794	0.262658	-671.054758	-670.549496	-670.610632	
**R10**	0.114423	0.121165	0.085967	-195.312907	-195.137717	-195.172915	
**TS10**	0.220578	0.234472	0.180190	-591.114920	-590.714668	-590.768950	476.6*i*
**P10**	0.225636	0.238894	0.185729	-591.190388	-590.789250	-590.842415	
**R11**	0.147394	0.153806	0.119446	-234.656154	-234.440847	-234.475207	
**TS11**	0.253600	0.267262	0.212775	-630.454478	-630.014177	-630.068664	478.3*i*
**P11**	0.259192	0.271787	0.220806	-630.537281	-630.095895	-630.146876	
**R12**	0.124498	0.133564	0.091906	-345.809111	-345.568660	-345.610319	
**TS12**	0.230672	0.246964	0.186278	-741.611160	-741.144735	-741.205421	463.2*i*
**P12**	0.235589	0.251124	0.191808	-741.681611	-741.214467	-741.273782	
**R13**	0.110715	0.118004	0.080355	-308.386523	-308.182746	-308.220395	
**TS13**	0.216124	0.230956	0.173416	-704.187062	-703.757856	-703.815397	510.2*i*
**P13**	0.223517	0.236943	0.183027	-704.332926	-703.903401	-703.957317	
**R14**	0.140092	0.151883	0.102191	-534.372544	-534.048739	-534.098431	
**TS14**	0.245938	0.265169	0.196846	-930.176259	-929.627124	-929.695447	463.9*i*
**P14**	0.250949	0.269395	0.202473	-930.247492	-929.697352	-929.764275	
**R15**	0.134641	0.142369	0.102124	-309.643648	-309.417808	-309.458052	
**TS15**	0.240974	0.255674	0.198884	-705.448154	-704.997416	-705.054206	492.7*i*
**P15**	0.246071	0.260202	0.203919	-705.523126	-705.071206	-705.127488	
**R16**	0.124403	0.133389	0.091772	-345.806157	-345.565775	-345.607392	
**TS16**	0.230667	0.246787	0.187197	-741.609228	-741.143479	-741.203069	472.9*i*
**P16**	0.235434	0.250854	0.192802	-741.680682	-741.214131	-741.272183	
**R17**	0.051277	0.056356	0.025397	-170.831181	-170.723156	-170.754115	

**TS17**	0.157917	0.169930	0.119211	-566.633735	-566.299075	-566.349794	489.3*i*
**P17**	0.162816	0.174188	0.124874	-566.702795	-566.367261	-566.416575	
**R18**	0.140111	0.152023	0.102181	-534.379587	-534.056302	-534.106143	
**TS18**	0.246216	0.265348	0.197154	-930.184846	-929.635807	-929.704002	482.2*i*
**P18**	0.250639	0.269259	0.201171	-930.250342	-929.700122	-929.768210	
**R19**	0.096263	0.103797	0.065889	-306.486057	-306.285360	-306.323268	
**TS19**	0.202695	0.217359	0.160085	-702.290838	-701.863621	-701.920896	471.2*i*
**P19**	0.207679	0.221760	0.165449	-702.362072	-701.933770	-701.990081	
**R20**	0.072108	0.079378	0.042193	-305.217571	-305.038155	-305.075340	
**TS20**	0.177930	0.192596	0.135789	-701.020508	-700.614999	-700.671805	510.0*i*
**P20**	0.184655	0.198120	0.143473	-701.173001	-700.762666	-700.817314	
**R21**	0.116254	0.128102	0.077672	-533.104953	-532.801746	-532.852176	
**TS21**	0.221851	0.241236	0.171988	-928.910177	-928.380599	-928.449847	454.6*i*
**P21**	0.227830	0.245953	0.180019	-929.056023	-928.527979	-928.593913	
**TS2•**[a,b]	0.364552	0.383143	0.317931	-841.856964	-841.245618	-841.310830	426.5*i*
**TS8•**[b]	0.277549	0.294442	0.231008	-706.872306	-706.375204	-706.438638	490.7*i*
**TS9•**[b]	0.301937	0.319004	0.255301	-670.972745	-670.468818	-670.532521	436.3*i*
**TS15•**[b]	0.241033	0.255756	0.198378	-705.444627	-704.993373	-705.050751	494.3*i*
**TS17•**[b]	0.157927	0.169859	0.119722	-566.631609	-566.297563	-566.347701	485.3*i*

[a]For these species, we chose benzene as the solvent according to experimental reports.[1]
[b]The starred transition states are the regioisomeric transition states.

Table S4. Energies in Table 1 and Figure 4. Zero-point vibrational energy (*ZPVE*), thermal correction to enthalpy (*TCH*), thermal correction to Gibbs free energy (*TCG*), energies (*E*), enthalpies (*H*), and Gibbs free energies (*G*) (in Hartree) of the structures calculated at the B2PLYP-D3/aug-cc-pVTZ-CPCM(Benzene/Carbon tetrachloride)//ωB97X-D/6-31+G(d,p)-CPCM(Benzene/Carbon tetrachloride) level of theory. *Note*: "Pn" (n = 1–21) refers to the cycloaddition product.

**Structures**	***ZPVE***	***TCH***	***TCG***	***E***	***H***	***G***	**Imaginary Frequency**
**Phenyl azide**	0.104692	0.112563	0.072801	-395.685312	-395.572749	-395.612511	
**Phenyl azide**[a]	0.104684	0.112558	0.072773	-395.685350	-395.572792	-395.612577	
**R1**[a]	0.228911	0.239257	0.193969	-406.514692	-406.275435	-406.320723	
**TS1**[a]	0.334723	0.352499	0.288227	-802.192308	-801.839809	-801.904081	323.3*i*
**P1**[a]	0.340269	0.356642	0.297059	-802.232354	-801.875712	-801.935295	
**R2**[a]	0.258703	0.269938	0.222297	-445.810480	-445.540542	-445.588183	
**TS2**[a]	0.364538	0.383255	0.316944	-841.484495	-841.101240	-841.167551	365.3*i*
**P2**[a]	0.369326	0.387100	0.323854	-841.521933	-841.134833	-841.198079	
**R3**[a]	0.234509	0.245595	0.198288	-481.704847	-481.459252	-481.506559	
**TS3**[a]	0.340426	0.358853	0.293414	-877.381236	-877.022383	-877.087822	335.2*i*
**P3**[a]	0.345662	0.362857	0.301122	-877.422836	-877.059979	-877.121714	
**R4**[a]	0.154279	0.160396	0.125782	-272.584425	-272.424029	-272.458643	
**TS4**[a]	0.260277	0.273731	0.219984	-668.253451	-667.979720	-668.033467	404.9*i*

**P4**[a]	0.265920	0.278430	0.227444	-668.323286	-668.044856	-668.095842	
**R5**	0.129847	0.135610	0.101749	-271.342746	-271.207136	-271.240997	
**TS5**	0.235708	0.248866	0.195626	-667.011122	-666.762256	-666.815496	407.7*i*
**P5**	0.241763	0.253886	0.203751	-667.087771	-666.833885	-666.884020	
**R6**[a]	0.117696	0.123245	0.090385	-195.219033	-195.095788	-195.128648	
**TS6**[a]	0.224054	0.236556	0.185268	-590.884366	-590.647810	-590.699098	484.8*i*
**P6**[a]	0.229628	0.241269	0.192223	-590.947527	-590.706258	-590.755304	
**R7**	0.184223	0.191418	0.15424	-311.891703	-311.700285	-311.737459	
**TS7**	0.290356	0.304841	0.248970	-707.556361	-707.251520	-707.307391	400.9*i*
**P7**	0.295838	0.309529	0.255349	-707.622852	-707.313323	-707.367503	
**R8**	0.171168	0.181122	0.136093	-310.912169	-310.731047	-310.776076	
**TS8**	0.277817	0.294672	0.232078	-706.576449	-706.281777	-706.344371	421.6*i*
**P8**	0.283100	0.298852	0.240051	-706.633217	-706.334365	-706.393166	
**R9**	0.195490	0.205450	0.161120	-275.002234	-274.796784	-274.841114	
**TS9**	0.301890	0.319023	0.255426	-670.663893	-670.344870	-670.408467	448.6*i*
**P9**	0.307535	0.323794	0.262658	-670.723027	-670.399233	-670.460369	
**R10**	0.114423	0.121165	0.085967	-195.204514	-195.083349	-195.118547	
**TS10**	0.220578	0.234472	0.180190	-590.867375	-590.632903	-590.687185	476.6*i*
**P10**	0.225636	0.238894	0.185729	-590.921701	-590.682807	-590.735972	
**R11**	0.147394	0.153806	0.119446	-234.517983	-234.440847	-234.475207	
**TS11**	0.253600	0.267262	0.212775	-630.164338	-630.014177	-630.068664	478.3*i*
**P11**	0.259192	0.271787	0.220806	-630.239894	-630.095895	-630.146876	
**R12**	0.124498	0.133564	0.091906	-345.662890	-345.568660	-345.610319	
**TS12**	0.230672	0.246964	0.186278	-741.325333	-741.144735	-741.205421	463.2*i*
**P12**	0.235589	0.251124	0.191808	-741.376493	-741.214467	-741.273782	
**R13**	0.110715	0.118004	0.080355	-308.249363	-308.182746	-308.220395	
**TS13**	0.216124	0.230956	0.173416	-703.910844	-703.757856	-703.815397	510.2*i*
**P13**	0.223517	0.236943	0.183027	-704.035609	-703.903401	-703.957317	
**R14**	0.140092	0.151883	0.102191	-534.176566	-534.048739	-534.098431	
**TS14**	0.245938	0.265169	0.196846	-929.841783	-929.627124	-929.695447	463.9*i*
**P14**	0.250949	0.269395	0.202473	-929.892919	-929.697352	-929.764275	
**R15**	0.134641	0.142369	0.102124	-309.493877	-309.417808	-309.458052	
**TS15**	0.240974	0.255674	0.198884	-705.160084	-704.997416	-705.054206	492.7*i*
**P15**	0.246071	0.260202	0.203919	-705.213558	-705.071206	-705.127488	
**R16**	0.124403	0.133389	0.091772	-345.657003	-345.565775	-345.607392	
**TS16**	0.230667	0.246787	0.187197	-741.324990	-741.143479	-741.203069	472.9*i*
**P16**	0.235434	0.250854	0.192802	-741.375974	-741.214131	-741.272183	
**R17**	0.051277	0.056356	0.025397	-170.763621	-170.723156	-170.754115	
**TS17**	0.157917	0.169930	0.119211	-566.425316	-566.299075	-566.349794	489.3*i*
**P17**	0.162816	0.174188	0.124874	-566.475598	-566.367261	-566.416575	
**R18**	0.140111	0.152023	0.102181	-534.183664	-534.056302	-534.106143	

**TS18**	0.246216	0.265348	0.197154	-929.849880	-929.635807	-929.704002	482.2*i*
**P18**	0.250639	0.269259	0.201171	-929.895503	-929.700122	-929.768210	
**R19**	0.096263	0.103797	0.065889	-306.364778	-306.285360	-306.323268	
**TS19**	0.202695	0.217359	0.160085	-702.018477	-701.863621	-701.920896	471.2*i*
**P19**	0.207679	0.221760	0.165449	-702.081291	-701.933770	-701.990081	
**R20**	0.072108	0.079378	0.042193	-305.109326	-305.038155	-305.075340	
**TS20**	0.177930	0.192596	0.135789	-700.773009	-700.614999	-700.671805	510.0*i*
**P20**	0.184655	0.198120	0.143473	-700.899684	-700.762666	-700.817314	
**R21**	0.116254	0.128102	0.077672	-532.922405	-532.801746	-532.852176	
**TS21**	0.221851	0.241236	0.171988	-928.588422	-928.380599	-928.449847	454.6*i*
**P21**	0.227830	0.245953	0.180019	-928.713047	-928.527979	-928.593913	
***trans*-cyclooctene**[a]	0.205370	0.213827	0.174191	-312.6195257	-312.967112	-313.006748	
**cyclooctyne**[a]	0.181534	0.190216	0.149819	-311.3914046	-311.737725	-311.778121	
**cyclopropene**[a]	0.056499	0.060760	0.032556	-116.394217	-116.525279	-116.553483	
**TS-cyclooctene**[a]	0.310980	0.327129	0.266790	-707.7503743	-708.553039	-708.613378	382.1*i*
**TS-cyclooctyne**[a]	0.286946	0.303367	0.242321	-706.5234397	-707.324760	-707.385805	391.3*i*
**TS-cyclopropene**[a]	0.162978	0.174081	0.126058	-511.521783	-512.105392	-512.153415	475.6*i*
**1-methoxycyclopentene**	0.151334	0.159220	0.120505	-309.302603	-309.621593	-309.660308	
**maleic anhydride**	0.056504	0.062594	0.027454	-378.759445	-379.125198	-379.160338	
**N-phenylmaleimide**	0.150613	0.161482	0.113960	-589.527234	-590.156687	-590.204209	
**TS-methoxycyclopentene**	0.256938	0.272212	0.213960	-704.423831	-705.201560	-705.259811	416.0*i*
**TS-maleic anhydride**	0.162593	0.175906	0.122032	-773.880472	-774.704242	-774.758117	466.5*i*
**TS-N-phenylmaleimide**	0.256626	0.274907	0.208493	-984.649949	-985.736624	-985.803038	475.3*i*

[a]For these species, we chose benzene as the solvent according to experimental reports.[1]

Table S5. Calculated ionization potentials and electron affinities for all the dipolarophiles shown in Figure 4.

Dipolarophile	Ionization potential (eV)	electron affinity (eV)
**R1**[a]	7.35	-5.78
**R2**[a]	7.56	-5.65
**R3**[a]	7.62	-5.64
**R4**[a]	9.02	-4.97
**R5**[a]	8.49	-4.15
**R6**[a]	9.12	-5.24
**R7**[a]	9.13	-5.06
**R8**[a]	8.96	-5.42
**R9**[a]	9.58	-5.00
**R10**[a]	8.57	-3.56
**R11**[a]	9.14	-5.21
**R12**[a]	10.12	-3.18
**R13**[a]	8.53	-2.95
**R14**[a]	10.88	-2.53
**R15**[a]	8.14	-2.95
**R16**[a]	10.14	-3.13
**R17**[a]	10.68	-2.63
**R18**[a]	10.92	-1.68
**R19**[a]	10.60	-2.96

**R20**[b]	11.36	-3.00
**R21**[a]	11.66	-2.69
**cyclooctyne**[a]	9.37	-4.87
***trans*-cyclooctene**[a]	7.57	-4.65
**cyclopropene**[a]	9.60	-4.93
**propyne**[b]	10.22	-6.03
**1-methoxycyclopentene**[a]	8.51	-5.79
**maleic anhydride**[a]	12.10	-0.75
**N-phenylmaleimide**[c]	11.49	-1.27

[a]Ionization potential is the minus of HOMO energy of the dipolarophile, and electron affinity is the minus of LOMO energy of the dipolarophile. The selected molecular orbitals of the dipolarophile are the ones that are involved in the bond formation.

[b]Ionization potential is the minus of HOMO-1 energy of the dipolarophile, and electron affinity is the minus of LOMO energy of the dipolarophile. The selected molecular orbitals of the dipolarophile are the ones that are involved in the bond formation.

[c]Ionization potential is the minus of HOMO-2 energy of the dipolarophile, and electron affinity is the minus of LOMO energy of the dipolarophile. The selected molecular orbitals of the dipolarophile are the ones that are involved in the bond formation.

Table S6. The data were used to construct Figure 4.

Dipolarophile	Ionization potential (eV)	log ($k_{cal} \times 10^9$)[a]
**R1**[b]	7.35	8.37
**R2**[b]	7.56	6.54
**R3**[b]	7.62	7.42
**R4**[b]	9.02	4.41
**R5**[c]	8.49	4.27
**R6**[b]	9.12	2.43
**R7**[c]	9.13	2.21
**R8**[c]	8.96	1.41
**R9**[c]	9.58	0.97
**R10**[c]	8.57	1.55
**R11**[c]	9.14	0.60
**R12**[c]	10.12	1.33
**R13**[c]	8.53	1.48
**R14**[c]	10.88	2.51
**R15**[c]	8.14	1.99
**R16**[c]	10.14	1.77
**R17**[c]	10.68	1.26
**R18**[c]	10.92	2.80
**R19**[c]	10.60	2.43
**R20**[c]	11.36	2.29
**R21**[c]	11.66	3.02
**cyclooctyne**[d]	9.37	7.06
***trans*-cyclooctene**[d]	7.57	6.55
**cyclopropene**[d]	9.60	4.63
**propyne**[e]	10.22	8.08[e]
**1-methoxycyclopentene**[b,f]	8.51	2.70
**maleic anhydride**[b]	12.10	2.41
**N-phenylmaleimide**[b]	11.49	3.14

[a]The reaction rate constants are calculated at the B2PLYP-D3/aug-cc-pVTZ-CPCM(solvent)//ωB97X-D/6-31+G(d,p)-CPCM(solvent) level of theory.

[b]In the experimental study, the reaction solvent was benzene for these dipolarophiles. In our computational study, we choose the same solvent as the experiment for calculation.[1]

[c]In the experimental study, the reaction solvent was carbon tetrachloride for these dipolarophiles. In our computational study, we choose the same solvent as the experiment for calculation. [1]
[d]Since experimental data show that the solvent effects are marginal,[1] we choose benzene as the solvent to calculate the cycloaddition reactions in which these three species participate in as dipolarophiles.
[e]For this species, the reaction rate constant corresponds to the rate constant of Cu-catalyzed Click reaction of methyl azide with propyne, and we get it from previous study.[2]
[f]The exact dipolarophile used in the experiment is 1-ethoxycyclopentene.[1] In the calculation, 1-methoxycyclopentene is substituted for 1-ethoxycyclopentene in order to simplify the calculation model.

Table S7. The data were used to construct Figure 5.

Dipolarophile	ΔE_{dist} (kcal/mol)	ΔE_{int} (kcal/mol)	log ($k_{exp} \times 10^9$)
**R1**	23.89	-14.35	7.06
**R2**	28.09	-16.12	6.00
**R3**	25.22	-15.16	5.41
**R4**	25.58	-9.68	4.40
**R5**	26.11	-9.77	4.29
**R6**	28.91	-10.64	2.38
**R7**	29.45	-10.62	1.95
**R8**	31.40	-14.06	1.60
**R9**	30.23	-9.88	1.38
**R10**	30.45	-10.87	1.18
**R11**	33.09	-11.23	0.52
**R12**	29.80	-10.11	1.43
**R13**	30.46	-10.43	1.46
**R14**	29.61	-11.49	1.53
**R15**	30.51	-12.52	1.60
**R16**	31.24	-12.61	1.86
**R17**	28.13	-8.50	2.03
**R18**	30.44	-12.92	2.92
**R19**	27.15	-9.22	2.99
**R20**	29.35	-10.89	3.02
**R21**	29.71	-12.07	3.40

Table S8. The data were used to construct Figure 6.

Dipolarophile	Ionization potential (eV)	ΔE_{FMO} (eV)
**R1**	7.35	11.01
**R2**	7.56	11.21
**R3**	7.62	11.27
**R4**	9.02	12.67
**R5**	8.49	12.15
**R6**	9.12	12.78
**R7**	9.13	12.79
**R8**	8.96	12.61
**R9**	9.58	13.24
**R10**	8.57	12.23
**R11**	9.14	12.80
**R12**	10.12	13.78
**R13**	8.53	12.19
**R14**	10.88	14.54
**R15**	8.14	11.80
**R16**	10.14	13.80
**R17**	10.68	14.34
**R18**	10.92	14.01
**R19**	10.60	14.25
**R20**	11.36	15.02
**R21**	11.66	15.01

10. References

(1) Huisgen, R.; Szeimie, G.; Möbius, L. 1.3-Dipolare Cycloadditionen, XXXII. Kinetik der Additionen organischer Azide an CC-Mehrfachbindungen. *Chem. Ber*. **1967**, *100*, 2494–2507.

(2) Himo, F.; Lovell, T.; Hilgraf, R.; Rostovtsev, V. V.; Noodleman, L.; Sharpless, K. B.; Fokin, V. V. Copper(I)-Catalyzed Synthesis of Azoles. DFT Study Predicts Unprecedented Reactivity and Intermediates. *J. Am. Chem. Soc*. **2005**, *127*, 210–216.

11. Cartesian coordinates of the structures (optimization in benzene)

Phenyl azide

C	0.13805500	0.99745000	-0.00001600
C	1.46674200	1.41607600	0.00001400
C	2.50348400	0.48548400	-0.00003200
C	2.20459600	-0.87731800	0.00002900
C	0.88286600	-1.30864200	0.00006400
C	-0.15040500	-0.36953000	-0.00003100
H	-0.66018200	1.73396500	0.00000700
H	1.68785500	2.47859900	0.00003700
H	3.53586900	0.81878800	-0.00006100
H	3.00466700	-1.61056100	0.00005300
H	0.63759900	-2.36531000	0.00012800
N	-1.47019500	-0.89944000	-0.00008900
N	-2.40274800	-0.09264700	-0.00006300
N	-3.33817800	0.54685900	0.00010400

R1

C	-1.44302000	1.18245800	0.12302900
C	-2.87017300	0.74459100	-0.19693100
C	-2.88625200	-0.71731800	0.27019100
C	-1.46579300	-1.22358600	-0.03213700
N	-0.66944400	-0.01282200	-0.18970100
H	-1.35235700	1.46572200	1.18545100
H	-3.62074900	1.36118000	0.30327500
H	-3.65579100	-1.31471000	-0.22395400
H	-1.42035600	-1.82591100	-0.94917200
C	1.52959300	1.19611100	-0.13038000
C	1.48020700	-1.16950900	-0.03777500
C	2.93267000	0.70159000	0.26848900
H	1.51387200	1.61354700	-1.14680900
C	2.94497600	-0.80457800	-0.07274600
H	1.12293200	-2.19269100	-0.07090400
H	3.06169400	0.82679600	1.34884700
H	3.55243800	-1.37806900	0.63670300
C	0.69780600	-0.06889800	-0.08759000
H	-1.11372600	2.03120400	-0.48309400
H	-3.04017200	0.80387100	-1.27723600
H	-3.07430200	-0.75804200	1.34808700
H	-1.07380700	-1.84492700	0.78666100
H	1.15559400	1.97645200	0.54057100
H	3.73195800	1.25996400	-0.22649000
H	3.37878500	-0.97979400	-1.06893600

TS1

C	-1.26704200	1.92333500	1.05240400
C	-1.33960400	3.29379900	0.38643500
C	-1.22661000	2.94497100	-1.10224500
C	-2.01507000	1.63593800	-1.23086700
N	-1.97372300	1.05590200	0.11458600
H	-1.73175500	1.89325700	2.04116000
H	-2.30441200	3.76648200	0.59955200
H	-0.17764800	2.77039000	-1.36175500
H	-1.56069900	0.94735800	-1.95169800
C	-1.92050800	-0.89470600	1.67462700
C	-2.44372900	-1.21805000	-0.63783000
C	-1.88763800	-2.39857400	1.35820200
H	-1.03810700	-0.54546700	2.21386200
C	-2.70814700	-2.53333300	0.06168200
H	-2.95855500	-0.94852200	-1.55236600
H	-2.27438100	-3.00947000	2.17745700
H	-3.77849700	-2.64546700	0.28804400
C	-2.07669700	-0.26594100	0.31031900
H	-0.22420900	1.57840300	1.13917200
H	-0.54702900	3.96156100	0.73019500
H	-1.61313800	3.72711400	-1.75883500
H	-3.05588000	1.80570800	-1.53173700
H	-2.80580300	-0.63565200	2.27355500
H	-0.85209000	-2.69839000	1.16761000
H	-2.40935000	-3.39923500	-0.53715500
C	2.44377100	0.18267700	1.13137900
C	3.79127500	0.44703700	1.35307500
C	4.74140000	0.15034900	0.37577400
C	4.32157900	-0.41545800	-0.82784900
C	2.97512600	-0.68076600	-1.06039800
C	2.01863400	-0.38432300	-0.07776600
H	1.70496500	0.40266300	1.89627600
H	4.10049500	0.88410800	2.29789600
H	5.79257700	0.35610000	0.54942700
H	5.04893700	-0.65139700	-1.59915200
H	2.65922300	-1.11635600	-2.00313900
N	0.63413700	-0.59690200	-0.21434000
N	0.27664100	-1.25517100	-1.24941200
N	-0.71063800	-1.65595900	-1.74798100

P1

C	0.23766300	1.75864500	1.38827000
C	0.75303000	3.12772900	0.95611700
C	0.87445300	2.96246100	-0.56226500
C	-0.34141000	2.10040100	-0.92672600
N	-0.71277100	1.41632500	0.32377700
H	-0.26569200	1.78342200	2.35978500
H	0.01627400	3.90076500	1.20076900
H	1.79771200	2.42201900	-0.79814000
H	-0.10194000	1.39224500	-1.72853600
C	-1.67849600	-0.48231800	1.58889600
C	-2.45635800	-0.06323600	-0.69955100
C	-2.68674900	-1.56719100	1.20998800
H	-0.84794700	-0.83395000	2.20483700
C	-3.48824100	-0.90607500	0.07866900
H	-2.86804800	0.90053500	-1.00653000
H	-3.31489900	-1.87120600	2.05114700
H	-4.24677500	-0.23998500	0.50280900
C	-1.22381800	0.08474300	0.23619100
H	1.07430100	1.04741600	1.45653700
H	1.69871800	3.38849200	1.43721000
H	0.89045700	3.91186700	-1.10259400
H	-1.17876700	2.71560700	-1.27889000
H	-2.18195600	0.32154800	2.13737900
H	-2.16445400	-2.45668400	0.84077400
H	-3.99665000	-1.61748400	-0.57644300
C	1.52468900	-1.66099800	0.88559400
C	2.89039500	-1.75719500	1.13600500
C	3.80963700	-1.24765700	0.21993600
C	3.35386900	-0.64561700	-0.95168100
C	1.98834400	-0.52528800	-1.19475600
C	1.06801300	-1.02306800	-0.26991400
H	0.80941900	-2.08895200	1.57900900
H	3.23639600	-2.24737600	2.04043400
H	4.87444800	-1.32948700	0.41265300
H	4.06263500	-0.25727100	-1.67624400
H	1.63126200	-0.04622900	-2.10009700
N	-0.32851900	-0.88714900	-0.48323700
N	-0.80089600	-1.16387600	-1.72555900
N	-1.96847400	-0.75491500	-1.90419700

R2

C	1.77662900	1.20791400	-0.11930400
C	3.23333100	0.73767500	0.07662900
C	3.17375200	-0.79282200	-0.05631800
C	1.74274700	-1.11822700	0.35703300
N	0.98730100	-0.01401300	-0.22908700
H	1.64298200	1.81897900	-1.01924700
H	3.91415900	1.19085600	-0.64758100
H	3.91594500	-1.30275800	0.56277200
H	1.64911900	-1.13772800	1.45782400
C	-0.40036400	0.05436800	-0.10838700
C	-1.08189300	1.21945900	-0.06361500
C	-1.11289000	-1.28298900	-0.08800400
C	-2.58594800	1.30896600	0.01774100
H	-0.54610200	2.16229700	-0.11328100
C	-2.61360500	-1.16179300	-0.35903200
H	-0.65908000	-1.94130700	-0.83730100
C	-3.22271000	-0.01417400	0.44241100
H	-2.86551100	2.10161800	0.72242400
H	-3.10556400	-2.11136300	-0.12306700
H	-3.03948300	-0.18341700	1.51203000

H	1.44473400	1.81492400	0.73887800
H	3.58422800	1.01667500	1.07453100
H	3.32855600	-1.09615100	-1.09662100
H	1.40373800	-2.08246600	-0.02699900
H	-3.00014500	1.61467800	-0.95540900
H	-4.30848100	0.02267600	0.30249000
H	-2.77502500	-0.97490900	-1.42884900
H	-0.94946900	-1.76477200	0.88609900

TS2

C	-1.49042700	2.06319500	-1.22700100
C	-0.55267400	3.25191200	-0.98413300
C	-0.68907400	3.52840000	0.51521200
C	-0.84311900	2.12769700	1.09491300
N	-1.63137000	1.42194000	0.08663400
H	-1.06798600	1.35822300	-1.94986400
H	0.47672500	2.95996800	-1.21365500
H	-1.58687600	4.12126700	0.72050300
H	-1.33633400	2.12097600	2.06855700
C	-1.96988500	0.12325900	0.21323000
C	-2.38026900	-0.63637400	-0.89073800
C	-1.91939400	-0.46664800	1.60863000
C	-3.01121800	-1.99647200	-0.70536000
H	-2.71385400	-0.08725100	-1.76517700
C	-2.68068300	-1.78838000	1.75189000
H	-0.86834300	-0.60284100	1.87959400
C	-2.47806300	-2.69580200	0.54164600
H	-4.10365100	-1.89663600	-0.62648500
H	-3.75343500	-1.58113200	1.85813700
H	-2.99201700	-3.65085800	0.69087800
H	-2.47542100	2.37652300	-1.59282800
H	-0.80821500	4.11150800	-1.60700200
H	0.17189000	4.05328200	0.93417700
H	0.13246300	1.62308800	1.18781300
H	-2.81523200	-2.59763800	-1.59974300
H	-1.41029100	-2.91522600	0.41217800
H	-2.36121100	-2.28222800	2.67489100
H	-2.33421800	0.26407700	2.31035100
C	2.13848400	-0.50053800	-0.09090200
C	2.60790400	-0.06889400	1.15782800
C	3.97126600	-0.04309900	1.43275000
C	4.89573600	-0.44809100	0.46989800
C	4.43299000	-0.87962400	-0.77313800
C	3.07096100	-0.90615300	-1.05842900
H	1.88844100	0.23602800	1.91210000
H	4.31242100	0.29246900	2.40768800
H	5.95927400	-0.42841400	0.68449200
H	5.13981200	-1.19727400	-1.53427200
H	2.72343300	-1.23899000	-2.03128000
N	0.74382900	-0.47344500	-0.27705300
N	0.33370300	-1.01621300	-1.36400900
N	-0.70224600	-1.22160300	-1.89683400

P2

C	-1.58742800	1.61294000	-1.20642000
C	-1.81392900	3.08419100	-0.83834200
C	-2.47368000	3.00932200	0.54178900
C	-1.74149700	1.84028500	1.20042300
N	-1.53145300	0.92921900	0.08336500
H	-0.65722200	1.48088200	-1.77450100
H	-0.85017800	3.59948400	-0.76011900
H	-3.53809900	2.77056000	0.44209400
H	-2.33895500	1.37578400	1.99089700
C	-0.92724800	-0.37108600	0.16772900
C	-1.73666400	-1.42509800	-0.64761200
C	-0.72198600	-0.81427400	1.61946300
C	-1.92514600	-2.75469400	0.09048300
H	-2.70884100	-1.01039900	-0.92635900
C	-0.05887700	-2.19321600	1.73385400
H	-0.12287300	-0.07313900	2.15649300
C	-0.61501400	-3.22424900	0.72323700
H	-2.69703200	-2.62450400	0.85801200
H	-0.20373400	-2.54791100	2.75904300
H	-0.76589100	-4.19171700	1.21069100
H	-2.40615000	1.21937500	-1.82718500
H	-2.41812100	3.61325700	-1.57874400
H	-2.38126100	3.93483000	1.11536700
H	-0.80776100	2.19931000	1.66853500
H	-2.30235500	-3.49697100	-0.61949000
H	0.11489300	-3.39415100	-0.07611500
H	1.02135000	-2.08610700	1.59356200
H	-1.70568300	-0.82800400	2.09963200
C	1.60760600	0.05223200	-0.30548900
C	1.73320100	1.22563800	0.44354500
C	2.99684200	1.72104700	0.75658000
C	4.14431600	1.07191900	0.30874200
C	4.01585800	-0.09003900	-0.45265800
C	2.76063000	-0.60838800	-0.75020000
H	0.84870000	1.75947100	0.76426100
H	3.07818900	2.63154400	1.34218000
H	5.12671700	1.46556600	0.54781300
H	4.90114100	-0.60874200	-0.80726200
H	2.66259500	-1.52233500	-1.32464500
N	0.34378400	-0.47919500	-0.60320100
N	0.17691900	-1.14816300	-1.76941400
N	-0.95944000	-1.65689900	-1.87716400

R3

C	-1.09171800	1.29771400	0.11835300
C	-2.58573900	1.05675200	0.32537400
C	-2.47010700	-1.22347000	-0.28156400
C	-1.13741100	-1.12280900	0.47025500
N	-0.39490500	0.02684100	-0.00998700
H	-0.92703300	1.88456200	-0.79379100
H	-2.80111100	0.79279200	1.37119200
H	-3.17073700	-1.83927900	0.29950000
H	-1.30573500	-1.06894400	1.55811200
C	0.98552200	0.06175500	-0.00667400
C	1.76930600	1.15962600	-0.06890200
C	1.81323100	-1.20687200	-0.06858200
C	3.22429500	0.79564900	-0.23120300
H	1.41480500	2.18327700	-0.10179500
C	3.25112600	-0.69879500	0.15314400
H	1.68804800	-1.68328000	-1.05072300
H	1.52923000	-1.94764000	0.68529500
H	3.55816100	0.94463800	-1.26873600
H	3.89244900	1.39209800	0.40019600
H	3.98899400	-1.27349300	-0.41289700
H	3.50340300	-0.79358500	1.21439500
H	-0.57399700	-2.03681100	0.28079800
H	-2.31625900	-1.69434600	-1.25657300
H	-3.14616600	1.96347300	0.09087900
H	-0.69643800	1.88291200	0.96614100
O	-3.05303900	0.04855400	-0.54666100

TS3

C	1.82662300	-1.49281600	-1.21882100
C	0.60492400	-2.39458900	-1.32935800
C	1.28075300	-3.16822800	0.81991300
C	1.21226000	-1.68811900	1.15674300
N	1.89497300	-0.91410400	0.12573000
H	2.74253900	-2.05383000	-1.43898800
H	-0.30146300	-1.79667600	-1.16131900
H	0.77399200	-3.74382000	1.59819200
H	0.16480100	-1.35515300	1.23190700
C	2.04999800	0.41484700	0.28919200
C	2.44002900	1.33907200	-0.67807000
C	1.94821000	1.07397500	1.64634500
C	2.75978200	2.65636000	-0.00585000
H	2.94829900	1.04375500	-1.58768800
C	1.96090200	2.57200200	1.30622100
H	2.83843700	0.79423700	2.22894800
H	1.06712900	0.77237900	2.21449000
H	3.83753500	2.73416200	0.19785200
H	2.48022200	3.52075400	-0.61573800
H	2.38444900	3.18069300	2.10858500
H	0.93255500	2.90383000	1.13098100
H	1.69312500	-1.52628200	2.12459900
H	2.33211200	-3.48719800	0.78696100
H	0.55259200	-2.82664300	-2.33103300
H	1.73023100	-0.69358500	-1.95641000
O	0.65010500	-3.48118400	-0.41451900
C	-2.00018900	0.60507700	-0.03938400
C	-2.95607200	0.78855600	-1.04918600

C	-4.29576200	0.50260500	-0.80220900
C	-4.70712900	0.02620500	0.44224600
C	-3.75674400	-0.15866800	1.44633000
C	-2.41632900	0.12841600	1.21071700
H	-2.64515500	1.15234500	-2.02351000
H	-5.02390900	0.65003800	-1.59436600
H	-5.75271300	-0.19776500	0.62649500
H	-4.06020300	-0.52563300	2.42221800
H	-1.67700700	-0.00377600	1.99514100
N	-0.62031900	0.83907900	-0.18993300
N	-0.27044100	1.43636400	-1.26288700
N	0.72065200	1.79287200	-1.78864300

P3

C	-0.17742000	1.80359300	-1.11952700
C	1.18287800	2.48437100	-1.04933100
C	0.72236700	3.06704400	1.21800400
C	0.37689100	1.58585400	1.25902300
N	-0.58283700	1.29207300	0.19893100
H	-0.92259500	2.52099900	-1.48503700
H	1.97226500	1.73844400	-0.88003300
H	1.48149800	3.29002700	1.97575700
H	1.29402500	0.98140300	1.19640700
C	-1.25387500	0.02205000	0.21026600
C	-2.48558300	-0.04507900	-0.74121300
C	-1.80159900	-0.37665400	1.59138100
C	-3.63942400	-0.64517400	0.08491300
H	-2.75082400	0.92781500	-1.16046900
C	-2.94959000	-1.33317900	1.26945200
H	-2.19549300	0.52598200	2.07215700
H	-1.04322400	-0.80322500	2.25021200
H	-4.27959700	0.16564900	0.44785800
H	-4.25565200	-1.31422100	-0.52010900
H	-3.61836800	-1.48740200	2.11983500
H	-2.55495100	-2.31033800	0.96948800
H	-0.07749600	1.37865000	2.23084200
H	-0.17732900	3.65454400	1.43918900
H	1.39299200	2.98470100	-1.99691700
H	-0.10858100	0.99743500	-1.86024500
O	1.22267200	3.48806900	-0.05106700
C	0.90424500	-1.35413800	-0.18142500
C	1.84828600	-1.10247900	-1.17976500
C	3.19968100	-1.32837000	-0.93302000
C	3.61996500	-1.78870600	0.31347500
C	2.67581400	-2.05184000	1.30497000
C	1.32143500	-1.85220600	1.05501900
H	1.52017000	-0.73149700	-2.14487500
H	3.92628500	-1.13135700	-1.71483700
H	4.67504100	-1.95171800	0.50764500
H	2.99167400	-2.43103700	2.27159300
H	0.58576100	-2.09241500	1.81393100
N	-0.47404500	-1.10271500	-0.40639700
N	-0.98242200	-1.45364600	-1.61689700
N	-2.08946900	-0.92535700	-1.85238000

R4

C	0.08565200	1.12454600	0.32321500
C	0.08522600	-1.12458600	0.32315800
C	0.03314800	-0.00003200	1.37670200
H	-0.89143600	0.00013000	1.96507100
H	0.89885600	-0.00020900	2.04484800
C	-1.18319000	0.77813400	-0.51977000
H	-2.08000100	1.17492900	-0.03344600
H	-1.13150200	1.20384900	-1.52518800
C	-1.18357200	-0.77772800	-0.51967700
H	-2.08050500	-1.17405700	-0.03319600
H	-1.13219200	-1.20358000	-1.52505300
H	0.11567100	2.15218100	0.68896800
H	0.11495300	-2.15224400	0.68887100
C	1.27661600	-0.66987700	-0.50160400
C	1.27703300	0.66948200	-0.50134900
H	1.92043900	1.32551200	-1.07758200
H	1.92023800	-1.32613800	-1.07733400

TS4

C	-2.26572000	0.86792000	-0.57815600
C	-2.73510400	0.35101000	0.77343900
C	-1.32193100	-1.19852300	-0.05430300
C	-1.41171900	-0.09230100	-1.08473900
H	-2.85874600	1.53544500	-1.19371200
H	-1.20548000	-0.24684300	-2.13655400
C	-1.47944800	-0.40104300	1.25212600
H	-1.67347200	-1.04301700	2.11715300
H	-0.63055900	0.24982800	1.47504600
C	-3.67950200	-0.84906400	0.46419100
H	-4.15581100	-1.20540300	1.38293000
H	-4.47132400	-0.57362500	-0.23766400
C	-2.70437800	-1.91845400	-0.11434300
H	-2.68608000	-2.81876600	0.50787600
H	-2.96236400	-2.22313800	-1.13244700
H	-3.15316700	1.09960800	1.44810200
H	-0.45838000	-1.86060200	-0.13558800
N	0.10362500	2.03880000	-0.31731400
N	-0.92688000	2.51034700	-0.04743200
N	0.56165100	1.00763300	-0.89343800
C	1.73827800	0.41375700	-0.39919700
C	2.40891500	0.84423900	0.75170800
C	2.20439700	-0.70394400	-1.09953200
C	3.53995300	0.15914500	1.18652800
C	3.32689700	-1.38810800	-0.64669600
C	4.00258700	-0.96023200	0.49632400
H	2.05073400	1.71460200	1.29276200
H	4.05896700	0.50347900	2.07559900
H	4.88244400	-1.49133900	0.84389100
H	3.67892300	-2.25730400	-1.19328600
H	1.67619400	-1.02680100	-1.99090900

P4

C	-1.92037300	0.93368200	-0.71015400
C	-2.94417600	0.50588700	0.35784000
C	-1.36909400	-1.11495100	0.46903700
C	-0.83951900	-0.17743700	-0.63706500
H	-2.38013900	1.07527200	-1.69140200
H	-0.64804300	-0.69333000	-1.58341000
C	-2.03634300	-0.11809200	1.43169900
H	-2.60127100	-0.61454600	2.22610500
H	-1.33694200	0.58942100	1.88667200
C	-3.67682600	-0.73577600	-0.19054500
H	-4.53098300	-0.98117300	0.44657100
H	-4.05789600	-0.57708800	-1.20380000
C	-2.58779300	-1.85083800	-0.12303500
H	-2.89606500	-2.66642400	0.53653100
H	-2.36984500	-2.28763000	-1.10277900
H	-3.59837200	1.31954200	0.67469700
H	-0.60755300	-1.76950300	0.89682600
N	-0.00221700	1.94386600	-0.12717500
N	-1.20490400	2.17807700	-0.36009100
N	0.31199800	0.62955200	-0.25241700
C	1.63447800	0.19486200	-0.12090300
C	2.64297300	1.07362100	0.30106600
C	1.95832900	-1.13603300	-0.41215000
C	3.94944000	0.61642500	0.42245500
C	3.27349700	-1.57708100	-0.28303300
C	4.27798800	-0.70874700	0.13367100
H	2.39700100	2.10350500	0.52761800
H	4.71978800	1.30801100	0.74945100
H	5.30059100	-1.05678000	0.23349100
H	3.50714800	-2.61188200	-0.51345600
H	1.19295600	-1.83098900	-0.73863400

R6

C	0.00000000	-1.22170200	-0.14349300
C	1.23072300	-0.31973900	0.10858900
C	0.66725300	1.07311400	-0.04888900
C	-0.66725300	1.07311400	-0.04888900
C	-1.23072300	-0.31973900	0.10858900
H	0.00000000	-2.12047900	0.47858500
H	2.05219700	-0.52860700	-0.58479200
H	1.63295000	-0.45309200	1.12240400
H	1.29070700	1.95986200	-0.10993200
H	-1.29070700	1.95986200	-0.10993200
H	-2.05219700	-0.52860700	-0.58479200
H	-1.63295000	-0.45309200	1.12240400
H	0.00000000	-1.54613100	-1.18939100

TS6

C	-1.90223700	-1.10821200	-1.33396000
C	-1.32411500	-1.71072900	-0.04018600
C	-1.76125100	-0.71941900	1.01306500
C	-2.79671900	0.06547700	0.54212300
C	-3.16203600	-0.35284500	-0.86914200
H	-2.11120300	-1.86016500	-2.09866200
H	-0.24201400	-1.86935800	-0.07913700
H	-1.78508600	-2.68446300	0.17860400
H	-1.53919600	-0.85508600	2.06426900
H	-3.50012100	0.56503200	1.19949500
H	-3.41999500	0.48991900	-1.51729700
H	-4.03080100	-1.02499100	-0.84146900
H	-1.18180700	-0.39697100	-1.75545200
C	1.19205400	0.44658400	0.41322100
C	1.71231500	0.94143300	-0.78897700
C	2.96029100	0.51045000	-1.22902800
C	3.68947400	-0.42340200	-0.49369100
C	3.16336800	-0.91997700	0.69878700
C	1.92450700	-0.48461000	1.15715800
H	1.14639800	1.66591900	-1.36641500
H	3.36173400	0.90514200	-2.15717500
H	4.65954400	-0.75869600	-0.84530700
H	3.72324100	-1.64517000	1.28081600
H	1.51018300	-0.85968700	2.08751200
N	-0.08231600	0.77619900	0.91042300
N	-0.74051400	1.70260800	0.34380200
N	-1.85785400	1.92977500	0.09348800

P6

C	2.59258100	-0.96551100	1.21058000
C	1.53125900	-1.60387700	0.30607000
C	1.20594300	-0.50745100	-0.72775100
C	2.43749300	0.43119600	-0.75811900
C	3.45464800	-0.16184700	0.23017300
H	3.16687400	-1.70444000	1.77478100
H	0.64299700	-1.94003900	0.84700900
H	1.95126000	-2.46954600	-0.21861600
H	0.92843400	-0.90701700	-1.70706200
H	2.86307600	0.59054400	-1.75124800
H	4.05251800	0.62048700	0.70392200
H	4.13562300	-0.82931900	-0.30931300
H	2.11586700	-0.29248600	1.93293300
C	-1.17776100	0.19357600	-0.11821500
C	-2.02759700	1.16683500	0.42789800
C	-3.38472900	0.90088800	0.56183200
C	-3.92047300	-0.32509600	0.16503100
C	-3.07322000	-1.28809100	-0.37458500
C	-1.71029900	-1.03800100	-0.51948500
H	-1.62032400	2.12050600	0.73964500
H	-4.03036500	1.66414900	0.98520500
H	-4.98097100	-0.52447800	0.27639500
H	-3.46905100	-2.24819900	-0.69085200
H	-1.07071700	-1.80367200	-0.94384800
N	0.19089800	0.43787500	-0.26701200
N	0.70539300	1.66882100	-0.03185800
N	1.92481000	1.73727500	-0.28106200

TS2*

C	-0.31108700	1.80362600	-0.92860200
C	-0.40039300	3.27267000	-0.49813700
C	-1.90631900	3.52878800	-0.39961700
C	-2.42912500	2.20793400	0.15693300
N	-1.58114800	1.21144500	-0.49770700
H	0.54705800	1.30748200	-0.46341800
H	0.06099900	3.39745800	0.48741200
H	-2.33002300	3.70920700	-1.39366400
H	-3.48399500	2.04620300	-0.08129700
C	-1.68770300	-0.13849000	-0.19878700
C	-0.75816100	-1.04820400	-0.72770900
C	-3.07090000	-0.59813000	0.22691600
C	-1.03757400	-2.53931400	-0.77292600
H	-0.04888700	-0.67227400	-1.45725400
C	-3.15556900	-2.10143600	0.47759700
H	-3.39262700	-0.05051300	1.11704800
C	-2.52829400	-2.86963500	-0.68191300
H	-0.60998300	-2.94833200	-1.69470900
H	-4.20488500	-2.38289000	0.61300700
H	-3.03481600	-2.59199600	-1.61601400
H	-0.19655200	1.70690600	-2.01674600
H	0.10934700	3.94171800	-1.19515300
H	-2.15845600	4.37799100	0.23972500
H	-2.31600600	2.17302200	1.25141300
H	-0.51567700	-3.03650700	0.05260600
H	-2.66047100	-3.94900600	-0.55248500
H	-2.63147200	-2.35132000	1.40896500
H	-3.76553000	-0.32765000	-0.58113000
C	2.16496800	-0.63569100	0.41348600
C	2.78796700	-1.29897700	-0.65163200
C	2.83977700	0.40311100	1.06977600
N	0.85243800	-1.03652400	0.71115800
C	4.06229500	-0.92054600	-1.05922500
C	4.12232900	0.76045200	0.66326600
N	0.24716000	-0.47958200	1.70130000
C	4.73910000	0.10846700	-0.40359300
N	-0.87417100	-0.16431700	1.86244300
H	2.26140200	-2.10734300	-1.14953400
H	2.36292900	0.91615300	1.89872000
H	4.53290700	-1.43929300	-1.88868300
H	4.63997300	1.56031300	1.18397300
H	5.73695800	0.39577300	-0.71814200

***trans*-cyclooctene**

C	-3.27770000	2.60932900	-0.62350600
H	-4.20824900	2.36961000	-0.10542500
C	-2.93640000	3.89284700	-0.76126000
H	-1.98030900	4.13069300	-1.23152000
C	-2.25668900	1.51999000	-0.68134500
H	-2.65560900	0.55535500	-1.01304900
H	-1.43689900	1.79760800	-1.35486100
C	-3.52319500	4.95437600	0.11122200
H	-4.54750100	4.69050900	0.40087000
H	-3.54728800	5.94641000	-0.35244000
C	-1.70570100	1.38573100	0.76560900
H	-0.85533100	0.69292000	0.74968600
H	-2.47287800	0.91498400	1.39465700
C	-1.26091600	2.70937200	1.42958500
H	-0.56770700	2.43616500	2.23345200
H	-0.66241200	3.28597800	0.71260600
C	-2.36431300	3.62486900	2.04802100
H	-2.07850500	3.83433200	3.08523500
H	-3.30959000	3.07083700	2.11287300
C	-2.62766200	4.99459100	1.38076800
H	-3.09576600	5.66291500	2.11397500
H	-1.66398000	5.45215500	1.12031800

cyclooctyne

C	-2.49482300	-0.17098400	0.25402200
C	-1.38443400	0.90494200	0.23326200
C	-0.66757800	-2.89003300	1.55871500
C	-0.03332700	0.45169900	-0.37216200
C	0.09021300	-1.88129700	0.81766500
C	0.28857400	-0.84214500	0.23039900
C	-2.58388800	-1.11142200	1.49412300
C	-2.16767300	-2.59034000	1.31905800
H	-2.42948200	-0.77366600	-0.66136900
H	-1.20868700	1.25897200	1.25625000
H	-0.12676300	0.34170600	-1.45902500
H	-0.43596900	-2.81660200	2.62786800
H	-3.44798800	0.36468300	0.18571500
H	-1.73261200	1.77106400	-0.34161400
H	-0.42899900	-3.91200100	1.24813500
H	0.73777300	1.20696200	-0.19113000
H	-2.01968500	-0.67482700	2.32867900
H	-3.63007700	-1.11934000	1.81901900
H	-2.74596200	-3.21183200	2.01274700
H	-2.43361400	-2.92054100	0.30764300

cyclopropene

C	0.58198900	1.33697700	-0.00268300
H	0.57502300	1.92713400	0.91626200
H	0.59886200	1.94072000	-0.91266700
C	-0.07619300	-0.02143200	-0.02172800
H	-1.01295600	-0.55365000	-0.03805900
C	1.21695300	-0.03252700	-0.00454500
H	2.14425500	-0.58128800	0.00335100

TS-cyclooctene

N	-5.89494000	3.09113500	2.17042900
N	-5.03962600	3.98768000	1.92497500
N	-4.00214500	4.35555100	2.29780000
C	-6.65886600	2.56652000	1.10932200
C	-6.42164900	2.85552100	-0.24016300
C	-7.67544400	1.67232000	1.46033300
C	-7.20356100	2.25373000	-1.22175600
C	-8.44011100	1.06581400	0.46945200
C	-8.21119800	1.35422500	-0.87578100
C	-3.46554500	2.73188500	3.67167800
C	-4.50793700	1.84887900	3.53298800
C	-2.09952500	2.37301000	3.16419800
C	-5.39449100	1.51471700	4.69018700
C	-1.37783700	1.53440200	4.24347000
C	-2.16954500	0.32565300	4.78428200
C	-3.24598700	0.60541900	5.87551100
C	-4.72142200	0.37265000	5.48979900
H	-5.63633200	3.55251600	-0.51578900
H	-7.85817600	1.46423400	2.50932900
H	-7.01880500	2.48840400	-2.26543400
H	-9.22506400	0.37123600	0.75162000
H	-8.81438600	0.88598200	-1.64651600
H	-3.48219900	3.37775500	4.55075900
H	-4.41464100	1.07396900	2.77121300
H	-2.18999600	1.78308800	2.24342000
H	-1.50110100	3.25963900	2.92837700
H	-6.39298300	1.19358500	4.37761500
H	-5.51390700	2.39382200	5.33434200
H	-1.11337900	2.18887400	5.08485700
H	-0.42883400	1.17892300	3.82386500
H	-1.43697400	-0.37006100	5.20831200
H	-2.62609400	-0.21090500	3.94213500
H	-3.13046500	1.62806600	6.25696300
H	-3.03585500	-0.04907900	6.72867000
H	-4.79291900	-0.55674100	4.90923000
H	-5.30706100	0.20857800	6.40230600

TS-cyclooctyne

C	4.12148100	-0.62538300	-0.63764200
C	4.14424600	0.74209400	0.07529700
C	0.83658700	-1.50574100	0.27214000
C	2.95594700	1.67221400	-0.22321500
C	1.03701400	-0.05517400	0.19967300
C	1.68115000	0.98552000	0.05105800
C	3.42169000	-1.81222800	0.07920700
C	2.01787500	-2.22286000	-0.40918100
N	-1.05502300	0.78483100	0.71618500
N	-0.72077000	1.96789000	0.45331200
N	0.21982400	2.60794100	0.23123900
C	-2.31278100	0.30172900	0.30546300
C	-2.69233300	-0.94476300	0.81274500
C	-3.14650800	0.97165100	-0.59797100
C	-3.89303000	-1.52095000	0.41028500
C	-4.35210700	0.39206300	-0.98070000
C	-4.72952100	-0.85600600	-0.48541600
H	3.69822600	-0.50268000	-1.64382900
H	4.20777000	0.58442800	1.15915700
H	2.97315400	1.97459600	-1.27779200
H	-0.10325700	-1.80393000	-0.20445600
H	5.16635300	-0.91576600	-0.79217700
H	5.05643900	1.27771300	-0.21330600
H	0.76870200	-1.80989100	1.32399500
H	3.04194300	2.59178000	0.36413100
H	3.38925000	-1.62222900	1.16055000
H	4.06585500	-2.68927000	-0.04628200
H	1.88140200	-3.29796500	-0.24414400
H	1.95077100	-2.06397200	-1.49239700
H	-2.04219400	-1.44700500	1.52169600
H	-2.85389000	1.94063700	-0.99035000
H	-4.17963500	-2.48976400	0.80691400
H	-4.99827400	0.91937600	-1.67544900
H	-5.66908500	-1.30369300	-0.79187000

TS-cyclopropene

C	-0.87031200	2.98202900	-1.15747000
C	0.42732600	2.27519600	-0.90995400
C	0.18796300	3.34573000	-0.15200200
N	-0.09098900	0.60280400	0.46333100
N	-0.51937300	1.34658800	1.39086800
N	-0.55241600	2.45097100	1.73749800
C	-0.94561200	-0.40963500	-0.02797700
C	-2.30503400	-0.49171100	0.28896600
C	-0.37887200	-1.32018600	-0.92260300
C	-3.08548100	-1.49029800	-0.28528500
C	-1.17176000	-2.30412700	-1.50185200
C	-2.52645100	-2.39619300	-1.18442000
H	-1.80154000	2.52550500	-0.80917700
H	-0.99366100	3.57700600	-2.06510900
H	1.19328000	1.72094900	-1.42490700
H	0.65480000	4.20495700	0.29936600
H	-2.74347900	0.21447900	0.98798500
H	0.67903500	-1.24597400	-1.15193900
H	-4.13847700	-1.55580900	-0.03029400
H	-0.72705900	-3.00790300	-2.19817400
H	-3.14088900	-3.17037900	-1.63185100

propyne

C	0.00000000	0.00000000	1.42475000
H	0.00000000	0.00000000	2.49097600
C	0.00000000	0.00000000	0.21773500
C	0.00000000	0.00000000	-1.24287200
H	0.00000000	1.02302800	-1.62955100
H	0.88596800	-0.51151400	-1.62955100
H	-0.88596800	-0.51151400	-1.62955100

1-methoxycyclopentene

C	-2.05366800	-0.41112600	0.19407200
C	-1.67063000	1.06105000	-0.09665900
C	-0.16273900	1.05382100	0.00056000
C	0.29987600	-0.20454800	-0.02098300
C	-0.79088400	-1.23574100	-0.12545600
H	-2.92757400	-0.74049100	-0.37348500
H	-2.14071300	1.75163100	0.61172200
H	-1.99699800	1.36854300	-1.10007100
H	0.43702500	1.95571000	0.02504500
H	-0.63470600	-2.07094100	0.56424800
H	-0.81129900	-1.65262100	-1.14090200
H	-2.29173900	-0.51688000	1.25737400
O	1.57000300	-0.66373700	-0.00327900
C	2.58533300	0.32277100	0.04807600
H	2.48818800	0.93218800	0.95457300
H	3.53604000	-0.20942000	0.06233400
H	2.53802200	0.97481500	-0.83226000

maleic anhydride

C	0.66565200	-1.26039300	0.00007300
C	-0.66565200	-1.26039300	-0.00009400
H	1.35739400	-2.09067700	0.00023900
H	-1.35739400	-2.09067700	-0.00027900
O	0.00000000	0.96350200	-0.00005600
C	1.12275800	0.15677600	-0.00022000
C	-1.12275800	0.15677600	0.00029000
O	2.23020300	0.60729700	0.00007000
O	-2.23020200	0.60729700	-0.00004500

N-phenylmaleimide

C	2.98612000	-0.64470500	-0.16668500
C	2.98634900	0.64415900	0.16642100
H	3.82108100	-1.30925200	-0.34007900
H	3.82148600	1.30843300	0.34000400
C	1.56648700	-1.10994400	-0.28846200
C	1.56679600	1.10980600	0.28852900
O	1.17191500	-2.21533200	-0.57975700
O	1.17267300	2.21548800	0.57938500
N	0.76316200	-0.00002500	0.00043900
C	-0.66160600	0.00008900	0.00022800
C	-1.34923000	0.90496700	-0.80571700
C	-1.34949900	-0.90478200	0.80601100
C	-2.74140400	0.90755900	-0.79562000
H	-0.79828000	1.60171400	-1.42764900
C	-2.74162600	-0.90737900	0.79549900
H	-0.79866900	-1.60144400	1.42814200
C	-3.43898800	0.00009600	-0.00018600
H	-3.28013100	1.61570300	-1.41653400
H	-3.28057800	-1.61549300	1.41625100

H	-4.52414200	0.00007600	-0.00035200

TS-methoxycyclopentene

C	-1.60674500	-0.03803900	-0.28787600
C	-2.04640800	1.00802600	0.53348800
C	-2.52663500	-0.99513500	-0.73771500
N	-0.23360900	-0.05867000	-0.57046200
C	-3.38506700	1.09270700	0.90105700
C	-3.86528300	-0.89409700	-0.37024600
N	0.21863200	-0.91576900	-1.39099400
C	-4.30379100	0.14424900	0.45122900
N	1.29735600	-1.28859600	-1.68174500
H	-1.32724800	1.74842100	0.87039800
H	-2.19082500	-1.80842000	-1.37326000
H	-3.71263000	1.90831000	1.53848500
H	-4.57075500	-1.63805100	-0.72800900
H	-5.34856500	0.21401700	0.73557900
C	1.81585500	-1.37708700	1.69175400
C	1.62778800	0.14535900	1.77710900
C	1.93903600	0.58502900	0.37364900
C	2.60326000	-0.39814100	-0.35193100
C	2.87040100	-1.56439400	0.58136400
H	0.87263000	-1.84183100	1.38691000
H	2.37187300	0.59941600	2.44657700
H	0.63775600	0.45958700	2.11360600
H	3.27489800	-0.15448000	-1.16650700
H	3.88464200	-1.48549400	0.99613800
H	2.79265400	-2.53516200	0.08376100
H	2.11878600	-1.81952400	2.64322100
O	1.82152100	1.88201700	0.09616200
C	2.08216900	2.28257300	-1.24318600
H	1.84339000	3.34360800	-1.29539100
H	3.13520400	2.12886200	-1.50007000
H	1.44189700	1.71955000	-1.93050400

TS-maleic anhydride

C	-1.42423800	-0.61453000	-0.27034100
C	-2.31620900	-0.03423100	-1.17042200
C	-1.73364300	-0.70808600	1.08848700
N	-0.17256200	-1.03073200	-0.79760900
C	-3.53186700	0.45619800	-0.70536500
C	-2.96028400	-0.23155100	1.53794100
N	0.53269200	-1.87464600	-0.17137700
C	-3.85810100	0.35457400	0.64602300
N	1.65948100	-2.08469200	0.03478800
H	-2.04992400	0.03242500	-2.21982000
H	-1.02711200	-1.15304300	1.78242400
H	-4.22692900	0.91316100	-1.40154700
H	-3.21007300	-0.31010900	2.59070200
H	-4.80966600	0.73224700	1.00482000
C	1.30628800	0.33346500	-1.26911900
C	2.45429500	-0.30555800	-0.82347900
H	0.97261700	0.43138900	-2.29142800
H	3.17515900	-0.84158200	-1.42371900
O	1.87086000	1.27169500	0.78148200
C	2.84703200	0.33090500	0.45233500
C	0.98606600	1.39253500	-0.26840600
O	3.79809300	0.15541200	1.15662000
O	0.13387300	2.23335200	-0.28909900

TS-N-phenylmaleimide

C	-2.50280500	-0.02689200	-0.21581200
C	-3.22190700	-0.54707600	0.85939000
C	-2.09431000	-0.85235600	-1.26731600
N	-2.15213400	1.34616300	-0.14099700
C	-3.53114500	-1.90276900	0.88384600
C	-2.42532000	-2.20314800	-1.24036400
N	-1.74903000	1.93289100	-1.18915400
C	-3.13790700	-2.73279100	-0.16489300
N	-0.94283200	2.71840100	-1.49198300
H	-3.51902600	0.10956900	1.66975800
H	-1.53019300	-0.44115300	-2.09907100
H	-4.08342200	-2.31029400	1.72389700
H	-2.11759300	-2.84404500	-2.06016800
H	-3.38610900	-3.78871200	-0.14529200
C	0.11414300	2.83605000	0.32622100
C	-0.62427300	2.02057500	1.16734800
H	0.11732100	3.91686600	0.31825000
H	-1.32219800	2.33814900	1.92781200
C	0.09846700	0.71857800	1.30240800
C	1.30887200	2.06493500	-0.12933700
O	-0.17704300	-0.21048000	2.02758400
O	2.22031500	2.43915500	-0.83106400
N	1.18237300	0.77406200	0.41858500
C	2.06827500	-0.31298100	0.17256200
C	3.44344500	-0.14058400	0.31532600
C	1.53939700	-1.54567600	-0.20777400
C	4.29515200	-1.21320500	0.06321700
H	3.84005200	0.82323100	0.61239300
C	2.39769300	-2.61582100	-0.44061500
H	0.46586900	-1.66364700	-0.31193900
C	3.77603000	-2.45123600	-0.31084700
H	5.36702600	-1.08000300	0.16662000
H	1.98719300	-3.57779600	-0.72987400
H	4.44341700	-3.28582000	-0.49987100

12. Cartesian coordinates of the structures (optimization in carbon tetrachloride)

Phenyl azide

C	0.00000000	0.39946000	0.00000000
C	-1.31103000	0.87984500	0.00000000
C	0.24802300	-0.97540900	0.00000000
N	1.02302300	1.38752200	0.00000000
C	-2.37259500	-0.01796700	0.00000000
C	-0.82474000	-1.86401300	0.00000000
N	2.19054700	0.99078200	0.00000000
C	-2.13573800	-1.39281400	0.00000000
N	3.29770200	0.74934300	0.00000000
H	-1.48202200	1.95103700	0.00000000
H	1.26505900	-1.35649000	0.00000000
H	-3.39004200	0.35959700	0.00000000
H	-0.62913400	-2.93154700	0.00000000
H	-2.96628200	-2.09074000	0.00000000

R5

C	-0.00003500	1.11963700	0.27293700
C	0.00003500	-1.11963700	0.27293700
C	0.00000000	-0.00000100	1.35160500
H	-0.89924200	-0.00003200	1.97417700
H	0.89924000	0.00003100	1.97417800
C	-1.23909200	0.66746000	-0.52003100
C	-1.23911800	-0.66758000	-0.51993800
H	-0.00010000	2.15532500	0.61302300
H	0.00010000	-2.15532600	0.61302000
C	1.23909400	-0.66745900	-0.52002900
C	1.23911800	0.66758000	-0.51993700
H	1.92860300	1.33661200	-1.02030700
H	1.92923900	-1.33646400	-1.01952400
H	-1.92860400	-1.33661000	-1.02030800
H	-1.92924300	1.33646400	-1.01952100

TS5

C	-1.40014900	-1.26582700	-0.05991800
C	-2.81929400	0.25728000	0.77688200
C	-1.51341600	-0.43499500	1.24194300
H	-0.67912500	0.24913700	1.41085900
H	-1.65931900	-1.06064100	2.12493600
C	-1.48650000	-0.13591900	-1.09348300
C	-2.34504600	0.80989100	-0.57979700
H	-0.55322500	-1.94447200	-0.16107900
H	-3.27795100	0.98084900	1.45045400
C	-3.64261800	-1.00651600	0.46439300
C	-2.79830800	-1.91355100	-0.03553700
H	-3.02532000	-2.90255200	-0.41454200
H	-4.71493300	-1.09420400	0.59054300
H	-2.93386000	1.49622300	-1.17535700
H	-1.24997300	-0.27261200	-2.13959300
C	1.67777000	0.41235100	-0.39936300
C	2.17866600	-0.68028200	-1.11479300
C	3.31263900	-1.34511200	-0.66151900
C	3.96443700	-0.92232300	0.49717300
C	3.46645500	0.17180000	1.20304900
C	2.32392300	0.83693800	0.76752900
H	1.66890200	-0.99850000	-2.01850900

H	3.69262100	-2.19456600	-1.22022100
H	4.85320500	-1.43821200	0.84495500
H	3.96666300	0.51146100	2.10456600
H	1.93831000	1.68776500	1.32076200
N	0.49121000	0.98746100	-0.89419900
N	0.01889100	2.01086700	-0.31628900
N	-1.01543500	2.46994500	-0.04053100

P5

C	-1.41009100	-1.17183600	0.47581100
C	-3.01551700	0.40587700	0.37348700
C	-2.05117300	-0.15282700	1.43762400
H	-1.34899300	0.58584200	1.83523900
H	-2.57651900	-0.63647400	2.26378800
C	-0.89963300	-0.21939400	-0.65885600
C	-2.00213900	0.87006100	-0.72134800
H	-0.64818700	-1.83743300	0.88185000
H	-3.71644400	1.18311000	0.67649400
C	-3.60848700	-0.88786600	-0.16243700
C	-2.65529700	-1.82592000	-0.10045700
H	-2.70518000	-2.83946900	-0.48095100
H	-4.59379400	-0.98015100	-0.60433500
H	-2.48134900	1.00354300	-1.69225700
H	-0.71176100	-0.74227200	-1.59936000
C	1.56523700	0.19515600	-0.14006300
C	1.91392300	-1.13002900	-0.42756100
C	3.23418200	-1.54981000	-0.28129600
C	4.21861700	-0.66524900	0.14899700
C	3.86501000	0.65427400	0.43381300
C	2.55300900	1.09032100	0.29508400
H	1.16432500	-1.83662700	-0.76548900
H	3.48780200	-2.58046500	-0.50902700
H	5.24530500	-0.99669600	0.26226400
H	4.61967400	1.35791000	0.77139200
H	2.28666400	2.11580500	0.51864800
N	0.23811000	0.60964500	-0.29352500
N	-0.10471600	1.91354500	-0.13867900
N	-1.31445000	2.12350100	-0.36709000

R7

C	-0.77384700	-1.25077100	-0.66968400
C	-1.28742300	-0.00110300	0.08820600
C	1.28742200	0.00110500	0.08822100
C	0.77607100	-1.24925600	-0.66996800
H	-1.16263700	-2.15489200	-0.19161200
H	-1.16503400	-1.23381400	-1.69332600
H	1.16678800	-2.15280000	-0.19238000
H	1.16688900	-1.23111700	-1.69373000
C	-0.66897500	-0.00027500	1.46421800
C	0.66895900	0.00030200	1.46422500
H	2.38007600	0.00205100	0.12851000
H	-2.38007700	-0.00204700	0.12848300
C	0.77385400	1.25075600	-0.66970200
H	1.16264200	2.15488800	-0.19165000
H	1.16505000	1.23377300	-1.69334100
C	-0.77606300	1.24924300	-0.66999900
H	-1.16678300	2.15279500	-0.19242800
H	-1.16687300	1.23109000	-1.69376500
H	1.27448300	0.00053600	2.36607300
H	-1.27450900	-0.00047100	2.36605900

TS7

C	-1.30102100	-0.17514900	-1.19321500
C	-1.29869900	-1.33233300	-0.22529900
C	-2.90284300	0.57552700	0.45253900
C	-2.13937000	0.86030500	-0.82535200
H	-0.99800700	-0.32875800	-2.22203300
H	-2.56794700	1.51903100	-1.57469800
C	-0.96389500	-0.85692600	1.20194100
H	-1.05632400	-1.70943100	1.88339100
H	0.07710900	-0.52789700	1.24974100
C	-1.92982000	0.28405200	1.61121300
H	-2.51102600	0.00249000	2.49612600
H	-1.37740600	1.19098600	1.86948600
H	-3.56578700	1.40502300	0.71260000
H	-0.60606200	-2.11807600	-0.53882700
C	-3.72197100	-0.71627800	0.19531800
H	-4.27638900	-0.97606600	1.10401400
H	-4.46178500	-0.53389600	-0.59045100
C	-2.75920600	-1.86513300	-0.20865400
H	-2.82140900	-2.69622400	0.50238400
H	-3.01880400	-2.26610200	-1.19330700
C	1.91210800	0.37188900	-0.43898100
C	2.38492600	-0.80017200	-1.03872600
C	3.54655000	-1.40176600	-0.56604400
C	4.25339300	-0.83915200	0.49671800
C	3.78288700	0.33316700	1.08679200
C	2.61556300	0.93816600	0.63139300
H	1.83256800	-1.22957400	-1.86846300
H	3.90427200	-2.31266900	-1.03572100
H	5.16146700	-1.30846900	0.86026900
H	4.32396600	0.78098100	1.91455100
H	2.24905300	1.84694700	1.09803200
N	0.70029100	0.88319900	-0.93694600
N	0.23451800	1.95833300	-0.45432900
N	-0.81467900	2.44482800	-0.28657400

P7

C	-0.69888000	-0.22122300	-0.82402200
C	-1.08381100	-1.25338900	0.25146800
C	-2.94745600	0.54804200	0.03958300
C	-1.80128000	0.85927900	-0.93369500
H	-0.51723300	-0.72052800	-1.78259900
H	-2.19692500	0.95300300	-1.94981900
C	-1.29220800	-0.55399000	1.60123100
H	-1.54422000	-1.30930500	2.35226200
H	-0.35538400	-0.09000800	1.92590900
C	-2.42017000	0.50591500	1.48126800
H	-3.25186100	0.26535200	2.15136600
H	-2.05562700	1.49533000	1.77156300
H	-3.71984900	1.31648800	-0.05934300
H	-0.30202700	-2.01320800	0.34233900
C	-3.51040000	-0.83140400	-0.33851100
H	-4.35564000	-1.06380200	0.31686400
H	-3.90211500	-0.80458500	-1.36128600
C	-2.39838500	-1.90864400	-0.20314800
H	-2.68425600	-2.67240600	0.52677000
H	-2.24322500	-2.42778100	-1.15556400
C	1.75392600	0.22455800	-0.23342800
C	2.17405900	-1.04989300	-0.63275200
C	3.48243000	-1.46018900	-0.38819000
C	4.38731200	-0.61210600	0.24400800
C	3.96538100	0.65940400	0.63419100
C	2.66067300	1.08100700	0.40723100
H	1.48974100	-1.71996000	-1.14093100
H	3.79218700	-2.45179600	-0.70330100
H	5.40579000	-0.93533700	0.43145300
H	4.65764100	1.33302200	1.12976700
H	2.33690700	2.06644700	0.71901200
N	0.43794900	0.62879300	-0.47817400
N	0.08558500	1.93321800	-0.36382500
N	-1.12082800	2.13154300	-0.60741100

R8

C	3.27696100	-0.83319000	-0.11274400
H	3.05033400	-1.67756700	-0.75492600
H	4.28212200	-0.72472300	0.27487600
C	2.34677100	0.06452300	0.21206700
H	2.56550400	0.91758400	0.85460900
O	1.06635800	-0.02572000	-0.21946100
C	0.22713100	1.08667200	0.09055200
H	0.60288600	1.97911300	-0.42639100
H	0.25729800	1.27368200	1.17354000
C	-1.18759000	0.76695000	-0.35287700
H	-1.79249100	1.67125500	-0.20788800
H	-1.18259600	0.55976600	-1.43026100
C	-1.82079200	-0.40297700	0.40122600
H	-1.20233300	-1.29659300	0.26506400
H	-1.81728400	-0.18061800	1.47637600
C	-3.24928400	-0.68786500	-0.05947100
H	-3.68543100	-1.52567400	0.49270800
H	-3.89413900	0.18531700	0.09071000
H	-3.27391200	-0.94046300	-1.12525100

TS8

C	-2.89149600	-1.93400700	-0.00567500

H	-3.62471700	-1.52787900	-0.69240800
H	-3.03270900	-2.95359100	0.33342700
C	-2.23419000	-1.06886600	0.84504400
H	-1.78627900	-1.39680400	1.77754400
O	-2.28384300	0.24142300	0.62004500
C	-1.44790400	1.09495000	1.41841600
H	-0.82411600	0.47878400	2.07412300
H	-2.11388300	1.70721000	2.03587900
C	-0.58479300	1.96097800	0.51892700
H	0.08302200	2.54074900	1.16977600
H	0.05683900	1.31298600	-0.08880000
C	-1.37867100	2.90649000	-0.38207700
H	-2.05993100	2.32080100	-1.00900100
H	-2.00614200	3.55930400	0.23883100
C	-0.46856200	3.75603900	-1.26754100
H	-1.04948100	4.42464800	-1.90974700
H	0.20611400	4.37229700	-0.66287300
H	0.14925000	3.12230300	-1.91319600
C	1.78116400	-0.83586400	1.27317200
C	3.01178900	-0.19711800	1.38106300
C	3.71377700	0.18368500	0.23736700
C	3.16801600	-0.08354100	-1.01774400
C	1.93139700	-0.71093900	-1.13744700
C	1.22690800	-1.09109500	0.01302000
H	1.23569500	-1.14206200	2.16068400
H	3.42741000	-0.00219700	2.36493700
H	4.67515900	0.67924500	0.32297300
H	3.70508500	0.20531000	-1.91611200
H	1.50557800	-0.90384500	-2.11697800
N	-0.05702700	-1.66174000	-0.00180300
N	-0.52371200	-2.07348500	-1.11395700
N	-1.59651100	-2.35233500	-1.51117900

P8

C	-3.33338400	0.29892800	0.04609200
H	-3.68402900	1.31211700	-0.15125600
H	-4.06841600	-0.22713900	0.66407000
C	-1.94453300	0.27577900	0.67616600
H	-1.94850400	0.10577900	1.75691700
O	-1.26082600	1.45417200	0.35958600
C	-0.29454700	1.96273200	1.28043300
H	-0.42233800	1.47818100	2.25765700
H	-0.52982900	3.02657300	1.40008100
C	1.13997000	1.80788200	0.79106900
H	1.78329100	2.33433200	1.50968500
H	1.43679500	0.75465700	0.81804000
C	1.36994800	2.36447300	-0.61264500
H	0.71147400	1.83875100	-1.31271100
H	1.07615800	3.42221700	-0.64103000
C	2.82184400	2.21573300	-1.06222700
H	2.96850400	2.60655600	-2.07381200
H	3.50118400	2.75438900	-0.39184300
H	3.11885100	1.16096600	-1.06148200
C	0.46345200	-1.71800800	1.24211700
C	1.80848200	-2.06325900	1.33201300
C	2.62226200	-2.01527800	0.20055400
C	2.08230200	-1.61691700	-1.02119700
C	0.74327200	-1.24699800	-1.11524200
C	-0.06534700	-1.29705700	0.02059900
H	-0.17893500	-1.77012700	2.11564600
H	2.21806400	-2.38086600	2.28552300
H	3.66987800	-2.28912900	0.27036400
H	2.71011700	-1.57524700	-1.90561200
H	0.32385700	-0.91101300	-2.05676900
N	-1.42343800	-0.89970900	-0.02534000
N	-2.11583900	-1.08789100	-1.19431000
N	-3.18669900	-0.45274900	-1.21271400

R9

C	3.09823500	0.79629200	-0.24409500
H	3.08985400	0.85075100	-1.33069200
H	3.77514900	1.46519800	0.27910100
C	2.31232000	-0.05662000	0.41334400
H	2.34793700	-0.07282500	1.50409000
C	1.34909600	-1.01969800	-0.22060100
H	1.65404800	-2.04269700	0.03572600
H	1.40666400	-0.93437800	-1.31312200
C	-0.10334100	-0.81297800	0.23858100
H	-0.70897100	-1.63830200	-0.15262700
H	-0.15085200	-0.88519800	1.33450600
C	-0.69402700	0.52565900	-0.20663300
H	-0.03743100	1.33572900	0.13329900
H	-0.69168100	0.56952500	-1.30541500
C	-2.11359700	0.78819200	0.30659400
H	-2.10797200	0.76988300	1.40460700
H	-2.40744700	1.80463300	0.01837800
C	-3.16039500	-0.19857100	-0.21341500
H	-2.97331600	-1.21599400	0.14431300
H	-4.16526300	0.08519400	0.11446600
H	-3.16047000	-0.22516900	-1.30928000

TS9

C	-1.00585100	2.37779500	0.25931000
H	-1.47571800	2.54555400	-0.70479000
H	-1.10644200	3.17284500	0.99132400
C	-0.74223500	1.08688100	0.67340300
H	-0.62179800	0.89806300	1.73691400
C	-1.02108700	-0.11661700	-0.18673500
H	-0.23048200	-0.86203100	-0.05035000
H	-1.00377600	0.17873300	-1.24345500
C	-2.37071000	-0.78079000	0.14154200
H	-2.41890800	-1.72570600	-0.41140600
H	-2.39261600	-1.04483500	1.20829000
C	-3.59099100	0.07783200	-0.19342500
H	-3.51102500	1.03815600	0.32966000
H	-3.5093000	0.31066700	-1.26809100
C	-4.93047600	-0.57151000	0.17054800
H	-4.94598300	-0.78369400	1.24789100
H	-5.73108800	0.15564300	-0.01020500
C	-5.23851500	-1.85313200	-0.60565900
H	-4.52897500	-2.65262900	-0.37025700
H	-6.24067000	-2.22390500	-0.36926000
H	-5.19345800	-1.67462800	-1.68622800
C	2.34077600	0.10232700	0.24168800
C	3.16966400	0.04799000	-0.88561400
C	3.94545900	-1.08400600	-1.11624800
C	3.89272200	-2.17227000	-0.24583400
C	3.05987900	-2.11666300	0.87133000
C	2.29203500	-0.98418700	1.12186100
H	3.21090900	0.89142700	-1.56752600
H	4.59263700	-1.11537800	-1.98722900
H	4.49685600	-3.05336200	-0.43498800
H	3.01273800	-2.95591500	1.55805800
H	1.64945400	-0.92871200	1.99490300
N	1.48806600	1.18070300	0.53709600
N	1.56540800	2.24857200	-0.14277100
N	0.83442000	3.10612600	-0.45721900

P9

C	-0.83393100	2.16913200	0.43155400
H	-1.69173800	2.36740100	-0.21429700
H	-1.09141500	2.48906200	1.44578100
C	-0.34472200	0.71172400	0.35751700
H	-0.45333900	0.20986900	1.32589200
C	-0.99178100	-0.13456800	-0.75035300
H	-0.26854800	-0.88043100	-1.09556500
H	-1.20278500	0.51344500	-1.61045700
C	-2.26425800	-0.85950600	-0.30321400
H	-2.61088400	-1.47494900	-1.14041900
H	-2.01097200	-1.55821200	0.50730700
C	-3.39861400	0.05481600	0.16659100
H	-3.05925200	0.65006200	1.02384400
H	-3.64250700	0.77070200	-0.63144900
C	-4.67111200	-0.69527500	0.57632400
H	-4.42595900	-1.41044300	1.37265000
H	-5.37311600	0.02364800	1.01471500
C	-5.36431300	-1.42778000	-0.57358400
H	-4.74455600	-2.23420100	-0.97776500
H	-6.30629100	-1.87427300	-0.24105300
H	-5.59187400	-0.73786600	-1.39421000
C	2.11220200	0.02927700	0.06535000
C	3.37945800	0.37374700	-0.42698700
C	4.39613000	-0.57347600	-0.43942100
C	4.17632200	-1.87073100	0.02510000

C	2.91673000	-2.20876400	0.51098300
C	1.88758300	-1.27040900	0.53690500
H	3.55736600	1.37772500	-0.79174500
H	5.37212900	-0.29176400	-0.82243600
H	4.97492300	-2.60471900	0.00731100
H	2.72467800	-3.21162300	0.87958300
H	0.91704300	-1.55643000	0.92698000
N	1.07650900	0.96913200	0.09838700
N	1.31914900	2.27544000	-0.16613600
N	0.30847700	2.99026700	-0.02443000

R10

C	-0.83302100	-0.68332800	-0.00000300
H	-0.87336500	-1.77190400	0.00033300
C	0.51903500	-0.09862200	-0.00004200
C	-1.98049800	0.00446600	-0.00004400
H	-2.93695900	-0.50808000	0.00013100
H	-2.00686400	1.09044700	-0.00015500
C	1.59034200	-0.90589800	-0.00002400
H	1.48313400	-1.98736200	-0.00003900
H	2.60137300	-0.50943400	-0.00001500
C	0.65255100	1.40160000	0.00004300
H	0.17008700	1.83729500	-0.88183800
H	0.16968700	1.83723700	0.88171700
H	1.70245600	1.70249200	0.00028600

TS10

C	0.80455400	2.58232200	-0.20758100
H	0.77086200	2.78485200	-1.27451500
H	0.30072300	3.28704700	0.44681300
C	1.44394500	1.50850900	0.27864400
C	2.10466000	0.57089300	-0.64374000
H	1.98461500	0.78343800	-1.70156900
C	3.08036800	-0.33706200	-0.26127100
H	3.47714800	-0.32268400	0.74772600
H	3.73304200	-0.75506700	-1.02039600
C	1.47769700	1.19833200	1.75153100
H	1.07683400	0.19643900	1.94430300
H	2.50025200	1.21961700	2.14355200
H	0.88299400	1.91925900	2.31627500
C	-0.91928800	-0.61697100	-0.42154700
C	-1.58550300	0.33909300	-1.19410800
C	-2.86622900	0.74909000	-0.83760400
C	-3.49606200	0.20367300	0.28012800
C	-2.83045700	-0.75395300	1.04572800
C	-1.54328600	-1.15957200	0.70884100
H	-1.08906900	0.75134400	-2.06607900
H	-3.37597200	1.49356800	-1.44096200
H	-4.49822300	0.51892900	0.55133600
H	-3.31428800	-1.18727000	1.91549800
H	-1.02655000	-1.90254700	1.30871100
N	0.39710500	-0.92167200	-0.80910700
N	1.00514700	-1.88300900	-0.24633500
N	2.10563900	-2.12289900	0.07475300

P10

C	2.04548100	2.49249700	-0.17368500
H	2.08855900	2.70475200	-1.23912600
H	2.26386900	3.31283800	0.50344100
C	1.73888700	1.27650100	0.28216200
C	1.51149400	0.13510800	-0.69226200
H	1.36899600	0.52870300	-1.70287300
C	2.62892800	-0.92882900	-0.66154500
H	3.49252100	-0.62825400	-0.06378700
H	2.98335100	-1.19378900	-1.66152500
C	1.64017800	0.93780700	1.74377600
H	0.61749600	0.64070900	2.00146300
H	2.29149500	0.09515300	2.00357000
H	1.91723100	1.79133100	2.36561900
C	-0.96010900	-0.33933400	-0.19445500
C	-1.36706200	0.91912900	-0.65255600
C	-2.70097000	1.30316400	-0.53412600
C	-3.64100700	0.44818800	0.03331200
C	-3.22908700	-0.80555800	0.48631100
C	-1.90221100	-1.20475600	0.37988200
H	-0.65090800	1.60314500	-1.09199800
H	-3.00033200	2.28313900	-0.89252400
H	-4.67849600	0.75219700	0.12292900
H	-3.94827200	-1.48557900	0.93227300
H	-1.59010500	-2.17894800	0.73475900
N	0.38084300	-0.72364400	-0.32382400
N	0.78724200	-1.95195300	0.08360900
N	2.01155200	-2.12853200	-0.06165600

R11

C	-0.25468400	0.72154700	-1.18723900
C	0.25468400	1.47701200	0.04448100
C	0.12132100	0.65712200	1.30277200
C	-0.12132100	-0.65712200	1.30277200
C	-0.25468400	-1.47701200	0.04448100
C	0.25468400	-0.72154700	-1.18723900
H	1.30938400	1.75490300	-0.09506700
H	-0.28948200	2.42148400	0.16295500
H	-1.35272100	0.71295500	-1.17798500
H	0.05257000	1.24011000	-2.10141000
H	-0.22839500	-1.17989000	2.25142000
H	0.28948200	-2.42148400	0.16295500
H	-1.30938400	-1.75490300	-0.09506700
H	1.35272100	-0.71295500	-1.17798500
H	-0.05257000	-1.24011000	-2.10141000
H	0.22839500	1.17989000	2.25142000

TS11

C	2.17783000	2.00791000	0.49414900
C	1.12685100	1.56099300	-0.52968700
C	1.49336800	0.26683300	-1.22613900
C	2.49298900	-0.57730500	-0.76014400
C	3.36577200	-0.19457700	0.41658000
C	2.67393400	0.82270900	1.32310600
H	0.15785800	1.45480700	-0.02633300
H	0.98054900	2.33799000	-1.28791600
H	3.03359000	2.45639800	-0.02723900
H	1.75433200	2.78560000	1.13793600
H	2.92309100	-1.27504200	-1.47544900
H	3.64694000	-1.09052500	0.97908800
H	4.29913700	0.23983100	0.03184100
H	1.82426800	0.34598700	1.83075900
H	3.36489600	1.15990100	2.10257800
H	1.16633300	0.15407000	-2.25327300
C	-1.52141700	-0.54027000	-0.29164300
C	-2.22514500	0.27978300	-1.18081300
C	-2.02295600	-0.75795000	0.99794100
N	-0.29218700	-1.04327300	-0.75152600
C	-3.41575000	0.87755000	-0.78211700
C	-3.22421300	-0.16781000	1.37973200
N	0.33860300	-1.87968600	-0.03073700
C	-3.92410500	0.65456800	0.49753900
N	1.46674200	-2.11881900	0.17391700
H	-1.82505100	0.44280400	-2.17647000
H	-1.47923000	-1.39492600	1.68863500
H	-3.95258400	1.51471700	-1.47785200
H	-3.61180300	-0.34924400	2.37738000
H	-4.85737400	1.11546200	0.80371000

P11

C	2.55076000	1.77139800	0.61567600
C	1.11133900	1.26900700	0.49392000
C	0.94080200	0.27725900	-0.67181500
C	2.05300900	-0.77184500	-0.78190500
C	3.46411100	-0.32721900	-0.41780600
C	3.50862000	0.59338100	0.80149300
H	0.83814800	0.75737500	1.42521500
H	0.41179900	2.10138900	0.37496000
H	2.83182000	2.33702300	-0.28351300
H	2.62246300	2.46401200	1.46015200
H	2.05703800	-1.18349700	-1.80189700
H	4.07643700	-1.22141700	-0.26370900
H	3.88666300	0.20352500	-1.27969900
H	3.22488200	0.03392700	1.70233500
H	4.53287700	0.94812000	0.95454300
H	0.81104000	0.79803300	-1.62264800
C	-1.50731200	-0.26164900	-0.19248300
C	-1.98521700	0.92565600	-0.75909100
C	-2.37200900	-1.05942700	0.56762400
N	-0.17583100	-0.64822700	-0.42972900
C	-3.30686100	1.31322900	-0.55659800

C	-3.69203300	-0.66235000	0.75409400
N	0.30454300	-1.77558200	0.17880900
C	-4.17026700	0.52425300	0.19937500
N	1.54104400	-1.88077600	0.06203700
H	-1.33187600	1.54679100	-1.36220700
H	-2.00719800	-1.98034900	1.00482100
H	-3.66032500	2.23870600	-1.00044000
H	-4.35138000	-1.28966100	1.34592600
H	-5.20033500	0.82795900	0.35342600

R12

C	0.76879100	-0.45848500	-0.00035000
H	0.80249700	-1.54406400	-0.00021100
C	1.87978300	0.28437800	-0.00035100
H	1.76730100	1.36784300	-0.00018000
C	3.27099100	-0.25782100	0.00025100
H	3.28418800	-1.35045100	-0.00072900
H	3.81863300	0.10163600	-0.87775900
H	3.81716100	0.09985700	0.87995900
C	-0.56740900	0.17247400	-0.00009700
O	-1.53936600	-0.75489100	0.00004600
O	-0.79341100	1.36783800	0.00009800
C	-2.88182200	-0.26077900	0.00009200
H	-3.06474500	0.34544900	0.88982300
H	-3.06657700	0.34148000	-0.89198000
H	-3.51824400	-1.14392400	0.00266100

TS12

C	1.83631700	-0.34802400	0.83033000
H	2.09594600	-0.05390600	1.84112600
C	0.70839600	-1.12266500	0.59213800
H	0.70121300	-1.71025300	-0.32125400
C	-0.16780500	-1.58675700	1.72048900
H	-0.23390300	-0.83183200	2.50879900
H	-1.17749300	-1.82741200	1.37978500
H	0.25920800	-2.49720400	2.15539100
C	2.90646200	-0.29693700	-0.18051300
O	4.03089900	0.24159400	0.31859400
O	2.80787000	-0.67749000	-1.33382900
C	5.12395900	0.37195000	-0.59406800
H	5.42793500	-0.60732600	-0.97007000
H	4.84493600	1.01153700	-1.43414700
H	5.93007400	0.82660400	-0.02073500
C	-1.95382400	0.24758300	-0.30162800
C	-2.53606900	-0.87649900	-0.89290500
C	-2.75671500	1.21863000	0.30539400
N	-0.54446200	0.30193300	-0.31002200
C	-3.91848400	-1.03102900	-0.86926700
C	-4.13940600	1.06178400	0.30557200
N	0.06097100	1.37340700	-0.01709500
C	-4.72602400	-0.06264900	-0.27409500
N	1.09964200	1.68415000	0.40758600
H	-1.90030300	-1.61676200	-1.36785300
H	-2.30125400	2.09107900	0.76312500
H	-4.36560000	-1.90707500	-1.32779700
H	-4.76045500	1.82206400	0.76845800
H	-5.80437200	-0.18109800	-0.26492600

P12

C	1.71356800	-0.01522300	0.79085400
H	2.21359400	-0.21216900	1.74288300
C	0.42339400	-0.82284600	0.61275000
H	0.50374100	-1.47016400	-0.26545300
C	0.02450800	-1.61359200	1.85541900
H	-0.02867100	-0.95485200	2.72728300
H	-0.94760800	-2.09521400	1.72796600
H	0.76858500	-2.39153200	2.04863600
C	2.72247900	-0.20491300	-0.33477800
O	3.91249200	0.27907600	0.00693800
O	2.46949900	-0.71003200	-1.40638100
C	4.93097800	0.23422600	-1.00283000
H	5.12820100	-0.79917800	-1.29382700
H	4.61865600	0.80914200	-1.87641100
H	5.81181000	0.67949100	-0.54520000
C	-1.86110500	0.17454200	-0.01660800
C	-2.36201400	-1.04373200	-0.48687100
C	-2.71481500	1.27897800	0.09765500
N	-0.50514800	0.27932400	0.33150100

C	-3.70634900	-1.15340500	-0.83392200
C	-4.05146000	1.15522000	-0.26297300
N	0.06845700	1.47988300	0.50159600
C	-4.55920200	-0.05849300	-0.72677400
N	1.28583600	1.41421300	0.77826100
H	-1.71077800	-1.90409600	-0.59395600
H	-2.32694400	2.22197200	0.46295200
H	-4.08179200	-2.10469700	-1.19743200
H	-4.70353200	2.01829700	-0.17251600
H	-5.60509400	-0.14785600	-1.00042000

R13

C	-1.51086200	-1.20692200	-0.00001700
C	-0.11974600	-1.21119600	0.00002100
C	0.58796700	-0.00006700	0.00003600
C	-0.11962300	1.21114300	0.00001900
C	-1.51073300	1.20701900	-0.00001700
C	-2.20910600	0.00008200	-0.00003700
H	-2.05082700	-2.14831100	-0.00002800
H	0.42724900	-2.14821400	0.00002800
H	0.42747800	2.14810000	0.00002900
H	-2.05061000	2.14846000	-0.00002500
H	-3.29442100	0.00014500	-0.00006900
C	2.02329800	-0.00013200	0.00008600
C	3.23236100	-0.00011600	0.00003900
H	4.29978900	0.00094900	-0.00071400

TS13

C	2.16076100	-1.64669100	1.20935100
C	1.58191200	-0.38356400	1.28352000
C	1.78656200	0.54461500	0.25407800
C	2.57239700	0.18866000	-0.85080300
C	3.14305300	-1.07783200	-0.92013700
C	2.93881400	-1.99874300	0.10800000
H	1.99458300	-2.36035600	2.00985100
H	0.95964100	-0.11274700	2.13010300
H	2.72165600	0.90478200	-1.65211500
H	3.74851100	-1.34716900	-1.77972200
H	3.38418600	-2.98672900	0.04943400
C	1.17164800	1.84427700	0.32042600
C	0.92128400	3.04335100	0.49800300
H	1.16500300	4.03628300	0.81405200
C	-1.53947800	0.05352100	-0.38897200
C	-0.94608600	-1.12139500	-0.86006700
C	-2.74162500	-0.00478400	0.32631000
N	-0.82897600	1.24728200	-0.61885200
C	-1.55019900	-2.34873600	-0.60791200
C	-3.34514300	-1.23701000	0.55509500
N	-1.36346200	2.36419300	-0.39551200
C	-2.75185300	-2.41321400	0.09590600
N	-1.03390100	3.42723000	-0.04331100
H	-0.01205500	-1.06204400	-1.40901900
H	-3.20099600	0.90891700	0.69063300
H	-1.08072900	-3.25781500	-0.97001300
H	-4.28267900	-1.27726300	1.10064800
H	-3.22503500	-3.37153400	0.28307900

P13

C	-2.22244300	-1.92721800	-1.04528700
C	-1.32726800	-0.86482100	-0.97489400
C	-1.57467800	0.20830100	-0.11020100
C	-2.73351400	0.20417900	0.67344500
C	-3.63042700	-0.85817700	0.59589700
C	-3.37529200	-1.92727500	-0.26054800
H	-2.02177300	-2.75412700	-1.71877300
H	-0.43704300	-0.86565700	-1.59597600
H	-2.92412400	1.03044000	1.35148800
H	-4.52527100	-0.85237700	1.20983600
H	-4.07236900	-2.75711100	-0.31817600
C	-0.66032300	1.35803200	-0.03503100
C	-0.91394900	2.71257800	-0.04784300
H	-1.86295100	3.22233700	-0.11223900
C	1.55041500	0.15019700	0.11041200
C	1.31601700	-0.80988800	1.09126900
C	2.60790200	0.02647800	-0.78565000
N	0.69949700	1.29192800	0.02154400
C	2.14908400	-1.92237100	1.16153200
C	3.44421700	-1.08317900	-0.69631100

N	1.23884100	2.52758600	0.03886600
C	3.21289000	-2.06010000	0.27071400
N	0.26458000	3.38337300	-0.00548200
H	0.49244900	-0.68683300	1.78626700
H	2.76759700	0.79237100	-1.53637700
H	1.97111800	-2.67708800	1.92025300
H	4.27320800	-1.18592400	-1.38854700
H	3.86295200	-2.92666400	0.33271400

R14

C	-0.66091400	1.41701200	-0.36439500
H	-1.28738700	2.23965300	-0.69070600
C	0.66377200	1.55411900	-0.30702700
H	1.12222200	2.50257000	-0.57240100
C	-1.34830100	0.16046600	0.02294700
O	-2.65919700	0.23418300	-0.23086900
O	-0.80900500	-0.81077200	0.51317800
C	-3.43275700	-0.92305300	0.10893300
H	-3.08476900	-1.78969500	-0.45657600
H	-3.35385000	-1.12963000	1.17805100
H	-4.45801600	-0.67659700	-0.15970600
C	1.62110300	0.50511000	0.16914900
O	2.14103700	0.53850100	1.26250200
O	1.88512800	-0.39729200	-0.77145200
C	2.76779200	-1.46153400	-0.38930400
H	3.73652700	-1.06179200	-0.08372700
H	2.32661300	-2.02974600	0.43177800
H	2.87079400	-2.08445500	-1.27540200

TS14

C	0.70710100	0.17990000	-1.36344400
H	0.34209500	0.11885600	-2.38144700
C	1.90419400	-0.46041800	-1.06712700
H	2.32909600	-1.10787900	-1.82693300
C	0.21788400	1.40145500	-0.63993100
O	-0.11186900	1.17002200	0.62348500
O	0.09774300	2.47087900	-1.20060100
C	-0.57243000	2.29549500	1.37639100
H	0.21635700	3.04724600	1.44420000
H	-1.45856100	2.72683200	0.90609000
H	-0.81504300	1.90388200	2.36222500
C	2.87370700	0.03838200	-0.06395100
O	2.73395200	1.00994200	0.65175400
O	3.97801200	-0.72106400	-0.06486100
C	5.00295200	-0.33962900	0.85769000
H	5.34400600	0.67677900	0.65027000
H	4.63124500	-0.39622400	1.88292000
H	5.81105900	-1.05274800	0.70550700
C	-1.97607100	-0.89756800	-0.26884200
C	-2.78992100	-0.20577300	-1.16676800
C	-2.42094400	-1.16768900	1.02779400
N	-0.68568700	-1.24408700	-0.73260800
C	-4.04781200	0.22872800	-0.75969400
C	-3.68845100	-0.74671500	1.41525300
N	0.07162500	-1.97794400	-0.03619100
C	-4.50358500	-0.04233200	0.52912500
N	1.21250700	-2.12815200	0.13527100
H	-2.43220400	-0.00649500	-2.17172900
H	-1.77997000	-1.70079400	1.72242300
H	-4.67523000	0.77374900	-1.45728900
H	-4.03642000	-0.96334900	2.42017100
H	-5.48798900	0.29018700	0.84109900

P14

C	0.35606500	-0.22395400	-0.79649800
H	0.23330300	-0.19001500	-1.87989000
C	1.52760800	-1.15976000	-0.41324800
H	1.77045400	-1.82662800	-1.24435700
C	0.51752600	1.22263000	-0.31648700
O	-0.02555500	1.43472300	0.86971600
O	1.09631300	2.05082600	-0.98529000
C	0.15620400	2.74481000	1.42505200
H	1.22101000	2.93880100	1.56514600
H	-0.27722000	3.49676900	0.76338200
H	-0.36203800	2.72772900	2.38113100
C	2.79578900	-0.46649300	0.05860800
O	2.84211100	0.27346500	1.01412800
O	3.83541500	-0.78298700	-0.70646100
C	5.08737900	-0.17547400	-0.34994700
H	5.00338800	0.91121300	-0.40479300
H	5.37343900	-0.47360600	0.66004700
H	5.80532000	-0.54454200	-1.07899700
C	-2.08681500	-0.58896200	-0.17131200
C	-2.53076500	0.37111100	-1.08519500
C	-2.99772500	-1.19680800	0.70006700
N	-0.72712100	-0.94565800	-0.14893800
C	-3.87814900	0.72297200	-1.11960800
C	-4.33909300	-0.83634200	0.64909200
N	-0.25903200	-1.84682700	0.74281100
C	-4.79044100	0.12403300	-0.25642600
N	0.97487300	-1.99944600	0.67919700
H	-1.84173500	0.84860900	-1.77372400
H	-2.65277600	-1.93961700	1.40828600
H	-4.20940800	1.47173800	-1.83193600
H	-5.03765900	-1.31225700	1.32996500
H	-5.83888900	0.40052800	-0.28707400

R15

C	1.35949100	1.32759700	0.00000000
C	-0.01042300	1.08955300	0.00000000
C	-0.51170000	-0.22076400	0.00000000
C	0.40566500	-1.27988200	0.00000000
C	1.77857900	-1.04417400	0.00000000
C	2.26116200	0.26217000	0.00000000
H	1.72656800	2.34932900	0.00000000
H	-0.69227800	1.93411400	-0.00000100
H	0.03683700	-2.30234500	0.00000000
H	2.46956800	-1.88139900	0.00000000
H	3.32994700	0.45137000	0.00000000
C	-1.95545100	-0.53157000	0.00000000
H	-2.19076000	-1.59516800	-0.00000100
C	-2.97037200	0.33768400	0.00000100
H	-3.99671500	-0.01367100	0.00000000
H	-2.82487500	1.41408100	0.00000200

TS15

C	-0.39093000	2.54071400	1.26830600
C	-1.25299200	1.55743200	0.79451000
C	-1.14665900	1.08486700	-0.52198400
C	-0.14035400	1.61576700	-1.34020800
C	0.72431300	2.59747900	-0.86648300
C	0.60190600	3.06473300	0.44080800
H	-0.48701300	2.89154800	2.29119300
H	-2.00254200	1.14369500	1.46237000
H	-0.03051700	1.24478800	-2.35552900
H	1.49925900	2.99213400	-1.51616500
H	1.27843300	3.82685000	0.81443200
C	-2.00133800	0.00343900	-1.03737200
H	-1.79032500	-0.32545300	-2.04956600
C	-3.17764500	-0.41206200	-0.43477500
H	-3.85662700	-1.04529600	-0.99650700
H	-3.63115500	0.17688500	0.35434400
C	0.64252500	-1.45874600	-0.07930900
C	1.51187800	-1.43445200	-1.17271900
C	1.10467200	-1.09818800	1.19127200
N	-0.70867500	-1.73905800	-0.35902400
C	2.83726200	-1.05086400	-0.99639900
C	2.43423600	-0.72688600	1.35916500
N	-1.48186700	-2.07183700	0.59316300
C	3.30388800	-0.69650600	0.26874600
N	-2.59214600	-1.86460300	0.90396800
H	1.13445400	-1.71078900	-2.15200100
H	0.42382300	-1.10547800	2.03698700
H	3.50777100	-1.03148600	-1.84964800
H	2.79008600	-0.44955100	2.34641100
H	4.33859700	-0.39972100	0.40559200

P15

C	2.48480100	-1.14670100	1.61477700
C	1.59764600	-0.20846000	1.09461800
C	1.57557100	0.05608100	-0.27626300
C	2.44935300	-0.63140200	-1.11887000
C	3.34029900	-1.56931100	-0.59953300
C	3.35921100	-1.82905400	0.76912600
H	2.49063600	-1.34870300	2.68119100
H	0.91023100	0.31216600	1.75601700

H	2.43120400	-0.43703800	-2.18855300
H	4.01377700	-2.10034200	-1.26491700
H	4.04826900	-2.56271800	1.17520300
C	0.64671600	1.10899200	-0.84927300
H	0.49886600	0.92610400	-1.91838500
C	1.11331700	2.55881600	-0.59341100
H	1.11128100	3.17188100	-1.49877500
H	2.10661200	2.61174400	-0.14306400
C	-1.61762100	0.15701000	-0.12320200
C	-1.42859600	-1.02028800	-0.85617200
C	-2.78003700	0.31567300	0.64420800
N	-0.64862000	1.16715800	-0.17144800
C	-2.39365800	-2.02402600	-0.81862600
C	-3.73189000	-0.69664000	0.66919000
N	-0.81507100	2.32509500	0.51455000
C	-3.54967500	-1.87280300	-0.05879900
N	0.12275300	3.12627800	0.34340000
H	-0.53083500	-1.16551900	-1.44578700
H	-2.92990400	1.22641300	1.21045900
H	-2.23091100	-2.93201200	-1.39082400
H	-4.62763700	-0.56095200	1.26734300
H	-4.29764700	-2.65808200	-0.03203400

R16

C	-1.35233900	1.57845200	0.00005600
H	-2.36143500	1.97958000	0.00018600
H	-0.52940900	2.28388100	0.00008700
C	-1.14386100	0.25838700	-0.00008100
C	-2.24350400	-0.76581300	-0.00001600
H	-2.17134700	-1.41411200	-0.87833000
H	-2.17204700	-1.41325700	0.87897700
H	-3.22103900	-0.27916500	-0.00067200
C	0.24314900	-0.29961700	-0.00002200
O	0.48300000	-1.49174600	0.00006400
O	1.20237900	0.63485500	-0.00008200
C	2.54948000	0.15372500	0.00002300
H	2.73947500	-0.44789600	-0.89127000
H	3.17612700	1.04381900	0.00045200
H	2.73910500	-0.44853100	0.89096100

TS16

C	2.02315400	1.75657700	0.69696900
H	1.87799300	2.58616000	1.38071200
H	2.92591900	1.77599900	0.09727500
C	1.42176200	0.54070300	0.99156100
C	0.51343000	0.38221800	2.17986300
H	-0.22873800	-0.40277900	2.02224300
H	1.09826100	0.10572200	3.06392800
H	-0.00325000	1.32221700	2.38897400
C	1.95855200	-0.72078000	0.40685700
O	1.67262200	-1.83011500	0.81730400
O	2.79247200	-0.51431700	-0.61958200
C	3.30481900	-1.68611200	-1.25882700
H	2.48685200	-2.27993500	-1.67268900
H	3.95265100	-1.32370800	-2.05501300
H	3.87166400	-2.29273300	-0.54966800
C	-1.43975400	0.12346100	-0.45859700
C	-1.61240800	-1.25146100	-0.27396400
C	-2.53548600	0.98985000	-0.37354800
N	-0.11571700	0.56218900	-0.66267500
C	-2.87965000	-1.75479000	0.00119000
C	-3.80058200	0.47057500	-0.11792800
N	0.10165100	1.76322000	-0.99626300
C	-3.97826900	-0.89925900	0.07533100
N	0.90248600	2.58672400	-0.76740900
H	-0.74984900	-1.90699200	-0.33292300
H	-2.39783000	2.05668600	-0.51948500
H	-3.00870100	-2.82186700	0.15046600
H	-4.65117400	1.14261200	-0.06432800
H	-4.96689300	-1.29674900	0.27972400

P16

C	2.20775500	1.58995500	0.02694100
H	2.73601200	2.13703100	0.81048200
H	2.94705100	1.16740700	-0.65926900
C	1.23182300	0.53292300	0.60718600
C	1.05129800	0.68627500	2.11689200
H	0.29777000	0.00334100	2.51240300
H	1.99645600	0.48321800	2.62471100
H	0.74312900	1.71248300	2.33277500
C	1.70647000	-0.87412900	0.23451300
O	2.13332000	-1.67826500	1.03378500
O	1.62367400	-1.09019400	-1.07536000
C	2.05281300	-2.37884900	-1.53554800
H	1.44462200	-3.16075800	-1.07687200
H	1.90841500	-2.36387900	-2.61365400
H	3.10400500	-2.53790600	-1.28837900
C	-1.25163700	0.33564200	-0.05397500
C	-1.41991200	-0.92692000	0.52603300
C	-2.36827000	1.01063300	-0.56639200
N	0.02687900	0.91320600	-0.14040500
C	-2.68805400	-1.49877700	0.59603400
C	-3.62508800	0.42172700	-0.49583200
N	0.19430300	2.08540700	-0.80203300
C	-3.79856100	-0.83384800	0.08588000
N	1.35871500	2.51754600	-0.73900800
H	-0.57859700	-1.47742800	0.93111500
H	-2.24480500	1.98728700	-1.01661600
H	-2.79827500	-2.47720500	1.05279000
H	-4.47909400	0.95762700	-0.89819600
H	-4.78374300	-1.28444500	0.14104000

R17

C	1.32120900	-0.97325400	0.00000000
H	1.72782100	-1.97823700	0.00000000
H	2.01966500	-0.14321100	0.00000000
C	0.00000000	-0.78162500	0.00000000
H	-0.70109500	-1.61018500	0.00000000
C	-0.58096400	0.53048800	0.00000000
N	-1.06969400	1.58256900	0.00000000

TS17

C	1.54584800	1.38948600	-0.39872600
H	1.38004200	2.38555500	-0.00749700
H	1.00091100	1.12889500	-1.30042800
C	2.74451700	0.75072800	-0.10547100
H	3.47830400	1.21784900	0.54124700
C	3.22439900	-0.30575700	-0.94248900
N	3.61197100	-1.16544100	-1.62095900
C	-1.08476800	0.17210400	0.38607600
C	-1.90177900	1.21949000	-0.04234700
C	-1.52511300	-1.15139400	0.30315400
N	0.20679100	0.52983000	0.84927000
C	-3.16159300	0.93918000	-0.56228300
C	-2.79497400	-1.41645900	-0.20026500
N	0.92471200	-0.32899600	1.44261000
C	-3.61436100	-0.37709800	-0.63895100
N	2.04432600	-0.61422100	1.54251100
H	-1.54708800	2.24188200	0.03738200
H	-0.88452200	-1.96250700	0.63447800
H	-3.79432200	1.75409900	-0.89820700
H	-3.14048600	-2.44352500	-0.25662700
H	-4.60049300	-0.59213200	-1.03655200

P17

C	1.25692300	-0.82328000	-0.48263500
H	1.07247800	-1.38140400	-1.40159900
H	1.24643800	-1.50769800	0.37194600
C	2.53959000	0.02388700	-0.56102700
H	2.93540500	0.07838600	-1.58010900
C	3.61540700	-0.43906200	0.31787400
N	4.46146300	-0.82338900	1.00711500
C	-1.10065500	0.06668700	-0.10227400
C	-1.64245300	-1.21909200	-0.18288300
C	-1.93178100	1.16184200	0.16398900
N	0.27806800	0.24555500	-0.30597200
C	-3.01068600	-1.40530100	0.00670400
C	-3.29305900	0.95672100	0.35096900
N	0.86474400	1.43036900	-0.07599400
C	-3.84325000	-0.32358300	0.27443800
N	2.10785300	1.40567200	-0.17639400
H	-1.01159600	-2.07620800	-0.39096600
H	-1.50985400	2.15741100	0.22442900
H	-3.42051200	-2.40817900	-0.05651000
H	-3.93006900	1.81048200	0.55881200
H	-4.90740300	-0.47314900	0.42179700

R18

C	-0.54555100	0.38269700	0.00006500
H	-0.47433300	1.46571000	-0.00006700
C	0.54554700	-0.38264800	0.00020400
H	0.47430600	-1.46566300	0.00032300
C	-1.90024100	-0.22922800	0.00005400
O	-2.84753900	0.71323300	-0.00021600
O	-2.12987700	-1.42096400	0.00024700
C	-4.20259800	0.24673800	-0.00019800
H	-4.39541800	-0.35265500	-0.89207900
H	-4.39519800	-0.35333200	0.89127100
H	-4.81865600	1.14359300	0.00020200
C	1.90025200	0.22924200	0.00013900
O	2.12990800	1.42098100	0.00032300
O	2.84751200	-0.71323500	-0.00015100
C	4.20259400	-0.24679200	-0.00030500
H	4.39509200	0.35332100	-0.89176500
H	4.39555600	0.35253100	0.89159100
H	4.81860300	-1.14368000	-0.00085300

TS18

C	-1.89171800	0.58852100	0.14322700
H	-2.46687800	1.31722300	0.70345500
C	-0.63412400	0.92504200	-0.33472400
H	-0.22828400	0.40188900	-1.19458400
C	-2.61740200	-0.53027900	-0.49720400
O	-3.91579200	-0.51909800	-0.17874500
O	-2.10505000	-1.37168500	-1.21156600
C	-4.70509200	-1.58741100	-0.71334400
H	-4.68292700	-1.56743800	-1.80476100
H	-4.32997700	-2.54959200	-0.35879400
H	-5.71537200	-1.41303300	-0.34813800
C	-0.10229700	2.28553700	-0.04765100
O	-0.54354300	3.04094100	0.79245500
O	0.94081000	2.57201300	-0.83384500
C	1.55861500	3.84673500	-0.61904000
H	0.83991400	4.65046100	-0.78947200
H	1.94330400	3.91239700	0.40093400
H	2.37166300	3.90078200	-1.34048800
C	1.69807500	-0.81578600	0.49685000
C	2.82384600	-0.12681200	0.04575600
C	1.61927500	-2.20616700	0.38244800
N	0.63073700	-0.02812600	1.00515200
C	3.87736400	-0.83519800	-0.52389500
C	2.68720500	-2.90502000	-0.17035600
N	-0.24679700	-0.57750000	1.73703400
C	3.81504800	-2.22387300	-0.62825300
N	-1.40326100	-0.54877300	1.87419700
H	2.85910700	0.95275900	0.13959500
H	0.73363900	-2.73323600	0.72314400
H	4.75259600	-0.30053200	-0.87798800
H	2.63245400	-3.98557500	-0.25211400
H	4.64183000	-2.77439900	-1.06474400

P18

C	-1.69854700	-0.23781900	0.58052000
H	-2.11520100	0.40154300	1.36310600
C	-0.41687000	0.32825900	-0.05361500
H	-0.50473500	0.37700600	-1.14323000
C	-2.78673800	-0.55287300	-0.43857600
O	-3.94897700	-0.76305100	0.16559100
O	-2.60403700	-0.62232700	-1.63306800
C	-5.04340600	-1.15253700	-0.67881000
H	-5.24949000	-0.36991900	-1.41102300
H	-4.80547400	-2.08554400	-1.19241100
H	-5.89028300	-1.28694000	-0.00956200
C	-0.05678800	1.69417900	0.52022800
O	0.31272800	1.87378900	1.65699300
O	-0.23444500	2.65432100	-0.38433900
C	0.03862700	3.99441500	0.05461100
H	-0.61275900	4.25605500	0.89024100
H	1.08260600	4.08106800	0.36068700
H	-0.16496700	4.62794900	-0.80583800
C	1.89303600	-0.74647500	0.00664200
C	2.40941700	0.18781800	-0.89553800
C	2.73017000	-1.72438400	0.55699500
N	0.53555300	-0.68852900	0.36208000
C	3.75810800	0.14044500	-1.24286900
C	4.07074400	-1.76000700	0.19435300
N	-0.05044800	-1.68154000	1.05388500
C	4.59603400	-0.83041000	-0.70414300
N	-1.27265900	-1.51220000	1.22457000
H	1.77467600	0.94815200	-1.33802300
H	2.32857600	-2.44413500	1.25940400
H	4.14770800	0.87114200	-1.94434600
H	4.71227100	-2.52220800	0.62506100
H	5.64508200	-0.86381500	-0.97800700

R19

C	-2.48905300	-0.01337600	0.00016900
H	-3.42365200	-0.56369600	0.00017000
H	-2.53406200	1.07178700	0.00059100
C	-1.31608100	-0.64515800	-0.00017800
H	-1.24137800	-1.72770000	-0.00051400
C	-0.04128600	0.11499600	-0.00006500
O	0.06042200	1.32560200	-0.00015400
O	1.01284400	-0.71328900	0.00006300
C	2.30297000	-0.09294200	0.00009800
H	3.02025900	-0.91151200	0.00046500
H	2.42688400	0.52548100	-0.89125600
H	2.42651800	0.52602200	0.89112500

TS19

C	0.67289800	-1.44866000	-0.87216200
H	0.32917600	-2.46859500	-0.99235300
H	0.26818100	-0.70868200	-1.55653500
C	1.89720400	-1.20734900	-0.27425700
H	2.49683400	-2.01821500	0.12233400
C	2.56724400	0.08548200	-0.49890600
O	2.04213000	1.07459000	-0.97939100
O	3.84689200	0.05755700	-0.09578000
C	4.57718400	1.27905900	-0.23581600
H	5.57519800	1.06748400	0.14411300
H	4.62342900	1.57930500	-1.28478600
H	4.10673200	2.07328900	0.34776700
C	-1.87509600	-0.31951700	0.29757500
C	-2.77068000	-0.96220900	-0.55897600
C	-2.14919600	0.96538600	0.77373100
N	-0.68049300	-1.01843400	0.59836100
C	-3.94071700	-0.31450600	-0.94277000
C	-3.33198000	1.59453700	0.39794500
N	0.08034300	-0.59613600	1.51799300
C	-4.22844300	0.96245800	-0.46288500
N	1.21373900	-0.54662000	1.76305300
H	-2.54453000	-1.96197100	-0.91505900
H	-1.44598900	1.46397800	1.43317300
H	-4.63363000	-0.81470100	-1.61146900
H	-3.54777300	2.58916400	0.77445000
H	-5.14543400	1.46200600	-0.75698000

P19

C	0.41270300	-0.70145400	0.78499400
H	0.13914400	-1.10493700	1.76242600
H	0.48678600	-1.51226400	0.05306400
C	1.68383100	0.14568100	0.82456400
H	2.08823500	0.28444400	1.82986600
C	2.79389900	-0.36741500	-0.08253100
O	2.63015000	-1.15341600	-0.98905000
O	3.96025700	0.18429100	0.23588100
C	5.07328800	-0.15305600	-0.60490900
H	5.92029700	0.39437100	-0.19702100
H	5.25721500	-1.22847000	-0.57285500
H	4.87361300	0.15570800	-1.63249700
C	-1.91213500	0.09683100	0.09929500
C	-2.45730100	-1.17506700	0.30242300
C	-2.73191200	1.14473800	-0.34105400
N	-0.55038900	0.30609100	0.35002000
C	-3.81313900	-1.39324300	0.06539600
C	-4.08088200	0.90781900	-0.57348400
N	0.03800100	1.48249100	0.08412200
C	-4.63315800	-0.35824900	-0.37289500
N	1.26421400	1.48902300	0.31947200
H	-1.83452300	-1.99581000	0.64048500
H	-2.30909100	2.12935000	-0.49783000
H	-4.22379000	-2.38504200	0.22584200

H	-4.70723700	1.72575600	-0.91543200
H	-5.68786300	-0.53284200	-0.55679500

R20

C	2.63092200	-0.41158500	-0.00003300
H	3.66661600	-0.67400500	0.00037000
C	1.46187500	-0.11724400	0.00006400
C	0.07241100	0.30309600	-0.00007600
O	-0.27874700	1.46235400	-0.00000300
O	-0.75188700	-0.74350700	-0.00014600
C	-2.15435900	-0.43381300	0.00010200
H	-2.41342500	0.13884000	-0.89218400
H	-2.66002400	-1.39689800	0.00002500
H	-2.41318600	0.13856800	0.89263200

TS20

C	2.94712000	-0.99174300	-0.46089000
H	3.84664300	-1.44240300	-0.82595100
C	2.19301300	-0.06453100	-0.14870600
C	1.64042400	1.25706000	0.09643100
O	1.75981700	1.86974400	1.13567200
O	0.96636500	1.69543800	-0.96880100
C	0.33360600	2.97530800	-0.82702500
H	1.07813800	3.74624800	-0.62070200
H	-0.15722400	3.15882600	-1.78045900
H	-0.39977300	2.94257100	-0.01879700
C	-0.89604400	-0.80026500	0.37423200
C	-1.39889300	0.28279100	1.09776200
C	-1.67050400	-1.41774000	-0.61248200
N	0.44132400	-1.16559600	0.65002600
C	-2.67801000	0.75673100	0.82308000
C	-2.95479400	-0.94678900	-0.86334500
N	0.89860900	-2.27770100	0.29289400
C	-3.46025800	0.14332400	-0.15407500
N	1.89318100	-2.73597700	-0.10444600
H	-0.78200100	0.74224900	1.86330000
H	-1.27189700	-2.25790800	-1.17241900
H	-3.06719800	1.60080500	1.38318500
H	-3.56090100	-1.43032300	-1.62260200
H	-4.46100000	0.50852000	-0.35972700

P20

C	1.94770000	-1.84351100	0.13804900
H	3.01641000	-1.98794800	0.13753500
C	1.23242800	-0.66891100	0.04817600
C	1.68559000	0.72241400	-0.13790800
O	0.96609600	1.66663400	-0.37965200
O	3.01282500	0.79501900	-0.01866100
C	3.59061300	2.09345900	-0.21374100
H	3.20184200	2.79450200	0.52698400
H	4.66176800	1.95370000	-0.08501000
H	3.36735800	2.45870200	-1.21779900
C	-1.28002100	-0.30668600	0.05491400
C	-1.56752200	0.55849900	1.10447300
C	-2.15510800	-0.48018800	-1.01044300
N	-0.06857900	-1.07111100	0.08233400
C	-2.75725300	1.27821900	1.07547100
C	-3.34769400	0.23898600	-1.02529300
N	-0.13852500	-2.40212700	0.18672000
C	-3.64669400	1.11894900	0.01294100
N	1.07643500	-2.87428300	0.21638400
H	-0.86780700	0.66720700	1.92590800
H	-1.90176500	-1.16702000	-1.81042600
H	-2.99182100	1.95969400	1.88616000
H	-4.03993600	0.11262900	-1.85084200
H	-4.57496300	1.68057500	-0.00390800

R21

C	0.59981800	-0.19596400	0.06359500
C	-0.59983800	-0.19576300	-0.06356900
C	-2.03681600	-0.24763800	-0.27767400
O	-2.55774800	-0.97960300	-1.08809200
O	-2.67389000	0.60051200	0.52228000
C	-4.10585600	0.62754400	0.39302400
H	-4.52101400	-0.35473500	0.62447200
H	-4.44287100	1.36888100	1.11380200
H	-4.38484000	0.91792300	-0.62120000
C	2.03687800	-0.24849800	0.27706900
O	2.55813500	-0.98315000	1.08485200
O	2.67365900	0.60232800	-0.52027800
C	4.10568600	0.62888100	-0.39150800
H	4.44246500	1.37233400	-1.11021700
H	4.38508500	0.91626100	0.62345500
H	4.52068800	-0.35273200	-0.62602700

TS21

C	0.82292600	0.62065900	-0.17671900
C	1.89084600	0.03385400	0.03615100
C	3.27056500	-0.10023500	0.48371800
O	3.59205500	-0.21074700	1.64639000
O	4.11586000	-0.09760200	-0.54570400
C	5.50568700	-0.23627800	-0.21362600
H	5.67719400	-1.18534500	0.29756500
H	6.03130400	-0.21172000	-1.16576500
H	5.82556700	0.58913400	0.42481900
C	-0.11388900	1.73784200	-0.27602600
O	-0.27579200	2.40350500	-1.27426900
O	-0.77535400	1.90048800	0.86757700
C	-1.77022100	2.93572900	0.87610600
H	-2.19315600	2.91701100	1.87804200
H	-1.31100700	3.90332800	0.66706000
H	-2.53805800	2.71969600	0.13045900
C	-1.82780700	-0.96688100	-0.42487700
C	-2.72875600	-0.12611500	-1.07838100
C	-2.23465800	-1.74940900	0.65864500
N	-0.48181100	-0.90809900	-0.86770600
C	-4.04720100	-0.06222900	-0.63689900
C	-3.55936600	-1.69123100	1.07718900
N	0.31627200	-1.85067500	-0.63909200
C	-4.46594800	-0.84486700	0.43752000
N	1.42843400	-2.03644100	-0.37207000
H	-2.38804400	0.46833800	-1.91977400
H	-1.52317100	-2.39400500	1.16524500
H	-4.74950500	0.59307800	-1.14156100
H	-3.88229300	-2.30327800	1.91288200
H	-5.49631000	-0.79991000	0.77435000

P21

C	1.52150200	-0.80180700	-0.06199700
C	0.38291300	-0.02669800	-0.11746300
C	0.21345200	1.45518000	-0.22070500
O	-0.05025600	2.02275000	-1.25511000
O	0.38685300	2.03362700	0.95688000
C	0.32013200	3.46984800	0.96877800
H	-0.66108700	3.80217900	0.62601100
H	0.48574100	3.75554600	2.00487100
H	1.09935600	3.87956700	0.32412400
C	2.90875500	-0.30433200	-0.09407200
O	3.17326500	0.87624600	-0.21443200
O	3.80785500	-1.27407900	0.02093700
C	5.18162300	-0.86393100	-0.00789000
H	5.75733100	-1.78197300	0.08895800
H	5.40721200	-0.36519600	-0.95229300
H	5.39135700	-0.18814500	0.82334000
C	-2.04043900	-0.69027500	-0.05853600
C	-2.62674400	0.06227500	-1.07190700
C	-2.79400200	-1.24552600	0.97191700
N	-0.62863800	-0.91827100	-0.07258300
C	-4.00243800	0.27533500	-1.03692800
C	-4.16949800	-1.03665400	0.98551800
N	-0.14034000	-2.17792400	0.01491300
C	-4.77278800	-0.27325700	-0.01350300
N	1.14780100	-2.10677000	0.01765600
H	-2.01853800	0.47021500	-1.87133500
H	-2.30513200	-1.82913800	1.74386500
H	-4.47120700	0.86255000	-1.81920200
H	-4.76826600	-1.46557300	1.78179600
H	-5.84507000	-0.10857100	0.00449500

TS8*

C	-0.04774600	-1.50767400	1.40630700
H	-0.27154800	-0.53520700	1.83107900
H	-0.51408000	-2.37386800	1.85601400
C	1.09580800	-1.68369300	0.65887100
H	1.56799600	-2.65944100	0.57327800
O	1.87756400	-0.60274200	0.38792500
C	3.07049700	-0.89328000	-0.33828400

H	2.81116700	-1.42562700	-1.26107000
H	3.71058300	-1.54603500	0.27387900
C	3.78324600	0.40779400	-0.65186700
H	4.66293300	0.16189600	-1.26081100
H	3.12979100	1.02893500	-1.27727800
C	4.21747700	1.19285900	0.58641100
H	3.33333400	1.44247500	1.18278700
H	4.84870900	0.55176300	1.21589600
C	4.97731500	2.46910400	0.22852200
H	5.26966700	3.02291900	1.12574900
H	5.88803100	2.24082500	-0.33666300
H	4.35997500	3.13165100	-0.38820200
C	-3.31774300	-0.11574200	1.01800200
C	-4.21396600	0.94409300	1.10572400
C	-4.30284600	1.88044300	0.07606900
C	-3.47895600	1.75180700	-1.04152400
C	-2.56560600	0.70564900	-1.13334400
C	-2.48791300	-0.23624000	-0.10109700
H	-3.25296300	-0.85753000	1.80746400
H	-4.85300900	1.03183400	1.97868000
H	-5.01087900	2.69992500	0.14147200
H	-3.54044700	2.47548000	-1.84824500
H	-1.92206400	0.61092200	-2.00214100
N	-1.57473900	-1.31047300	-0.08529500
N	-0.87015800	-1.55257600	-1.11481700
N	0.21416600	-1.94690800	-1.31389100

TS9*

C	2.64441900	0.13656500	-0.24036600
C	2.98046600	-0.93684200	-1.07177300
C	3.28735000	0.29186900	0.99333900
N	1.62483300	0.98923300	-0.70678000
C	3.94608400	-1.85192000	-0.66633500
C	4.26296600	-0.62239700	1.38020200
N	1.29743900	2.00778600	-0.02701400
C	4.59477800	-1.69898700	0.55888200
N	0.31962800	2.60999300	0.18430500
H	2.47940500	-1.04315100	-2.02852700
H	3.03063200	1.12738900	1.63672400
H	4.19808700	-2.68430300	-1.31575200
H	4.76330900	-0.49180800	2.33465900
H	5.35325400	-2.40988500	0.86931100
C	-0.39386700	0.34675600	-1.24255300
H	-0.24887500	-0.62515000	-0.77824500
H	-0.15886600	0.41632400	-2.29808100
C	-1.17256000	1.30485600	-0.62678300
H	-1.56271600	2.11106200	-1.24617900
C	-1.91253100	1.04109000	0.66274300
H	-1.93289700	1.95740000	1.26246600
H	-1.36189400	0.29293100	1.24837800
C	-3.35806700	0.56096200	0.44946300
H	-3.85668400	0.55316500	1.42571200
H	-3.89493000	1.29644000	-0.16635900
C	-3.47160700	-0.81950700	-0.19824600
H	-2.93369900	-0.81310300	-1.15397100
H	-2.96124800	-1.55730600	0.43800400
C	-4.91234500	-1.27855300	-0.44761300
H	-5.41379100	-0.55019500	-1.09875800
H	-4.88700400	-2.22231000	-1.00556400
C	-5.73718300	-1.47620100	0.82537700
H	-5.88921400	-0.53463800	1.36233000
H	-6.72551700	-1.88511000	0.59351500
H	-5.23806800	-2.17298200	1.50873100

TS15*

C	2.29515400	2.04307500	-0.21389700
C	1.56963600	0.86653000	-0.37233600
C	2.21223700	-0.38027900	-0.35963700
C	3.59802100	-0.41171800	-0.16078800
C	4.32506200	0.76527800	-0.00068800
C	3.67707100	1.99890300	-0.02928300
H	1.77809200	2.99778200	-0.22629500
H	0.49062200	0.92165000	-0.49323600
H	4.11039500	-1.36982700	-0.13584500
H	5.39958900	0.71796700	0.14752000
H	4.24176300	2.91718500	0.09704300
C	1.46217100	-1.64287600	-0.52398100
H	2.02069900	-2.55990300	-0.35904300
C	0.26897600	-1.71918200	-1.22051500
H	-0.10133500	-2.68602700	-1.53987200
H	-0.11128800	-0.86191800	-1.76780600
C	-2.14073700	-0.46335800	0.19573700
C	-3.05464600	-0.55419400	-0.85748900
C	-2.16501000	0.64223300	1.05289500
N	-1.18251700	-1.49917200	0.28814400
C	-3.98476900	0.46134900	-1.05484100
C	-3.11249700	1.64253400	0.85629100
N	-0.42333300	-1.53246700	1.31081700
C	-4.02137300	1.56102700	-0.19793400
N	0.69903900	-1.74752200	1.53457000
H	-3.02660600	-1.42009300	-1.51104100
H	-1.45249300	0.71310400	1.86904000
H	-4.68991400	0.38729200	-1.87649100
H	-3.13380400	2.49398400	1.52903800
H	-4.75381200	2.34699900	-0.34892500

TS17*

C	-3.18385100	0.15233700	-0.07109900
H	-3.97880800	-0.15566300	0.59891000
H	-3.44097400	0.23263900	-1.12074200
C	-2.14582500	0.91740400	0.44618500
H	-2.10146600	1.16016200	1.50067100
C	-1.35265300	1.74495700	-0.41363000
N	-0.69775000	2.40893000	-1.10497200
C	0.70424400	-0.48698800	0.38833400
C	1.45881400	0.31962300	1.24185700
C	1.26453000	-0.99551700	-0.78717100
N	-0.64866900	-0.68597600	0.75006300
C	2.77709200	0.62009200	0.91622300
C	2.58967300	-0.70349700	-1.09240400
N	-1.33044800	-1.57386700	0.16422800
C	3.34773800	0.10652300	-0.24750000
N	-2.42036500	-1.70034700	-0.24402700
H	1.00373100	0.70699900	2.14750200
H	0.67176300	-1.61926500	-1.44886900
H	3.36117100	1.25207700	1.57696200
H	3.02829000	-1.10457600	-2.00024700
H	4.37833900	0.33684900	-0.49599800
